# Supplementary material for: The structure and predictive value of intrinsic capacity in a longitudinal study of ageing
Source: BMJ Open. 2019 Nov 2;9(11):e026119. doi: 10.1136/bmjopen-2018-026119 (PMC6830681; doi:10.1136/bmjopen-2018-026119)

Supplementary analysis

Table 1s : Sample characteristics

**Demographic and health characteristics**	**Wave 4**
**Age , mean (SD)**	70.5 (7.9)
**Sex**	
Male	3181(44.7%)
Female	3925 (55.3%)
**Education**	
No education	2035(30.7%)
Intermediate	2527(38.2%)
Higher education	2053(31.0%)
**Wealth quintile**	
1 (lowest)	712 (12.9%)
2	1119(20.3%)
3	1192 (21.6%)
4	1184(21.4%)
5 (highest)	1302(23.6%)
**Multimorbidity**	
0	1681(25.1%)
1 or 2	3647(54.3%)
3 or more	1381(20.6%)
**Activities of daily living**	
No ADL limitation	5745(80.8%)
1 or 2 limitations	1082(15.2%)

3 or more limitations	280 (3.9%)
**Instrumental activities of daily living**	
No limitation	5582(78.5%)
1 or 2 limitations	1228 (17.3%)
3 or more limitations	297 (4.2%)

Table 2s : Model fit statistics of ESEM and CFA models

**Models**	**Fit statistics**				
	**χ2**	**df**	**CFI**	**TLI**	**REMSEA (90% CI)**
**ESEM**					
One-factor	5385.6	104	0.63	0.57	0.136 (0.133 -0.139)
Two-factors	2570.8	89	0.82	0.76	0.101(0.098- 0.104)
Three-factors	927.5	75	0.94	0.90	0.064(0.061-0.068)
Four- factors	277.4	62	0.98	0.97	0.036(0.031-0.040)
Five-factors	117.9	50	0.99	0.98	0.022(0.017-0.030)
Six-factors (one general factor and five sub-factors) [1]	71.2	39	0.99	0.99	0.012(0.011-0.024)
**CFA**					
One-factor	6735.9	104	0.56	0.49	0.154(0.150-0.150)
Second-order	2369.9	102	0.94	0.92	0.073(0.070-0.080)
Correlated five factors	1782.3	103	0.95	0.92	0.060(0.050-0.060)
Bi-factor (one general factor and five sub-factors)	1180.6	89	0.98	0.97	0.035(0.033 -0.037)

[1] *Bi-factor exploratory analysis is conducted in SEM framework.*

Table 3s: Construct validity of intrinsic capacity and sub-domain score

Demographic and health characteristics	Intrinsic capacity<br><br>Regression coefficient (95% CI)	Sensory<br><br>Regression coefficient (95% CI)	Cognitive<br><br>Regression coefficient (95% CI)	Vitality<br><br>Regression coefficient (95% CI)	Psychological<br><br>Regression coefficient (95% CI)	Locomotor<br><br>Regression coefficient (95% CI)
**Age**	-0.052 (-0.054 to - 0.046)***	-0.002(-0.008 to 0.003)	-0.02(-0.023 to -0.019)***	-0.021(-0.024 to -0.019)***	-0.003(-0.005 to -0.0003)*	-0.009(-0.011 to -0.007)***
**Sex**						
Male	Ref 1	Ref 1	Ref 1	Ref 1	Ref 1	Ref 1
Female	-0.322 (-0.358 to -0.286)***	0.036(-0.037 to 0.110)	0.27(0.240 to 0.302)***	-0.881(-0.905 to -0.857)***	-0.251 (-0.314 to -0.189)***	0.099(0.059 to 0.139)***
**Education**						
No education	Ref1	Ref 1	Ref 1	Ref 1	Ref 1	Ref 1
Intermediate	0.462(0.420 to 0.504)***	0.085(0.037 to 0.133)***	0.267(0.230 to 0.304)***	0.068(0.029 to 0.106)***	0.027(-0-0.017 to 0.073)	0.058(0.028 to 0.094)***
Higher education	0.779(0.735 to 0.823)***	0.114(0.064 to 0.164)***	0.4042)***	0.239 (0.198 to 0.279)**3	-0.015(-0.062 to 0.032)	0.063(0.031 to 0.094)***
**Wealth quintile**						
1 (lowest)	Ref 1	Ref 1	Ref 1	Ref 1	Ref 1	Ref 1
2	-0.020(-0.085 to 0.044)	0.045(-0.025 to 0.116)	-0.072(-0.128 to -0.169)*	-0.072 (-0.132 to -0.129)*	-0.045(-0.113 to 0.230)	0.049(0.003 to 0.095)**
3	0.268(0.204 to 0.332)***	0.006(-0.150 to	0.050 (-0.005 to	0.016(-0.042 to	-0.017(-0.152 to	0.125(0.080 to 0.171)***

		0.163)	0.105)	0.075)	0.116)	
4	0.448(0.384 to 0.512)***	0.016(-0.141 to 0.164)	0.111(0.056 to 0.166)***	0.073 (0.014 to 0.131)*	-0.0530 (-0.119 to 0.0139)	0.155(0.110 to 0.200)***
5 (highest)	0.616(0.553 to 0.678)***	0.01(-0.142 to 0.159)	0.201 (0.147 to 0.255)***	0.099(0.041 to 0.156)**	0.055 (-0.074 to 0.184)	0.181(0.137 to 0.226)***
**Multimorbidity**						
0	Ref 1	Ref 1	Ref 1	Ref 1	Ref 1	Ref 1
1 or 2	-0.0237 (-0.279 to -0.195)***	-0.068(-0.151 to 0.014)	-0.012(-0.049 to 0.025)	-0.122(-0.160 to -0.085)***	0.035(-0.010 to 0.077)	-0.032(-0.061 to -0.003)*
3 or more	-0.764(-0.816 to -0.712)***	-0.057 (-0.151 to 0.014)	-0.067(-0.114 to -0.021)**	-0.308 (-0.354 to -0.261)***	-0.242(-0.350 to -0.133)***	-0.221(-0.251 to -0.185)***
**Activities of daily living**						
No ADL limitation	Ref 1	Ref 1	Ref 1	Ref 1	Ref 1	Ref 1
1 or 2 limitations	--0.471(-0.510 to -0.432)***	-0.105(-0.147 to 0.063)***	-0.049(-0.082 to -0.016)**	-0.096 (-0.131 to -0.061)***	-0.134 (-0.187 to -0.082)***	-0.097(-0.122 to -0.072)***
3 or more limitations	-0.857(-0.918 to -0.797)***	-0.116(-0.182 to -0.050)***	-0.088(-0.082 to -0.016)**	-0.072(-0.127 to -0.018)***	-0.028(-0.357 to 0.299)	-0.317 (-0.356 to -0.279)***
**Instrumental activities of daily living**						
No limitation	Ref 1	Ref 1	Ref 1	Ref 1	Ref 1	Ref 1
1 or 2 limitations	-0.636(-0.677 to -0.596)***	-0.097(-0.142 to -	-0.077(-0.111 to -	-0.127(-0.164 to -	-0.105(-0.253 to	-0.147 (-0.173 to -

		0.053)***	0.042)***	0.091)***	0.043)	0.121)***
3 or more limitations	-1.067(-1.144 to -0.990)***	-0.108(-0.193 to -0.023)**	-0.396 (-0.462 to -0.330)***	-0.107(-0.177 to -0.036)**	0.273(-0.108 to 0.656)	-0.259 (-0.309 to -0.209)***

*** p value < 0.001, **p value <0.05.

Table 4s: Regression coefficient of direct of intrinsic capacity on ADL and IADL and indirect effect through multimorbidity

Causal path	ADL		IADL	
	**Standardized Coefficient (SE)1**	**P value**	**Standardized Coefficient (SE)1**	**P value**
**Total effect**				
Intrinsic capacity	**-0.52 (0.01)**	**<0.001**	**-0.48(0.02)**	**<0.001**
**Direct effect**				
Intrinsic capacity	**-0.40(0.03)**	**<0.001**	**-0.39(0.02)**	**<0.001**
**Indirect effect**				
Intrinsic capacity→Multimorbidity	**-0.039(0.005)**	**<0.001**	**-0.024(0.005)**	**<0.001**
	**R2=0.20, pvalue=<0.001**		**R2= 0.21, pvalue=<0.001**	

[1]Controlled for age, sex, education, and wealth

Table 5s: Direct of and indirect effect personal characteristics on ADL and IADL in serial multiple mediators (intrinsic capacity and multimorbidity)

	ADL		IADL	
**Causal path**	**Standardized Coefficient (SE)**	**P value**	**Standardized Coefficient (SE)**	**P value**
**Direct effect**				
Age	0.006(0.02)	0.011	0.110 (0.03)	0.001
Sex	-0.049(0.03)	0.061	-0.049(0.02)	0.051
Education	0.009(0.01)	0.571	-0.012(0.02)	0.613
Wealth	-0.013(0.01)	0.185	0.016(0.24)	0.510
Multimorbidity	0.036(0.01)	0.001	0.020(0.02)	0.402
Intrinsic capacity	-0.099(0.02)	<0.000	-0.142(0.02)	<0.001
**Specific indirect effect**				
Age → Intrinsic capacity	0.053(0.01)	<0.001	0.050(0.01)	<0.001
Age → multimorbidity	0.011(0.04)	0.004	0.003(0.01)	0.405
Female → intrinsic capacity	0.047(0.010)	<0.001	0.048(0.01)	<0.001
Female → multimorbidity	0.008(0.003)	0.008	0.002(0.003)	0.409
Education→intrinsic capacity	--0.022(0.01)	<0.001	-0.02(0.01)	0.001
Education→multimorbidity	0.000(0.002)	0.810	0.00(0.00)	0.817
Wealth →intrinsic capacity	-0.021(0.01)	<0.001	-0.021 (0.01)	<0.001
Wealth→multimorbidity	-0.009(0.01)	0.007	-0.002(0.001)	0.408
**Indirect effect**				
Age → Multimorbidity → Intrinsic capacity	0.002(0.001)	0.002	0.002(0.001)	0.002
Female → Multimorbidity → Intrinsic capacity	0.002(0.001)	0.004	0.002(0.001)	0.004
Education→ Multimorbidity → Intrinsic capacity	0.000(0.00)	0.810	0.000(0.00)	0.810
Wealth → Multimorbidity → Intrinsic capacity	-0.002(0.001)	0.003	-0.002(0.001)	0.003

Figure 1s: A statistical diagram of a simple mediation model (Direct and indirect) effect of intrinsic capacity on ADL and IADL

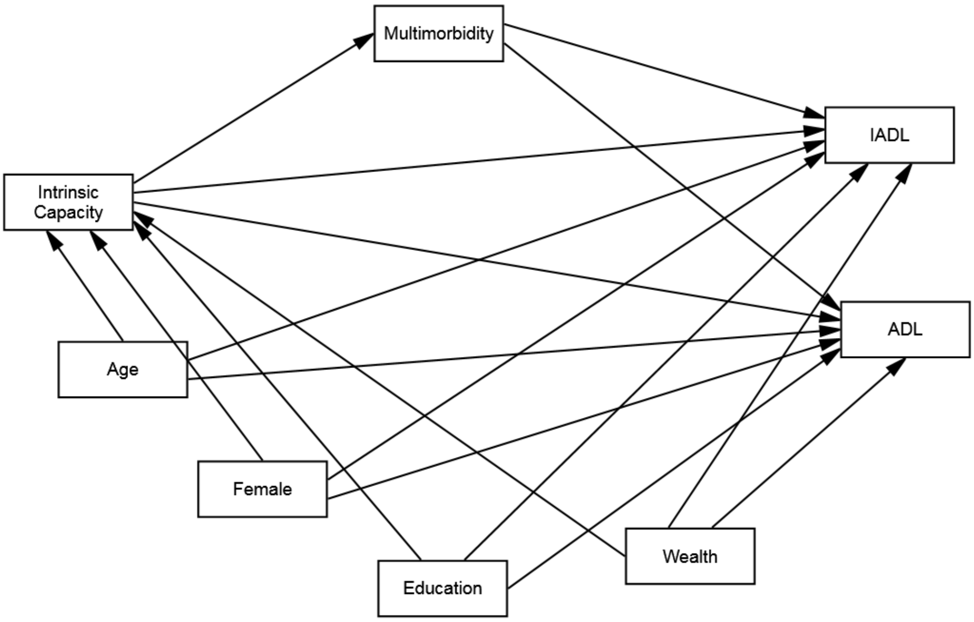

Table 6s: Model fit information for Parallel and Sequential models examining pathways to ADL and IADL

**Models**	**Fit statistics**					**R2**		
**Activities of daily livings**	χ2	df	CFI	TLI	REMSEA (90% CI)	**Multi morbi dity**	**Intrinsi c capacity**	**ADL**
Model 1a : Effect of age (and other covariates) on ADL is either mediated by multimorbidity or intrinsic capacity	43.9	5	0.96	0.89	0.06(0.04-0.08)	7.6%	42.6%	18.0%
Model 1b : Effect of age(other covariates) on ADL is mediated by multimorbidity and intrinsic capacity	16.3	4	0.98	0.96	0.04(0.022 - 0.062)	7.6%	45.5%	18.9%
**Instrumental Activities of Daily Livings**								
Model 2a: Effect of age (and other covariates) on IADL is either mediated by multimorbidity or intrinsic capacity	55.6	5	0.95	0.86	0.07(0.05-0.09)	7.8%	42.7%	35.4%
Model 2b : Effect of age(and other covariates) on IADL is mediated by multimorbidity and intrinsic capacity	30.9	4	0.97	0.91	0.05(0.04-0.08)	7.9%	45.4%	31.2%
Model 2c : Effect of age(and other covariates) on IADL is mediated by intrinsic capacity (with no direct path between multimorbidity and IADL) model)	493	4	0.99	0.99	0.05(0.03-0.06)	11.5%	42%	32%

Figure 2s : Interaction effect of age and intrinsic capacity on incidence IADL.

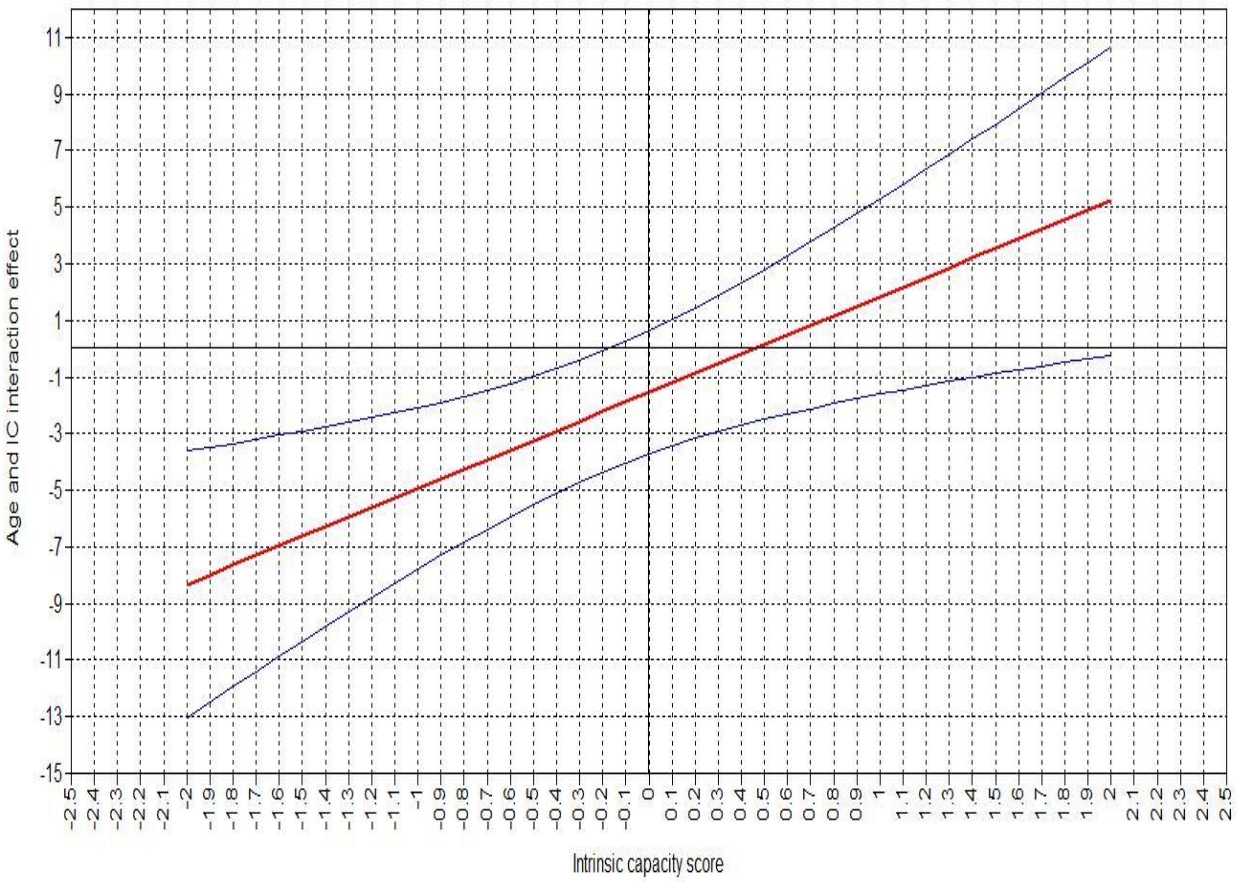

Supplement: Supplementary data [file bmjopen-2018-026119supp001.pdf]
