# Supplementary material for: The structure and predictive value of intrinsic capacity in a longitudinal study of ageing
Source: BMJ Open. 2019 Nov 2;9(11):e026119. doi: 10.1136/bmjopen-2018-026119 (PMC6830681; doi:10.1136/bmjopen-2018-026119)
Supplement: Author's manuscript [file bmjopen-2018-026119.draft_revisions.pdf]

BMJ Open is committed to open peer review. As part of this commitment we make the peer review history of every article we publish publicly available.

When an article is published we post the peer reviewers' comments and the authors' responses online. We also post the versions of the paper that were used during peer review. These are the versions that the peer review comments apply to.

The versions of the paper that follow are the versions that were submitted during the peer review process. They are not the versions of record or the final published versions. They should not be cited or distributed as the published version of this manuscript.

BMJ Open is an open access journal and the full, final, typeset and author-corrected version of record of the manuscript is available on our site with no access controls, subscription charges or pay-per-view fees (http://bmjopen.bmj.com).

If you have any questions on BMJ Open's open peer review process please email info.bmjopen@bmj.com

BMJ Open

The structure and predictive value of intrinsic capacity in a longitudinal study of ageing

Journal:	*BMJ Open*
Manuscript ID	bmjopen-2018-026119
Article Type:	Research
Date Submitted by the Author:	17-Aug-2018
Complete List of Authors:	Beard, John; Organisation mondiale de la Sante, Ageing and Life Course<br>Jotheeswaran, AT; Organisation mondiale de la Sante, Department of Ageing and Life Course<br>Cesari, Matteo; Università di Milano<br>Araujo de Carvalho, Islene; Organisation mondiale de la Sante
Keywords:	EPIDEMIOLOGY, GERIATRIC MEDICINE, PUBLIC HEALTH, PRIMARY CARE

SCHOLARONE™
Manuscripts

Article type : Original research paper

Title : Intrinsic capacity in a longitudinal study of ageing

John Beard[1],Jotheeswaran Amuthavalli Thiyagarajan[1], Matteo Cesari[2], Islene Araujo de Carvalho[1]

[1]Department of Ageing and Life Course, World Health Organization, Geneva, Switzerland
[2]Department of Medical Sciences and Community Health, Università di Milano, Milano, Italy.

Corresponding author: John Beard, Department of Ageing and Life Course, World Health Organization, Geneva, Switzerland. Email ID: beardj@who.int

ABSTRACT:

Objectives: To test the construct and predictive validity of the World Health Organization concept of intrinsic capacity in relatively robust participants of a large longitudinal study of ageing; to identify whether this overall measure disaggregated into biologically plausible and clinically useful subdomains; and to assess whether intrinsic capacity predicted subsequent development of care dependence.

Design: Structural equation modeling of commonly used biomarkers and self-reported measures in the English Longitudinal Study of Ageing including exploratory factor analysis, exploratory bi-factor analysis and confirmatory factor analysis. Longitudinal mediation and moderation analysis of the direct and indirect relationships of intrinsic capacity and multimorbidity with incident loss of ADLs and IADLs.

Settings: Community, United Kingdom

Participants: 2560 eligible participants aged over 60 years

Main outcome measures: Activities of daily living (ADL) and instrumental activities of daily living (IADL).

Results: One general factor (intrinsic capacity) and five sub-factors emerged: locomotor, cognitive; psychological; sensory; and "vitality". This factor structure is consistent with biological theory and the model had a good fit for the data. The summary score of intrinsic capacity and specific sub-factors showed good construct validity in relation to age, sex, education, wealth, and multimorbidity. In a causal path model examining incident loss of ADL and IADL, intrinsic capacity had a direct relationship with the outcome and was a strong mediator for the effect of age, sex, wealth and education. Multimorbidity had an independent direct relationship with incident loss of ADLs but not IADLs, and also operated through intrinsic capacity. More of the indirect effect of personal characteristics on incident loss of ADLs and IADLs was mediated by intrinsic capacity than multimorbidity. In interaction tests, intrinsic capacity moderated the direct effect of chronological age on IADL and ADL.

Conclusions: The WHO construct of intrinsic capacity appears to provide valuable predictive information on an individual's subsequent functioning, even after accounting for the number of multimorbidities. The proposed general factor and sub-domain structure may contribute to a transformative paradigm for future research and clinical practice.

For peer review only

Strength and limitations of this study

1. To our knowledge this is the first large population-based longitudinal analysis to examine the structure and predictive validity of the WHO concept of intrinsic capacity.
2. We applied a rigorous psychometric approach for constructing a valid measurement model using commonly measured biomarkers and self-reported measures, allowing us to create a theoretically error-free composite score for intrinsic capacity, which was used in all analysis.
3. We used longitudinal data to minimize the potential for reverse causality and adjusted for multimorbidity to minimise confounding-by-disease; however, the potential of residual confounding cannot be completely eliminated.
4. This study shows that many of the commonly used assessments of health and functioning in older age have common variance (i.e. they are possibly measuring one underlying trait of an individual's health status) that is consistent with the WHO concept of intrinsic capacity.
5. This composite measure was structured in a way that is consistent with biological theory.
6. However, it is important to note that the measures included in the ELSA study are neither complete nor random. They were chosen to inform specific research questions of interest to the investigators, rather than to create an overall measure of intrinsic capacity.

INTRODUCTION

In 2015, the World Health Organization released the *World report on ageing and health*, which proposed a public health framework for action on population ageing[1 2]. Central to the Report is a new conceptual model for "*Healthy Ageing*". Rather than considering healthy ageing from the perspective of the presence or absence of disease, this functioning-based approach is oriented around building and maintaining the ability of older people to be, and to do, the things they have reason to value. The Report proposes that this "functional ability" is determined by the "intrinsic capacity" of the individual, the environments in which they live and the interaction between the individual and these environments. However, while the Report considers intrinsic capacity to be "all the physical and mental capacities" that an individual can draw on at any point in time, it does not provide a detailed description of the components of capacity, how they might be structured or how capacity and its components may be measured and monitored.

This reframing of the concept of healthy ageing builds on a growing body of research exploring patterns and determinants of functional status in older people. Many of these studies examine functioning in areas such as physical performance or cognition[3 4], and increasingly they are applying a life course perspective.[5] At the same time there is growing interest in the biological underpinnings of ageing and in identifying ways to measure "biological" age as distinct from chronological age.[6] This work all serves to better capture the heterogeneity that is a hallmark of ageing and helps researchers and clinicians advance from stereotypical notions of older age, and towards more personalised interventions to foster healthy ageing.

There has also been significant work identifying measures that might assess different domains of functioning at different stages in life.[7] However, there is less research and less agreement on how functional approaches for specific domains might together reflect the *overall* health status of older individuals.[8 9] It also remains unclear how specific functional domains such as locomotor and cognitive capacity relate to each other, and how the deficits in the complex and dynamic biological systems that underpin ageing relate to these more overt expressions of an individual's capacity.[10]

For peer review only

Broad self-reported measures of health and wellbeing such as the SF36 and GHQ attempt to capture this heterogeneity, but do not consider key capacities (for example cognitive capacity), and can have difficulty distinguishing between the contribution of individual or environmental level factors to functional status.

Distinguishing between capacity and ability is also a problem for other commonly used measures of overall functioning in older age including Instrumental Activities of Daily Living (IADLs) or Activities of Daily Living (ADLs). Losses of IADLs and ADLs are also generally only observed with very significant decrements of functioning,[11] while the WHO model suggests that changes in capacity are likely to start much earlier in life. Understanding the factors that influence levels and trajectories of overall capacity in relatively robust people before they experience these significant losses may help identify interventions earlier in the life course, and could be useful in self-care and clinical practice. Broad based outcomes like this could be useful in other ways too - for example as a way of comparing the relative benefits of interventions on different functional domains or in different organ systems.

Continuous measures of intrinsic capacity that are sensitive to subtle changes and that distinguish between the individual and their context would thus enable a much better understanding of functioning at both a population and individual level. However, this would first require a clearer conceptualisation of the intrinsic capacity construct.

To progress work in this area, we examined data from the English Longitudinal Study on Ageing (ELSA) to assess whether a range of commonly collected biomarkers and self-reported measures might provide a useful estimate of intrinsic capacity, and whether this construct predicted subsequent outcomes in relatively robust older people after accounting for the number of health conditions a participant may be experiencing. We examined the factor structure of the total capacity score to identify relevant sub factors and used structural equation modelling to assess longitudinally the direct and indirect relationships of the total intrinsic capacity score, personal characteristics and multimorbidity with subsequent IADL or ADL loss.

METHODS

Study description:

The English Longitudinal Study of Ageing (ELSA) is an ongoing study of a nationally representative sample of the English population aged ≥50 years[12]. Participants were recruited from households that were included in the Health Survey for England in 1998, 1999 and 2001, and then followed up every 2 years with detailed health examinations through nurse visits taking place every 4 years. Data were collected via face-to-face assessments using computer-assisted personal interviews and a self-completion questionnaire. In addition, a trained nurse visited participants in waves two, four, and six to measure physical functioning and collected the blood samples which were then analyzed to generate biomarker data. In ELSA the response rates varied across the waves with 67 % in wave 1, 82 % in wave 2, 73 % in wave 3, 74 % in wave 4 and 80 % in wave 5 [12]. The inclusion criteria for the present study include a) participants aged over 60 years included in the nurse visit, b) consent to provide blood sample, c) no missing data on main exposure (intrinsic capacity) indicators, and d) follow-up outcome data available in wave 5 (2010/2011). Applying these criteria led to a total study sample of 2352 participants (Figure 1).

Patient involvement: All participants were required to provide informed written consent. All ELSA data are anonymous and freely accessible from the UK Data Service Discover. Only data contained within the ELSA database were included in the analyses. No patients were involved in the development of the research question, study design or interpretation of the data in this study.

For peer review only

Figure 1: Flow of study members into the analytical sample: the English Longitudinal Study of Ageing.

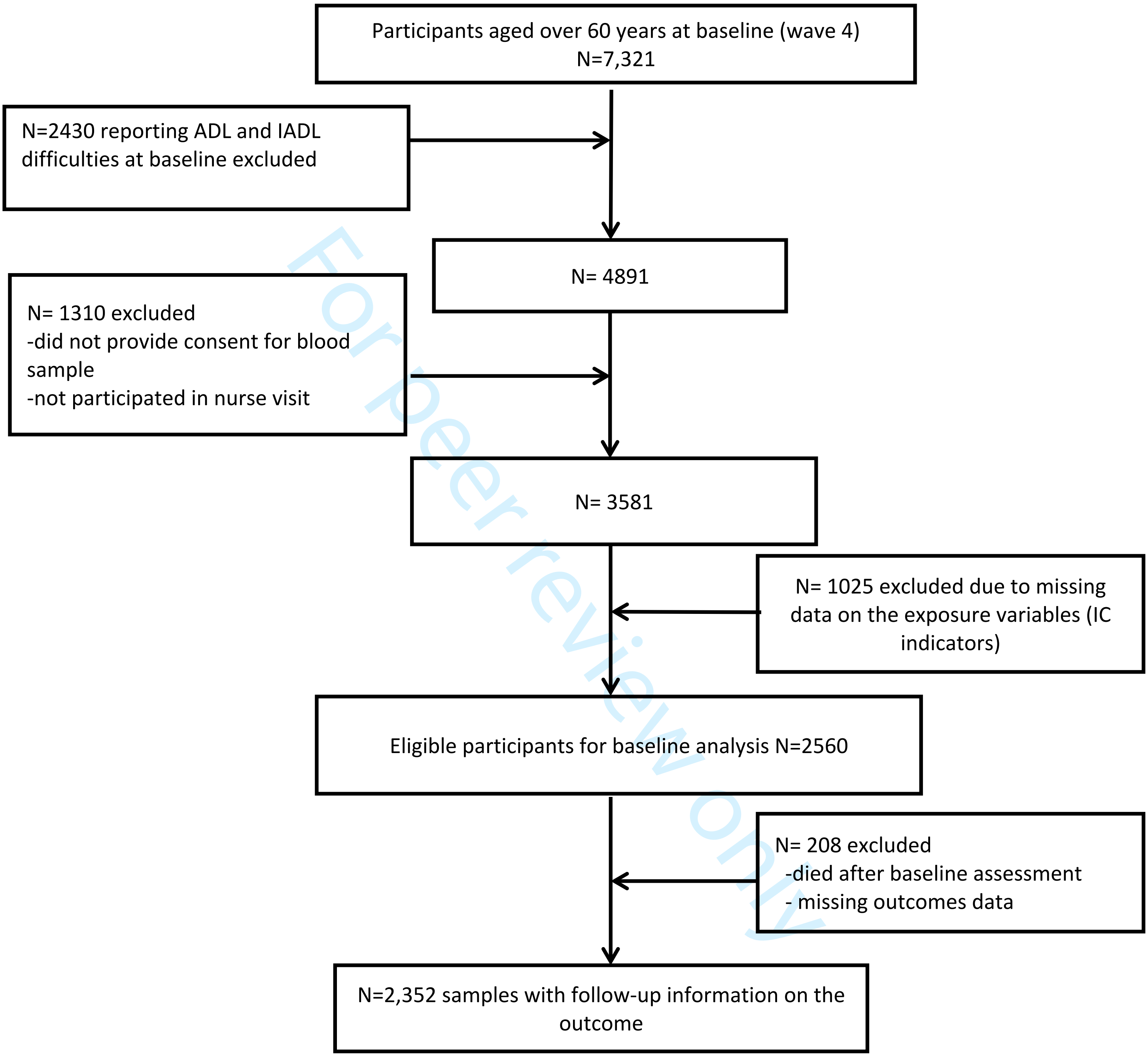

For peer review only

Measures

Intrinsic capacity:

We considered measures collected in ELSA that might provide objective estimates of aspects of intrinsic capacity based on the following criteria: a) prior evidence supporting an association with at least one aspect of

capacity, and b) ability to distinguish between high and low physical or mental capacity at older ages and sensitivity to detect change within and between individuals over time.

Walking speed: Each participant aged 60 and above was eligible for the *timed walk* test. In addition, prior to the actual test, respondents were asked if they had any problems from recent surgery, injury, or other health conditions that might prevent them from walking. Only persons aged at least 60 years, willing to do the test, and able to walk (walking aids were permitted) were asked to walk 8 feet (2.4 m) at their usual walking pace, twice[13]. The time for both walks was recorded separately. In our analysis we use the mean speed (measured in m/s) of the two trials.

Chair-stand test: The chair stand test, a measure of physical performance, assessed the time required to rise from a chair to a full standing position five times with arms folded across the chest, with slower times reflecting worse function[14]. The test incorporated the use of respondent's own armless, straight backed chair. The time taken for full stand was recorded in seconds. Respondents were considered as ineligible if they could not stand up without assistance; the use of walking aids, such as a walker or cane, was not permitted. The test was stopped if the respondent became too tired or short of breath; if the participant used their hands; if after one minute, the participant had not completed all the rises; or if the nurse felt concerned for the respondent's safety.

Balance: Static balance was evaluated in three separate and progressively more difficult tests which formed part of the Short Physical Performance Battery[15]. Participants were ineligible for the tests if they were chair-bound or wheelchair-based; if it became clear after discussion that they were too unsteady on their feet; if they found it painful to stand; or if either the nurse or the participant considered the test unsafe. We used three components of the balance test (an additional two components were performed by younger participants only): side-by-side, semi-tandem, and full tandem. A) Side-by-side stand: Participants were asked to stand with feet together, side-by-side, for at least 10 seconds, using their arms, bending their knees or moving their body to maintain balance, but not moving their feet. If the participant was unable to hold the position for 10 s, a score of zero was recorded and no further tests attempted. Those able to hold the position for 10 s moved on to the semi-tandem stand. B) Semi-tandem stand: Participants had to stand with the side of the heel of one foot touching the big toe of the other foot for at least 10s. Participants unable to hold the position for 10 s scored one and no further tests were attempted. Those able to hold the position for 10 s moved on to the full-tandem stand. C) Full-tandem stand: For this test, participants had to stand with the heel of one foot in front of and touching the toes of the other foot. Those unable to hold this position for at least 3s scored no additional points; those able to hold the position for at least 3 but less than 10 s scored one point for this test; and those able to hold the position 10 s or longer scored two points for this test. The maximum possible score from all three tests was four points: one point each from the side-by-side and semi- tandem tests, and two points from the full-tandem test.

Grip strength: The grip strength test is a test for upper body strength[16]. Handgrip strength (kg) of the dominant hand was assessed using a handheld dynamometer, with the average(mean) of three measures used in the analyses. Three values were recorded for each hand, starting with the non-dominant hand and alternating between hands. Any measurements carried out incorrectly or participants refused to perform the test were not included.

Forced expiratory volume: Lung function was measured using a NDD Easy On Spirometer[17]. Willing and eligible respondents were asked to stand or seated, take a deep breath and blow into the spirometer as hard as they could. Respondents were then required to repeat the procedure to give three technically satisfactory blows. The highest technically satisfactory measure of forced expiratory volume in 1 second (FEV1) was used in the analysis. The protocol required three successful measurements to be completed. An unsatisfactory blow included any of the following: an unsatisfactory start with excessive hesitation; laughing or coughing, especially during the first second; a Valsalva manoeuvre; leakage of air around the mouthpiece; obstruction of the mouthpiece by tongue or teeth; obstruction of the spirometer flow head outlet by hands.

For peer review only

Blood assay: A trained nurse collected biomarker data from all participants not meeting exclusion criteria. Viable blood samples were obtained from 6188 respondents (75.6% of wave 4 participants). Detailed information on the technicalities of the blood analysis, the internal quality control and the external quality assessment for the laboratory have been described elsewhere[18]. Dehydroepiandrosterone DHEA (S) levels from serum was performed using the Roche DHEA(S) assay that is a competitive immunoassay using electrochemiluminescence technology (analytical range: 0.003–27 µmol/L)[19]. Haemoglobin level (g/dl) was measured with two Abbott Diagnostics Cell-Dyn 4000 analysers[20]. IGF-1(Insulin-like growth factor 1) values are reported as whole numbers (range: 3–200 nmol l−1)[21].

Sensory: Hearing and vision impairments were measured using self-reported [22 23], validated questions previously demonstrated to be accurate when compared with objective measures. Hearing status was assessed by asking participants to rate their hearing (using a hearing aid if they used one) as excellent, very good, good, fair, or poor. For vision, participants were also asked 'How good is your eyesight for seeing things at a distance, like recognising a friend across the street' and 'How good is your eyesight for seeing things up close, like reading ordinary newspaper print'. Response options (excellent/very good/good/fair–poor) were categorised as above.Cognitive*:* The ELSA data include scores on three tests of cognitive function: verbal fluency, delayed verbal memory, and attention[24]. Verbal (semantic) fluency was assessed by asking participants to name as many animals as they could think of in 1 minute. Delayed verbal memory was assessed using lists of nouns presented aurally. Attention was assessed using a letter cancellation task. Scores on these tests were used as measures of three kinds of cognitive function: the scores on the animal naming task were taken as a measure of executive function[25], the sum of the scores on the delayed recall tasks were taken as a measure of memory, and the scores on the letter cancellation task were taken as a measure of processing speed[26].

Affect*:* Affect was measured using the eight-item Center for Epidemiological Studies-Depression (CES-D) scale[27]. Five of the eight CES-D items (i.e. felt depressed, was happy, felt lonely, enjoyed life, felt sad) were depressed mood items, while the remaining three (i.e. everything was an effort, restless sleep, and could not get going) were somatic complaints items. We derived a summary CES-D score by adding responses to all eight dichotomous questions (possible range:0-8).

Sleep: To assess sleep disturbance, participants were asked about the frequency of delay in falling asleep, inability to stay asleep, waking up tired, and disturbed sleep in the previous month[28]. Response categories were no difficulties, less than once a week, once or twice a week and three times or more a week. These response codes were given a numerical score (1 to 4) and then items were summed and a total score created. The total score ranged between 4 and 16, and showed a normal distribution, with a mean score of 8.8 (standard deviation 3.2).

Other covariates:

We also extracted data on other sociodemographic and medical covariates, recorded at wave 4, that may potentially confound the associations between intrinsic capacity and care dependence. These included chronological age, sex, education (no education, intermediate and higher education), total non-pension net wealth in quintile as a proxy measurement of socioeconomic status and multimorbidity (self-reported information on doctor diagnosed diabetes, hypertension, stroke, heart diseases (myocardial infarction, congestive heart failure, angina), chronic obstructive pulmonary disease , asthma, arthritis, osteoporosis, cancer, Parkinson's disease, Alzheimer's disease and other dementia[29].

Measures of outcome:

Care dependence: The outcome of interest for longitudinal analysis – incident care dependence - was chosen because it was an overall measure of functioning that was assessed independently from the functional characteristics included in the intrinsic capacity construct. Care dependence was assessed using self-reported

For peer review only - http://bmjopen.bmj.com/site/about/guidelines.xhtml

limitations in the Basic Activities of Daily Living (ADL) and Instrumental Activities of Daily Living (IADL)[30]. Respondents were asked to exclude any difficulties expected to last less than 3 months. ADL included six activities: dressing, walking across a room, bathing or showering, eating, getting in or out of bed, using the toilet. IADL included seven activities: using a map to get around in a strange place, preparing a hot meal, shopping for groceries, making telephone calls, taking medications, doing work around the house or garden and managing money. The scales ranging from 0 to 6 for ADL and 0 to 7 for IADL (number of items with reported difficulty) were constructed. To enable us to identify the incident loss of ADLs and IADLs, adults with limitations at wave 4 were excluded from the baseline analysis.

Statistical analysis

All statistical analysis was performed using Mplus version 8 [31] and Stata 14[32]. We performed incrementally related structural equation models (SEMs): a) traditional exploratory factor analysis, b) exploratory bi-factor analysis(EFA), c) confirmatory factor analysis(CFA), and d) mediation and moderation analysis.

We first performed a conventional exploratory factor analysis to reveal sub-factors of the intrinsic capacity concept using the robust weighted least squares (WLSMV) method. Eigen value and scree plot were used to identify number of sub-factors to retain. Communalities ≥0.3 was selected for minimum loading of an item. We then conducted a bi-factor analysis to examine the possibilities of establishing one general factor (Intrinsic capacity). The bi-GEOMIN rotation was implemented that allowed specific sub-factors to be correlated with the general factor (intrinsic capacity) and also correlated with each other. The factor structure was further tested in the confirmatory factor analysis. We identified the best fitting model using the inferential goodness-of-fit index in combination with several descriptive indices: root mean square error of approximation (RMSEA), comparative fit index (CFI), Tucker–Lewis Index (TLI). CFI and TLI values of greater than 0.9 and a RMSEA of less than 0.8 suggest a moderate fit, where as a CFI and TLI of greater than 0.95 and a RMSEA of less than 0.6 suggest a very good fit[33]. For the bifactor model, we calculated omega hierarchical coefficients (ωH), because in the bifactor model the indicators are assumed to be influenced by both the general factor and the specific factors[34].

We tested the construct validity of the general factor(intrinsic capacity) and specific sub-factors in regression analysis. The summary scores for general factor and specific sub-factors were generated from CFA by fixing the latent mean to 0 and the latent standard deviation to 1 for each factor. The scores of specific sub-factors can be interpreted as the unique contribution of each of the specific domains "over and above" the general factor (intrinsic capacity). These summary scores were used in the linear regression for testing the construct validity. Simple t-test were performed to examine the statistical difference in the intrinsic capacity score among older persons with or without chronic diseases and results are summarized by age-group and overall population score in two-way boxplot.

Finally, we assessed the predictive validity of the intrinsic capacity score in a mediation model of the direct and indirect relationships of intrinsic capacity and multimorbidity with incident loss of ADLs and IADLs, after controlling for all personal characteristics[35]. PM (ratio of the indirect effect to the total effect) and Rm (ratio of the indirect effect to the direct effect) was calculated to examine the indirect effect size in the mediation analysis[36 37]. For visualizing moderation effects, we used the Johnson-Neyman technique[38]. A bias-corrected bootstrap method was used for drawing inference in mediated and moderated analysis [35].

For peer review only - http://bmjopen.bmj.com/site/about/guidelines.xhtml

RESULTS

Sample characteristics

Baseline levels of study variables are presented in supplementary table 1s (online). Of the 7321 potential participants at baseline, 33% reported either ADL or IADL difficulties and 26 % did not provide consent for blood sample analysis (Figure 1). A further 28% of the remaining 3581 participants, participants had incompleteinformation on the independent variables and were also excluded from analysis. The baseline sample therefore comprised 2560 eligible participants. Compared to participants included in the baseline analysis, participants without complete information were older, had a lower education attainment and reported more chronic conditions.

In the follow-up, 91% of baseline eligible participants were re-interviewed. Except education, there was no difference on age, sex, wealth, and multimorbidity status among participants interviewed and not interviewed at the follow-up (Table s1).No imputation was performed in the analysis and participants with missing data were excluded, leaving a study sample of 2560 with complete data for the EFA and CFA analysis.

Bi-factor EFA, CFA and model fit

In the initial exploratory factor analysis, the Kaiser eigenvalues criterion suggested a five-factor model, with 5 factors having Eigen values greater than 1 (i.e.3.1,2.3,1.61, 1.23,1.04). These five factors explained 86% of total variance among the intrinsic capacity indicators. Supplementary table 2s shows the model fit information for EFA and CFA models tested in the study. One to three factor models provided unacceptable degrees of fit to the data, whereas five factor models provided very good fit, which suggests that intrinsic capacity is a multidimensional construct.

Next, we performed bi-factor EFA under a SEM framework to identify potential modelling problems (e.g. sizable cross loading of intrinsic capacity indicators) and get an early insight on whether primary results of EFA could be replicated with bi-factor model perspectives of multidimensionality. Most items loaded well (≥0.3) on the general factor (intrinsic capacity). Bi-factor EFA revealed one general factor (IC) and five specific sub-factors that we labelled cognitive, sensory, vitality, locomotor, and psychological (supplementary table 3s). The model fits the data very well: chi-square = 71.2 (df = 39), RMSEA = 0.012 (90% CI 0.011 to 0.024), CFI = 0.99 and TLI = 0.99 (Supplementary table 2s). When we examined the factor structure (one factor, second-order, correlated, bi-factor models) in confirmatory factor analysis, the pattern of factor loadings for the bi-factor CFA model showed a clear, simple structure with the five sub-factors (Figure 2).

For peer review only

Figure 2: Bi-factor CFA model of Intrinsic Capacity

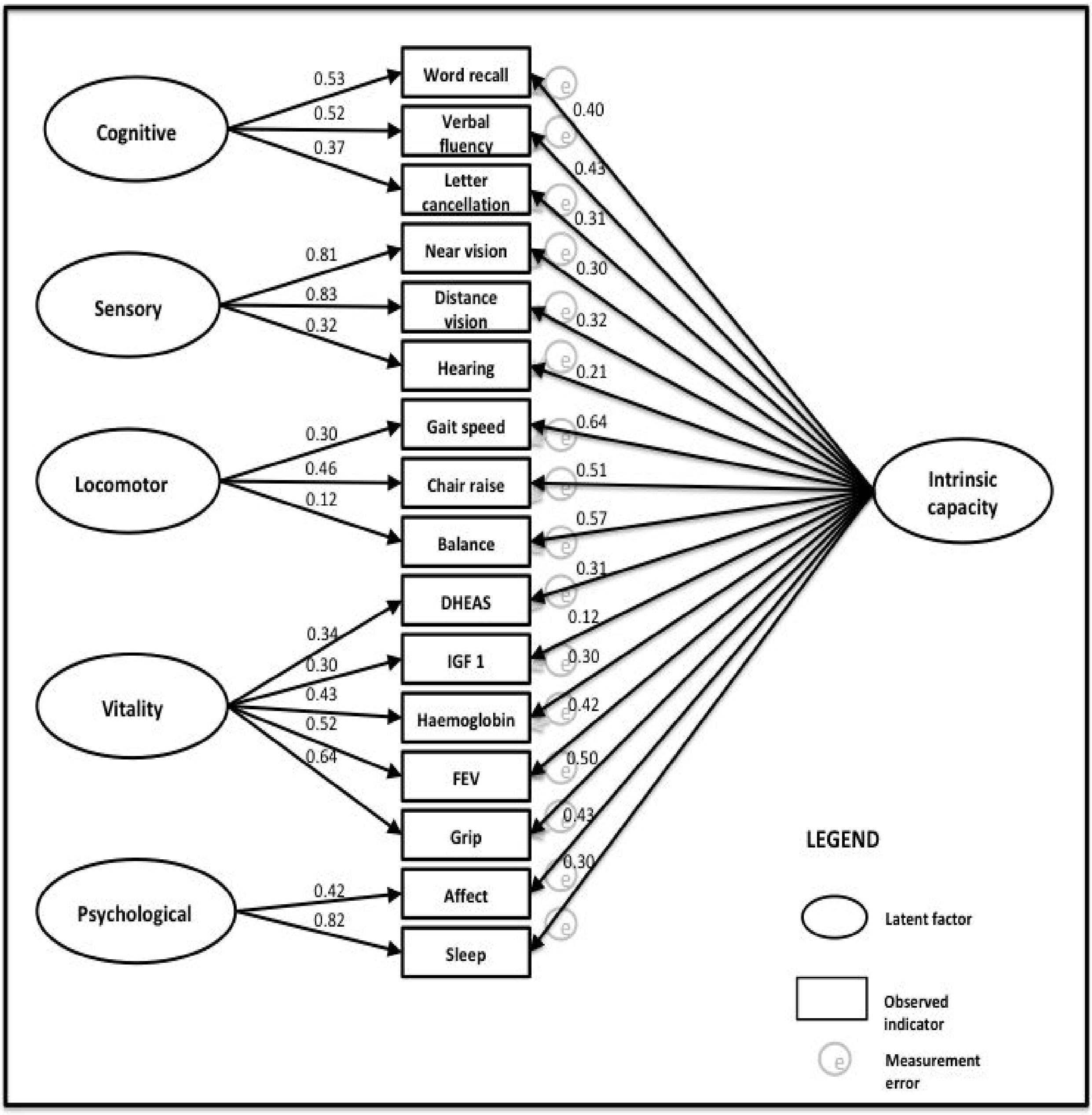

Within the bifactor CFA model, excluding two sub-factors (sensory and locomotor), the factor loadings were evenly shared between the general factor and sub-factors. However, indicators in the psychological (sleep) and sensory (near vision and distance vision) sub-factors had higher loadings on their group factor than on the general factor (intrinsic capacity). This suggests that these two sub-factors provide additional information about psychological and sensory capacity, after accounting for the variance of the general factor. The model achieved a good fit for the data: chi-square value =1180.6(df=89), RMSEA =0.035 (90% CI 0.033 to 0.037), CFI =0.98 and TLI=0.97(table 2s). Indeed, the bi-factor model fit was stronger than for the second order factor model: chi-square value =2369 (df=102), RMSEA =0.07, CFI =0.94 and TLI=0.92. Taken together, these findings support this bi-factor model with one general factor representing overall intrinsic capacity and five specific sub-factors.

Reliability of the factor scores

The ωH (hierarchical) coefficient was calculated to understand the reliability of a latent general factor (Intrinsic capacity). The ω̇h value for the general factor was 0.78, and the ωHS(sub-score) values for specific factors were .0.79, 0.80,0.81,0.82, and 0.83, respectively. A ωH value more than 0.7 indicates that the intrinsic capacity total score predominantly reflects a single general factor, suggesting that the total score can be interpreted as a reliable measure of intrinsic capacity. The ωHS more than 0.80 for the sub-factor suggests that domain specific scores are equally reliable as the general factor score. Independent of specific factors, the percentage of reliable variance in the score due to the general factor was 72%. This indicates that the intrinsic capacity summary score was a sufficiently reliable measure of the general factor, and added value beyond sub-factor scores.

Construct validity

Factors associated with intrinsic capacity (general factor) and sub-domains (sub-factors) are presented in the supplementary table 4s. Lower intrinsic capacity scores were significantly associated with increasing age, female sex, lower levels of education, lower wealth, number of chronic diseases, and number of ADL and IADL limitations. Even after mutual adjustment, all related constructs remained statistically associated with intrinsic capacity (see Figure 3). Since all these characteristics have previously been associated with poorer health in older age, these findings support the construct validity of the general factor.

Figure 3: Construct validity of Intrinsic capacity (mutually adjusted)

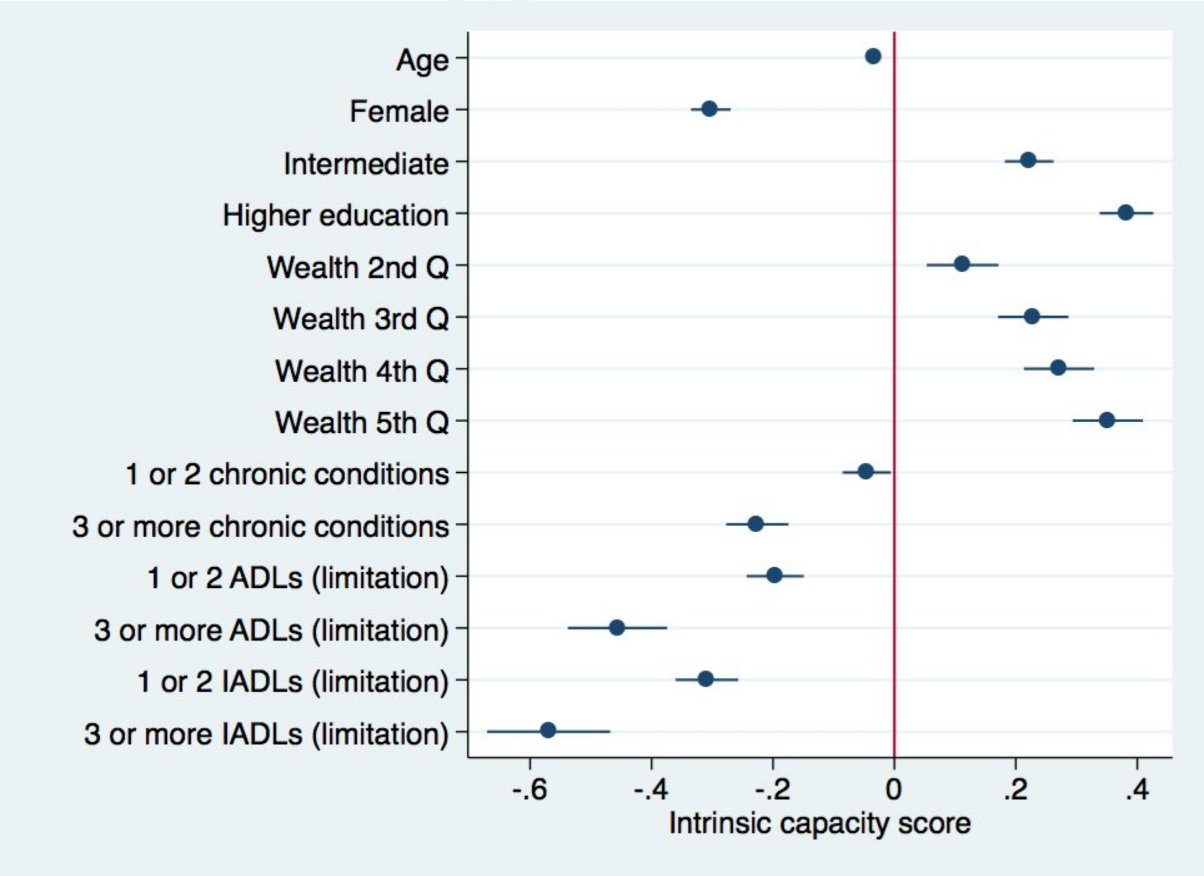

Associations between intrinsic capacity score and other variables

We used a boxplot of intrinsic capacity score for each chronic condition over three different age group to display associations between specific chronic conditions and intrinsic capacity s (Figure 4). Overall, older adults with chronic conditions had statistically significantly lower intrinsic capacity scores (below the mean) than those without chronic conditions and this association was stronger in older age groups. However, the impact of different chronic conditions on the intrinsic capacity scores varied. The greatest impact on intrinsic capacity score was from dementia in the two older age groups. We also examined the intrinsic capacity scores among older people with no chronic conditions in different age-groups. We found that in the absence of any diagnosed chronic conditions, the intrinsic capacity scores tend to decline in higher age-groups. In other words, older people with no diagnosed chronic conditions in higher age-groups (70-79 and 80-100) had significantly lower intrinsic capacity scores than older people in young age-group 60-69 years.

In a separate correlation analysis, we found associations between specific factor scores and various personal characteristics or multimorbidity and these associations were generally consistent with previous research on these characteristics (table 4s). Cognitive factor scores were negatively associated with increasing age, number of multimorbidities and positively associated with female sex, higher education, and wealth (highest quantile). Locomotor scores were negatively associated with age and multimorbidity, and positively associated with higher education, wealth, and female sex. Psychological factor scores were negatively associated with increasing age and higher multimorbidity. Higher psychological factor scores were negatively associated with age, female sex and multimorbidity. Vitality sub-factor scores were negatively associated with increasing age and multimorbidity, and positively associated with female sex, higher education and higher wealth. The scores of the sensory sub-factor were positively associated only with higher education.

Figure 4: Intrinsic capacity summary score by chronic health conditions and age-group

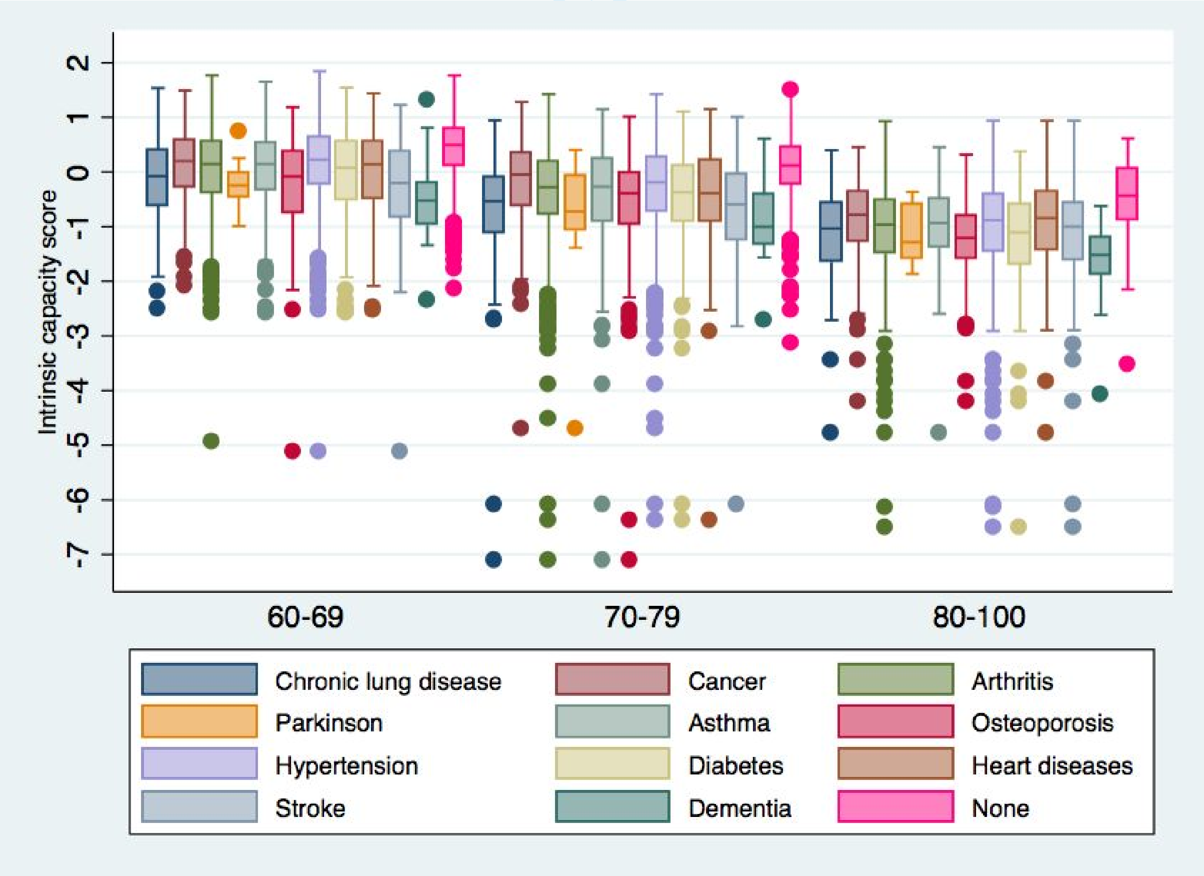

* Value 0 on the y-axis represents the mean Intrinsic capacity score for entire population.

Pathways to care dependence

In the simple mediation model, we tested the direct effect of intrinsic capacity on the incident loss of ADLs and IADLs and the indirect effect through multimorbidity (Supplementary table 5s, Supplementary figure1s). Intrinsic capacity predicted the incident loss of ADLs and IADLs both directly and indirectly, even after controlling for age, sex, education, and wealth. In comparisons of the effect size, the direct effect of intrinsic capacity on IADL and IADL was much more prominent than the indirect mediational effect through multimorbidity. In terms of proportion, only a small proportion of the effect of intrinsic capacity on the incidence ADL (8.7%) and IADL (5.2%) occurred indirectly through multimorbidity. A bias-corrected bootstrap confidence interval for this direct and indirect effect, which was based on a 10,000-bootstrap sample, was entirely above zero, thus suggesting that these effects are statistically significant.

The results of serial multiple mediators modelling of the relationships between the incident loss of ADLs and IADLs and personal characteristics, intrinsic capacity scores and multimorbidity are shown in Figures 5 and 6.

Figure 5: Direct and indirect effect of characteristics on activities of daily living

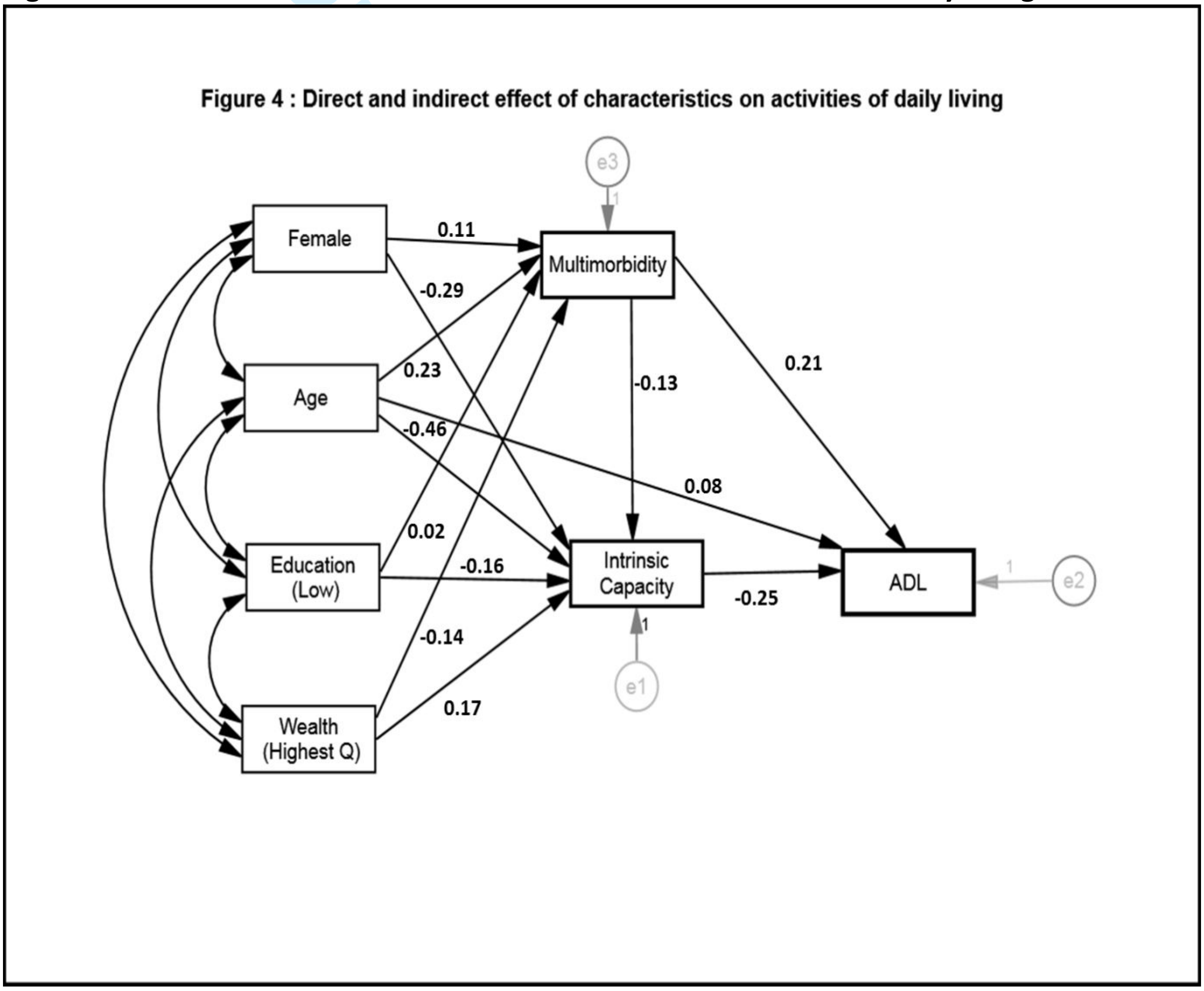

Model fit information: Chi-square =5.9 (df=3), RMSEA=0.02(90% CI 0.001 to 0.05), CFI=0.99, TLI=0.98 and SRMR (Standardized Root Mean Square Residual)=0.016. The paths in the figure are set out to test direct relationship between personal characteristics on ADL difficulties and indirect relationship through intrinsic capacity and multimorbidity.

Figure 6: Direct and indirect effect of characteristics on instrumental activities of daily living

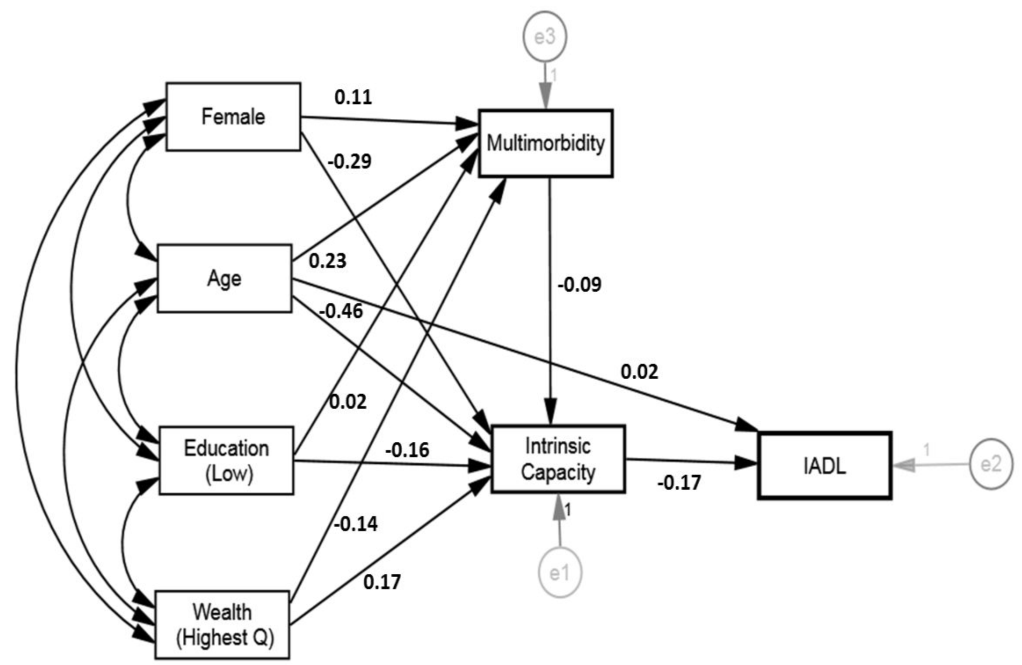

Figure 5 : Direct and indirect effect of characteristics on instrumental activities of daily

Model fit information: Chi-square =4.4 (df=4), RMSEA=0.008(90% CI 0.001 to 0.03), CFI=0.99 ,TLI=0.99 and SRMR (Standardized Root Mean Square Residual)=0.021. The paths in the figure are set out to test direct relationship between personal characteristics on IADL difficulties and indirect relationship through intrinsic capacity and multimorbidity.

Both intrinsic capacity score and multimorbidity independently predicted incident loss of ADLs, however only intrinsic capacity independently predicted incident loss of IADLs. Except age, none of the personal characteristics (sex, wealth and education) had a direct effect on incident loss of ADLs and IADLs (supplementary table 6s). Personal characteristics were strongly associated with both intrinsic capacity and multimorbidity, and the relationship between all personal characteristics (including chronological age) and the incident loss of ADLs and IADS operated through multimorbidity or intrinsic capacity. A greater proportion of the impact of age on outcomes (30% for ADLs and 39% for IADLs) occurred indirectly through intrinsic capacity than directly (24% for both ADLs and IADLs).

The specific indirect effect of all personal characteristics (age, sex, education, and wealth) on the incident loss of ADL and IADL through intrinsic capacity was statistically significant (Table 6s). None of the indirect effect of personal characteristics on incident loss of IADLs operating through multimorbidity was statistically significant. This implies that specific indirect effects of personal characteristics on IADL were mainly transmitted through intrinsic capacity rather than multimorbidity. Model fit information for all path analysis was provided in supplementary table 7s.

In a moderation analysis, after including the interaction term (age*intrinsic capacity), the direct effect of chronological age on incident IADL was not statistically significant (-0.03, pvalue = 0.16). The effect of chronological age on IADL was moderated by a person's level of intrinsic capacity (-0.526, pvalue=0.004), with the relationship between chronological age and IADL only being significant for people with low intrinsic capacity (figure 2s). Similarly, intrinsic capacity moderated the effect of chronological age on incident loss of ADL, after controlling for personal characteristics and multimorbidity (-0.472, pvalue =0.03).

DISCUSSION:

The WHO model of *Healthy Ageing* provides a transformative framework by which to consider health in older age. Rather than using the entry points of chronological age or disease, the model is built around the concept of intrinsic capacity - all the individual level characteristics that contribute to a person's ability to be and to do what they have reason to value. However, there has been little empirical analysis of the concept and a clear understanding of a possible structure for intrinsic capacity is lacking.

We used a large longitudinal study on ageing to explore the possible structure and predictive validity of the intrinsic capacity concept. We developed a total capacity score for each study participant and found it to be a powerful predictor of incident care dependence, even after accounting for chronological age and the presence, or number, of key health conditions. Factor analysis suggested a structure comprising 5 sub factors - psychological, sensory, cognitive, vitality and locomotor. This may provide a frame for the construct that is readily applicable to research and clinical practice.

These findings suggest that the intrinsic capacity concept has an empirical rigour and captures information beyond that generally considered in research or clinical practice. It also suggests that multiple domains of capacity can be aggregated into a meaningful overall measure of health status. If confirmed by future studies, these findings have a number of significant implications. For example, routine monitoring of intrinsic capacity might enable clinicians to flag when trajectories of capacity in the second half of life are veering off normal - a similar approach to the way child development charts currently guide paediatric practice[39]. A recent meeting of expert geriatricians convened by WHO confirmed that this would be useful, particularly if score changes could be interpreted in ways that have clinical relevance[40]. The factor structure of capacity identified in this analysis may provide a framework that achieves this by allowing clinicians to identify and address the drivers of any changes.

Measurable trajectories of capacity may also be useful as research outcomes of interest. As continuous measures that can be monitored at multiple time points, they allow a more nuanced and powerful analysis than approaches that use crude categorical measures of late life events such as mortality or incident loss of ADLs and IADLs[41]. Moreover, if information was available on trajectories of capacity across the full second half of life, this may facilitate the identification of mid-life influences on late life health which may be amenable to intervention. This is likely to become more feasible with the rapid development of wearable and communications devices which are already generating large amounts of relevant and routinely collected data. Appropriate algorithms could be developed to process this information to describe trajectories of capacity that could inform self-management, clinical practice and research.

Using trajectories of capacity as a research outcome may also allow better comparison between the impacts of interventions for different conditions. Furthermore, as medicine becomes increasingly personalised and precise, better information is needed on how different subpopulations may respond to specific interventions[42]. Stratifying by intrinsic capacity may provide a useful way of identifying the groups for which interventions are most effective and may be more appropriate than categorisation by chronological age or comorbidity.

One critical issue requiring further work is that not all five subfactors appear to operate at the same level. The cognitive, locomotor, sensory and psychological sub factors can be thought of as overt expressions of capacity.

For peer review only

On the other hand, dehydroepiandrosterone, IGF-1, haemoglobin and forced expiratory volume (included in the vitality sub factor) are elements of the biologic systems that underlie these overt manifestations of capacity[43]. Grip strength, the other characteristic loading to the vitality subfactor, can also be considered a marker of broader underlying factors such as nutritional, immune and hormonal status, and in this sense it is interesting that it loaded separately to locomotor capacity.[44 45]

The vitality sub factor interacts strongly with the other subfactors and part of the contribution it makes to the intrinsic capacity score is through the influence it has on these overt expressions of capacity. However it also loaded independently to the general factor (intrinsic capacity).

One possible conceptual frame for these relationships starts with a vitality domain describing variance in the complex and dynamic biologic systems which sustain life and functioning. When accumulated deficits in these systems reach a certain point they become manifest in the overt losses of capacity that are commonly associated with ageing. However, deficits in these systems that may not yet be expressed in overt manifestations are also likely have implications for the ability of the individual to retain their level of functioning. This residual is consistent with the notion of physiologic reserves or physiologic “resilience”. A total measure of vitality may thus capture an individual’s “biological age”.

Figure 6 shows how these domains might hypothetically relate. We have included a space for specific expressed capacities not captured in the four domains identified in our analysis (for example continence and speech). Within the vitality construct we have included cellular level characteristics as well as the contribution of higher physiologic systems. This is consistent with our analysis but also suggests at how characteristics not assessed (see strengths and weaknesses) might be considered in a conceptual frame for intrinsic capacity.

For peer review only

Figure 6: Conceptual frame for the construct of Intrinsic Capacity

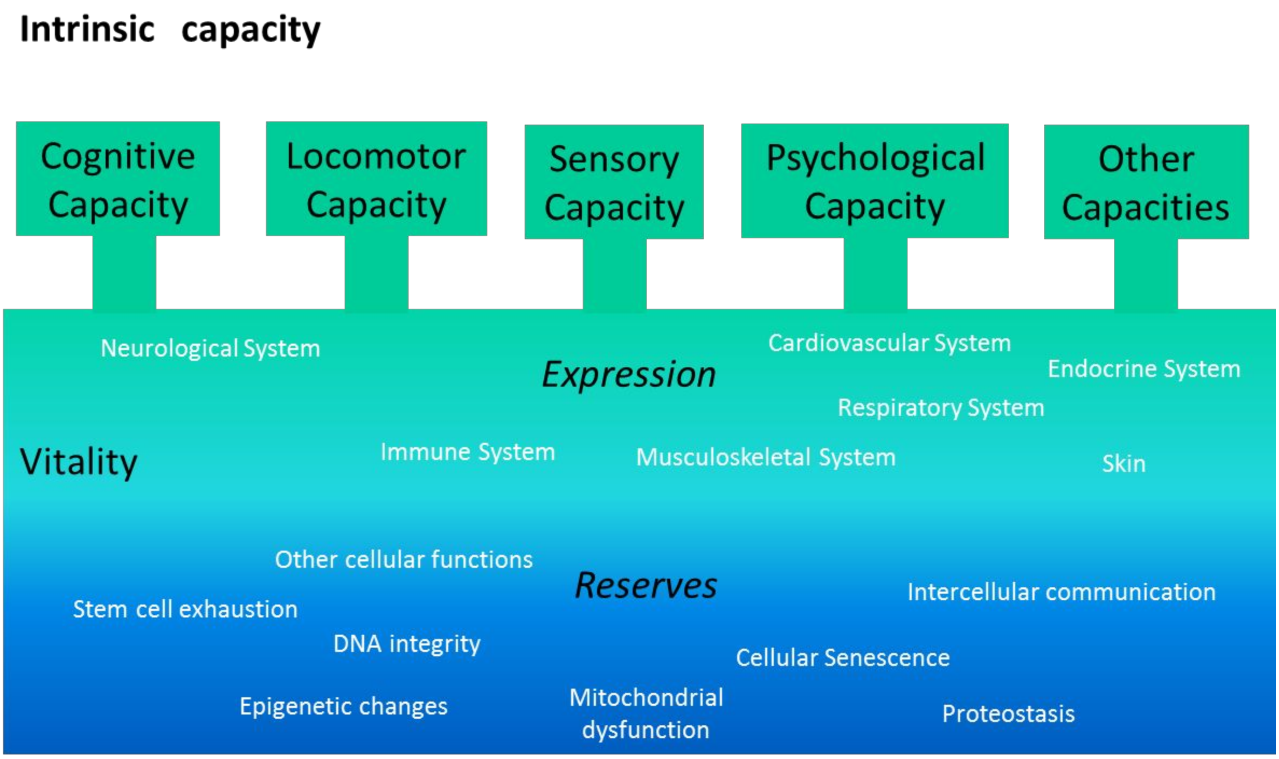

A second issue is that all the more overt capacities also interact (supplementary table 8s). This could be explained using the conceptual frame proposed above since the biologic drivers of these capacities are shared. This finding is also consistent with research and clinical experience which suggest that decrements in one domain of capacity may have clinically relevant impacts on other domains. For example, gait speed can be influenced by simultaneously drawing on an individual's cognitive capacity (e.g. by being asked to count backwards). These complex interactions may indeed provide the opportunity for "stress" testing of scores in any single domain[46].

However, the combined score we have calculated takes no account of thresholds that may exist within each subfactor. For example, cognitive capacity may fall to the point where it becomes impossible for an individual to survive without appropriate care and support, even though they may retain perfect capacity in each other domain and thus retain a relatively high total capacity score. This emphasises the need to assess the multiple dimensions of capacity to fully assess the clinical importance of changes in total score.

Strengths and limitations of study

A strength of this study is that it is a large, nationally representative sample of older people living in England with good follow-up. Unlike approaches that use a composite total score which assumes that each indicator or measure contributes equally to the general factor (i.e. intrinsic capacity), we used the bifactor model scores that represents a pure measure of the underlying latent trait of interest, after controlling for all five specific sub-factors [47]. Hence, a theoretically error-free score was used in all analysis to study the unique contribution of intrinsic capacity and its components in the prevention of care dependence.
Secondly, the longitudinal nature of the study allowed us to examine the direction of causality. Thirdly, most of the indicators of intrinsic capacity were measured using objective performance tests, limiting opportunities for response or interviewer bias.

However, it is important to note that the measures included in the ELSA study are neither complete nor random. They were chosen to inform specific research questions of interest to the investigators, rather than to create an overall measure of intrinsic capacity[12]. Nevertheless, since these questions largely draw on existing knowledge and research priorities, they cover aspects of most domains that might be conceptualised within the notion of capacity. Some potential components of capacity cannot be readily measured objectively (for example energy levels). Others require complex assessments that are beyond the scope of primary care or population-based research (for example, continence, cardiovascular capacity). Changes in other important attributes like the capacity for speech are important but less common. A number of key biomarkers, for example telomere length and immune function, were also missing from this dataset. Thus, while the set of indicators considered in this analysis can be considered relatively comprehensive, they are not complete in their ability to measure all aspects of capacity. Moreover, while we attempted to limit analysis to objective measures, the only data available on sensory and psychological capacities was through self report. This should not have had a significant impact on the construct of capacity, but may have had a marginal influence on the longitudinal analysis we undertook.

Despite carefully accounting for potential confounders, measurement error in their assessment, particularly the difference between participants who could and could not provide complete information on all exposure measures, may have biased associations. Also, the number of chronic diseases included in the analysis are limited, hence there is possibility of residual confounding.

Our findings are, however, consistent with previous research on the sub factors that were included in our analysis. Several longitudinal studies have shown strong predictive validity of cognitive (namely memory and executive function) [48 49], locomotor (gait or chair rise)[50-52], sensory (vision and hearing)[23 53-55], vitality (hand grip strength or FEV)[56-59], and psychological [60] indicators in relation to incident loss of ADL and IADL. Studies have also demonstrated associations between indicators of intrinsic capacity and survival. In particular, studies of locomotor and cognitive functions have shown that these indicators are predictors of premature mortality in community dwelling populations[61-63] . Yet, traditionally, these characteristics have often been considered independently. The intrinsic capacity concept provides a vehicle for assessing how they relate to each other and a possible approach to better quantify ambiguous notions such as "health" in older age into research and clinical practice[40 64].

Conclusions

Measurement of intrinsic capacity is feasible with commonly used measures and appears to provide useful predictive information on an individual's subsequent functioning. The proposed general factor and sub-factors structure may contribute to a transformative paradigm for future research and clinical practice.

For peer review only

Contributorship: JRB conceived of the research, oversaw analysis and was responsible for final drafting of the paper. JAT undertook all analyses, reviews of related literature, and contributed to drafting of the paper. MC contributed to conceptualisation, reviews of related literature and drafting of the paper. IAC contributed to conceptualisation, reviews of related literature and drafting of the paper. All authors reviewed and approved the final manuscript submitted for publication.

Funding: This research received no specific grant from any funding agency in the public, commercial or not-for-profit sectors.

Patient consent: The study uses secondary data from ELSA available publicly. All participants gave full informed written consent for participation in the study.

Ethical approval: Ethical approval for ELSA was obtained from NHS Research Ethics Committees under the National Research and Ethics Service (NRES), and participants gave full informed written consent for participation. More information on ELSA can be found at http://www.ifs.org.uk/elsa/documentation.php.

Declaration of interests: The authors received no support from any organisation for the submitted work; have no financial relationships with any organisations that might have an interest in the submitted work in the previous three years; and have no other relationships or activities that could appear to have influenced the submitted work.

Acknowledgments: The views expressed in this paper are those of the authors and do not necessarily reflect the views of WHO. The authors would like to thank the ELSA participants, the ELSA researchers and the UK Data Service for enabling the use of ELSA data for this analysis.

Data sharing statement: No additional data are available**.** However, ELSA dataset and information on all currently archived can be freely accssed through the UK Data Archive (https://www.elsa-project.ac.uk/availableData).

References :

1. WHO. World Report on Ageing and Health. In: John Beard AO, Andrew Cassels, ed. Geneva, Switzerland Department of Ageing and Life Course ,World Health Organization, 2015.
2. Beard JR, Officer A, de Carvalho IA, et al. The World report on ageing and health: a policy framework for healthy ageing. The Lancet 2016;**387**(10033):2145-54.
3. Guralnik JM, Ferrucci L. Assessing the building blocks of function: utilizing measures of functional limitation. Am J Prev Med 2003;**25**(3 Suppl 2):112-21.
4. Guralnik JM, Ferrucci L. Assessing the building blocks of function: Utilizing measures of functional limitation. American Journal of Preventive Medicine 2003;**25**(3, Supplement 2):112-21.
5. Ben-Shlomo Y, Cooper R, Kuh D. The last two decades of life course epidemiology, and its relevance for research on ageing. Int J Epidemiol 2016;**45**(4):973-88.

6. Belsky DW, Caspi A, Houts R, et al. Quantification of biological aging in young adults. Proceedings of the National Academy of Sciences 2015;**112**(30):E4104-E10.
7. Gershon RC, Wagster MV, Hendrie HC, et al. NIH Toolbox for Assessment of Neurological and Behavioral Function. Neurology 2013;**80**(11 Supplement 3):S2-S6.
8. Chatterji S, Byles J, Cutler D, et al. Health, functioning, and disability in older adults—present status and future implications. The Lancet 2015;**385**(9967):563-75.
9. Ferrucci L, Cooper R, Shardell M, et al. Age-Related Change in Mobility: Perspectives From Life Course Epidemiology and Geroscience. The Journals of Gerontology: Series A 2016;**71**(9):1184-94.
10. Milot E, Morissette-Thomas V, Li Q, et al. Trajectories of physiological dysregulation predicts mortality and health outcomes in a consistent manner across three populations. Mechanisms of Ageing and Development 2014;**141-142**:56-63.
11. Fieo RA, Austin EJ, Starr JM, et al. Calibrating ADL-IADL scales to improve measurement accuracy and to extend the disability construct into the preclinical range: a systematic review. BMC Geriatrics 2011;**11**(1):42.
12. Steptoe A, Breeze E, Banks J, et al. Cohort profile: the English longitudinal study of ageing. Int J Epidemiol 2013;**42**(6):1640-8.
13. Weber D. Differences in physical aging measured by walking speed: evidence from the English Longitudinal Study of Ageing. BMC Geriatr 2016;**16**:31.
14. Jones CJ, Rikli RE, Beam WC. A 30-s chair-stand test as a measure of lower body strength in community-residing older adults. Research quarterly for exercise and sport 1999;**70**(2):113-9.
15. Guralnik JM, Simonsick EM, Ferrucci L, et al. A short physical performance battery assessing lower extremity function: association with self-reported disability and prediction of mortality and nursing home admission. J Gerontol 1994;**49**(2):M85-94.
16. Dodds RM, Syddall HE, Cooper R, et al. Grip strength across the life course: normative data from twelve British studies. PLoS One 2014;**9**(12):e113637.
17. Yohannes AM, Tampubolon G. Changes in lung function in older people from the English Longitudinal Study of Ageing. Expert Rev Respir Med 2014;**8**(4):515-21.
18. Graig R, Deverill C, Pickering K. Quality control of blood, saliva and urine analytes. In: J; SKM, ed. Health survey for England 2004, Methodology and documentation. London: The Information Centre, 2006:34-41.
19. Souza-Teodoro LH, de Oliveira C, Walters K, et al. Higher serum dehydroepiandrosterone sulfate protects against the onset of depression in the elderly: Findings from the English Longitudinal Study of Aging (ELSA). Psychoneuroendocrinology 2016;**64**:40-6.
20. Grimaldi E, Scopacasa F. Evaluation of the Abbott CELL-DYN 4000 hematology analyzer. Am J Clin Pathol 2000;**113**(4):497-505.
21. Newcastle. FL. DPC Immulite 2000—IGF-1 (Laboratory summary of methods used up to 02 February 2012, available from laboratory on request). United Kingdom, 2012.
22. Liljas AEM, Carvalho LA, Papachristou E, et al. Self-reported vision impairment and incident prefrailty and frailty in English community-dwelling older adults: findings from a 4-year follow-up study. J Epidemiol Community Health 2017.
23. Liljas AEM, Carvalho LA, Papachristou E, et al. Self-Reported Hearing Impairment and Incident Frailty in English Community-Dwelling Older Adults: A 4-Year Follow-Up Study. J Am Geriatr Soc 2017;**65**(5):958-65.
24. Steel N, Huppert FA, McWilliams B, et al. Physical and cognitive function. In: J MMBJBRLCN, ed. Health, Wealth and Lifestyles of the Older Population in England: the 2002 English Longitudinal Study of Ageing. London: Institute of Fiscal Studies, 2003:249-300.

For peer review only

25. Shao Z, Janse E, Visser K, et al. What do verbal fluency tasks measure? Predictors of verbal fluency performance in older adults. Frontiers in Psychology 2014;**5**:772.
26. Batty GD, Deary IJ, Zaninotto P. Association of Cognitive Function With Cause-Specific Mortality in Middle and Older Age: Follow-up of Participants in the English Longitudinal Study of Ageing. American Journal of Epidemiology 2016;**183**(3):183-90.
27. Carleton RN, Thibodeau MA, Teale MJ, et al. The center for epidemiologic studies depression scale: a review with a theoretical and empirical examination of item content and factor structure. PLoS One 2013;**8**(3):e58067.
28. Jenkins CD, Stanton BA, Niemcryk SJ, et al. A scale for the estimation of sleep problems in clinical research. J Clin Epidemiol 1988;**41**(4):313-21.
29. Dhalwani NN, O'Donovan G, Zaccardi F, et al. Long terms trends of multimorbidity and association with physical activity in older English population. Int J Behav Nutr Phys Act 2016;**13**:8.
30. Chan KS, Kasper JD, Brandt J, et al. Measurement equivalence in ADL and IADL difficulty across international surveys of aging: findings from the HRS, SHARE, and ELSA. J Gerontol B Psychol Sci Soc Sci 2012;**67**(1):121-32.
31. M*plus* [program]. 8 version. Los Angeles: Muthén & Muthén, 2017.
32. Stata Statistical Software [program]. 14 version. TX: StataCorp LLC 2015.
33. Hooper DC, J ; Mullen, M. Structural Equation Modeling: Guidelines for Determining Model Fit. Electronic Journal on Business Research Methods 2008;**6**(1):53-60.
34. Schweizer. K, DiStefano. C. *Principles and Methods of Test Construction: Standards and Recent Advances*: Hogrefe Publishing, 2016.
35. Hayes. AF. *Introduction to Mediation, Moderation and Conditional Process Analysis: A Regression Based Approach*. New York: The Guilford Press, 2013.
36. Preacher KJ, Kelley K. Effect size measures for mediation models: quantitative strategies for communicating indirect effects. Psychol Methods 2011;**16**(2):93-115.
37. Miocevic M, O'Rourke HP, MacKinnon DP, et al. Statistical properties of four effect-size measures for mediation models. Behavior research methods 2017.
38. J.; JPN. Tests of certain linear hypotheses and their applications to some educational problems. Statistical Research Memoirs 1936(1):57-93.
39. de Onis M, Onyango A, Borghi E, et al. Worldwide implementation of the WHO Child Growth Standards. Public Health Nutr 2012;**15**(9):1603-10.
40. WHO. Clinical Consortium on Healthy Ageing. Secondary Clinical Consortium on Healthy Ageing 2016. http://www.who.int/ageing/health-systems/clinical-consortium-meeting/en/.
41. Mlinac ME, Feng MC. Assessment of Activities of Daily Living, Self-Care, and Independence. Archives of Clinical Neuropsychology 2016;**31**(6):506-16.
42. Morley JE, Vellas B. Patient-Centered (P4) Medicine and the Older Person. J Am Med Dir Assoc 2017;**18**(6):455-59.
43. Belsky DW, Caspi A, Houts R, et al. Quantification of biological aging in young adults. Proceedings of the National Academy of Sciences of the United States of America 2015;**112**(30):E4104-10.
44. Norman K, Stobäus N, Gonzalez MC, et al. Hand grip strength: Outcome predictor and marker of nutritional status. Clinical Nutrition 2011;**30**(2):135-42.
45. Visser M, Deeg DJH, Lips P. Low Vitamin D and High Parathyroid Hormone Levels as Determinants of Loss of Muscle Strength and Muscle Mass (Sarcopenia): The Longitudinal Aging Study Amsterdam. The Journal of Clinical Endocrinology & Metabolism 2003;**88**(12):5766-72.

For peer review only

46. Montero-Odasso MM, Sarquis-Adamson Y, Speechley M, et al. Association of Dual-Task Gait With Incident Dementia in Mild Cognitive Impairment: Results From the Gait and Brain Study. JAMA Neurol 2017;**74**(7):857-65.
47. DeMars CE. A Tutorial on Interpreting Bifactor Model Scores. International Journal of Testing 2013;**13**(4):354-78.
48. Johansson MM, Marcusson J, Wressle E. Cognitive impairment and its consequences in everyday life: experiences of people with mild cognitive impairment or mild dementia and their relatives. Int Psychogeriatr 2015;**27**(6):949-58.
49. Dodge HH, Kadowaki T, Hayakawa T, et al. Cognitive impairment as a strong predictor of incident disability in specific ADL-IADL tasks among community-dwelling elders: the Azuchi Study. Gerontologist 2005;**45**(2):222-30.
50. Vermeulen J, Neyens JC, van Rossum E, et al. Predicting ADL disability in community-dwelling elderly people using physical frailty indicators: a systematic review. BMC Geriatr 2011;**11**:33.
51. Ostir GV, Markides KS, Black SA, et al. Lower body functioning as a predictor of subsequent disability among older Mexican Americans. J Gerontol A Biol Sci Med Sci 1998;**53**(6):M491-5.
52. Gill TM, Williams CS, Tinetti ME. Assessing risk for the onset of functional dependence among older adults: the role of physical performance. J Am Geriatr Soc 1995;**43**(6):603-9.
53. Cigolle CT, Langa KM, Kabeto MU, et al. Geriatric conditions and disability: the Health and Retirement Study. Ann Intern Med 2007;**147**(3):156-64.
54. Crews JE, Campbell VA. Vision impairment and hearing loss among community-dwelling older Americans: implications for health and functioning. American journal of public health 2004;**94**(5):823-9.
55. Lin MY, Gutierrez PR, Stone KL, et al. Vision impairment and combined vision and hearing impairment predict cognitive and functional decline in older women. J Am Geriatr Soc 2004;**52**(12):1996-2002.
56. Taekema DG, Gussekloo J, Maier AB, et al. Handgrip strength as a predictor of functional, psychological and social health. A prospective population-based study among the oldest old. Age and ageing 2010;**39**(3):331-7.
57. Al Snih S, Markides KS, Ottenbacher KJ, et al. Hand grip strength and incident ADL disability in elderly Mexican Americans over a seven-year period. Aging Clin Exp Res 2004;**16**(6):481-6.
58. Hegendorfer E, Vaes B, Mathei C, et al. Prognostic value of short-term decline of forced expiratory volume in 1 s over height cubed (FEV1/Ht3) in a cohort of adults aged 80 and over. Aging Clin Exp Res 2017.
59. Abe T ST, Yoshida H,. The relationship between pulmonary function and physical function and mobility in community-dwelling elderly women aged 75 years or older J Phys Ther Sci 2011;**23**:443-49.
60. Penninx BW, Guralnik JM, Ferrucci L, et al. Depressive symptoms and physical decline in community-dwelling older persons. JAMA 1998;**279**(21):1720-6.
61. Amuthavalli Thiyagarajan J, Bryce R, Prina M, et al. Frailty and the prediction of dependence and mortality in low- and middle-income countries: a 10/66 population-based cohort study. BMC Med 2015;**13**:138.
62. Cooper R, Kuh D, Hardy R, et al. Objectively measured physical capability levels and mortality: systematic review and meta-analysis. BMJ 2010;**341**.
63. Studenski S, Perera S, Patel K, et al. Gait speed and survival in older adults. JAMA 2011;**305**(1):50-58.

For peer review only

64. Cesari M, Araujo de Carvalho I, Amuthavalli Thiyagarajan J, et al. Evidence for The Domains Supporting The Construct of Intrinsic Capacity. The Journals of Gerontology: Series A 2018:gly011-gly11.

For peer review only

Supplementary analysis

Table 1s : Sample characteristics

**Demographic and health characteristics**	**Wave 4**
**Age , mean (SD)**	70.5 (7.9)
**Sex**	
Male	2535(45.9%)
Female	2981(54.0%)
**Education**	
No education	1712(31.2%)
Intermediate	2090(38.1%)
Higher education	1683(30.6%)
**Wealth quintile**	
1 (lowest)	868 (16.3%)
2	959(18.1%)
3	1095 (20.6%)
4	1172(22.1%)
5 (highest)	1202(22.7%)
**Multimorbidity**	
0	1241(22.5%)
1 or 2	3073(55.9%)
3 or more	1182(21.5%)
**Activities of daily living**	
No ADL limitation	4410(79.9%)
1 or 2 limitations	880(15.9%)

For peer review only

3 or more limitations	226 (4.1%)
**Instrumental activities of daily living**	
No limitation	4292(77.8%)
1 or 2 limitations	990 (17.9%)
3 or more limitations	234 (4.2%)

Table 2s : Model fit statistics of ESEM and CFA models

**Models**	**Fit statistics**				
	**χ2**	**df**	**CFI**	**TLI**	**REMSEA (90% CI)**
**ESEM**					
One-factor	5385.6	104	0.63	0.57	0.136 (0.133 -0.139)
Two-factors	2570.8	89	0.82	0.76	0.101(0.098- 0.104)
Three-factors	927.5	75	0.94	0.90	0.064(0.061-0.068)
Four- factors	277.4	62	0.98	0.97	0.036(0.031-0.040)
Five-factors	117.9	50	0.99	0.98	0.022(0.017-0.030)
Six-factors (one general factor and five sub-factors) [1]	71.2	39	0.99	0.99	0.012(0.011-0.024)
**CFA**					
One-factor	6735.9	104	0.56	0.49	0.154(0.150-0.150)
Second-order	2369.9	102	0.94	0.92	0.073(0.070-0.080)
Correlated five factors	1782.3	103	0.95	0.92	0.060(0.050-0.060)
Bi-factor (one general factor and five sub-factors)	1180.6	89	0.98	0.97	0.035(0.033 -0.037)

[1] *Bi-factor exploratory analysis is conducted in SEM framework.*

For peer review only

Table 3s: Intrinsic capacity Bi-factor Exploratory Factor Analysis with standardized factor loadings

S.no	Name of the indicator	General factor (Intrinsic capacity)	Factor 1 (Sensory)	Factor 2 (Cognitive)	Factor 3 (Psychological)	Factor 4 (Locomotor)	Factor 5 (Vitality)
1.	Near-vision	**0.31**	**0.80**	-0.01	0.06	0.002	-0.02
2.	Distance-vision	**0.31**	**0.84**	-0.01	0.02	0.01	-0.005
3.	Hearing	**0.22**	**0.34**	0.02	0.05	0.05	0.03
4.	Delayed recall	**0.49**	-0.01	**0.43**	-0.08	-0.01	-0.04
5.	Verbal fluency	**0.56**	-0.04	**0.36**	-0.06	-0.03	-0.04
6.	Letter cancellation	**0.33**	0.07	**0.38**	-0.07	0.04	0.10
7.	Hand-grip strength	**0.69**	-0.03	-0.06	0.02	-0.04	**0.32**
8.	Forced expiratory volume	**0.58**	-0.02	-0.03	-0.03	-0.03	**0.30**
9.	IGF-1	**0.27**	-0.01	0.03	0.08	0.06	**0.31**
10.	DHEAS	**0.37**	-0.03	-0.18	0.03	-0.02	**0.30**
11.	Hemoglobin	**0.42**	-0.04	-0.21	0.06	0.004	**0.31**
12.	Gait-speed	**0.52**	0.02	-0.06	0.03	**0.65**	0.002
13.	Chair-rise	**0.42**	0.03	0.03	0.01	**0.35**	-0.01
14.	Balance	**0.47**	0.01	0.02	-0.06	**0.21**	-0.02
15.	Affect	**0.33**	0.09	-0.01	**0.40**	0.15	-0.07
16.	Sleep	**0.28**	0.07	-0.06	**0.94**	0.00	0.01

Table 4s: Construct validity of intrinsic capacity and sub-domain score

Demographic and health characteristics	Intrinsic capacity Regression coefficient (95% CI)	Sensory Regression coefficient (95% CI)	Cognitive Regression coefficient (95% CI)	Vitality Regression coefficient (95% CI)	Psychological Regression coefficient (95% CI)	Locomotor Regression coefficient (95% CI)
**Age**	-0.052 (-0.054 to -0.046)***	-0.002(-0.008 to 0.003)	-0.02(-0.023 to -0.019)***	-0.021(-0.024 to -0.019)***	-0.003(-0.005 to -0.0003)*	-0.009(-0.011 to -0.007)***
**Sex**						
Male	Ref 1	Ref 1	Ref 1	Ref 1	Ref 1	Ref 1
Female	-0.322 (-0.358 to -0.286)***	0.036(-0.037 to 0.110)	0.27(0.240 to 0.302)***	-0.881(-0.905 to -0.857)***	-0.251 (-0.314 to -0.189)***	0.099(0.059 to 0.139)***
**Education**						
No education	Ref1	Ref 1	Ref 1	Ref 1	Ref 1	Ref 1
Intermediate	0.462(0.420 to 0.504)***	0.085(0.037 to 0.133)***	0.267(0.230 to 0.304)***	0.068(0.029 to 0.106)***	0.027(-0-0.017 to 0.073)	0.058(0.028 to 0.094)***
Higher education	0.779(0.735 to 0.823)***	0.114(0.064 to 0.164)***	0.4042)***	0.239 (0.198 to 0.279)**3	-0.015(-0.062 to 0.032)	0.063(0.031 to 0.094)***
**Wealth quintile**						

1 (lowest)	Ref 1	Ref 1	Ref 1	Ref 1	Ref 1	Ref 1
2	-0.020(-0.085 to 0.044)	0.045(-0.025 to 0.116)	-0.072(-0.128 to -0.169)*	-0.072 (-0.132 to -0.129)*	-0.045(-0.113 to 0.230)	0.049(0.003 to 0.095)**
3	0.268(0.204 to 0.332)***	0.006(-0.150 to 0.163)	0.050 (-0.005 to 0.105)	0.016(-0.042 to 0.075)	-0.017(-0.152 to 0.116)	0.125(0.080 to 0.171)***
4	0.448(0.384 to 0.512)***	0.016(-0.141 to 0.164)	0.111(0.056 to 0.166)***	0.073 (0.014 to 0.131)*	-0.0530 (-0.119 to 0.0139)	0.155(0.110 to 0.200)***
5 (highest)	0.616(0.553 to 0.678)***	0.01(-0.142 to 0.159)	0.201 (0.147 to 0.255)***	0.099(0.041 to 0.156)**	0.055 (-0.074 to 0.184)	0.181(0.137 to 0.226)***
**Multimorbidity**						
0	Ref 1	Ref 1	Ref 1	Ref 1	Ref 1	Ref 1
1 or 2	-0.0237 (-0.279 to -0.195)***	-0.068(-0.151 to 0.014)	-0.012(-0.049 to 0.025)	-0.122(-0.160 to -0.085)***	0.035(-0.010 to 0.077)	-0.032(-0.061 to -0.003)*
3 or more	-0.764(-0.816 to -0.712)***	-0.057 (-0.151 to 0.014)	-0.067(-0.114 to -0.021)**	-0.308 (-0.354 to -0.261)***	-0.242(-0.350 to -0.133)***	-0.221(-0.251 to -0.185)***
**Activities of daily living**						
No ADL limitation	Ref 1	Ref 1	Ref 1	Ref 1	Ref 1	Ref 1
1 or 2 limitations	--0.471(-0.510 to -	-0.105(-0.147 to	-0.049(-0.082 to -	-0.096 (-0.131 to -	-0.134 (-0.187 to -	-0.097(-0.122 to -

For peer review only

	0.432)***	0.063)***	0.016)**	0.061)***	0.082)***	0.072)***
3 or more limitations	-0.857(-0.918 to -0.797)***	-0.116(-0.182 to -0.050)***	-0.088(-0.082 to -0.016)**	-0.072(-0.127 to -0.018)***	-0.028(-0.357 to 0.299)	-0.317 (-0.356 to -0.279)***
**Instrumental activities of daily living**						
No limitation	Ref 1	Ref 1	Ref 1	Ref 1	Ref 1	Ref 1
1 or 2 limitations	-0.636(-0.677 to -0.596)***	-0.097(-0.142 to -0.053)***	-0.077(-0.111 to -0.042)***	-0.127(-0.164 to -0.091)***	-0.105(-0.253 to 0.043)	-0.147 (-0.173 to -0.121)***
3 or more limitations	-1.067(-1.144 to -0.990)***	-0.108(-0.193 to -0.023)**	-0.396 (-0.462 to -0.330)***	-0.107(-0.177 to -0.036)**	0.273(-0.108 to 0.656)	-0.259 (-0.309 to -0.209)***

*** p value < 0.001, **p value <0.05.

Table 5s: Regression coefficient of direct effect of intrinsic capacity on ADL and IADL and indirect effect through multimorbidity

Causal path	ADL		IADL	
	Standardized Coefficient (SE)1	P value	Standardized Coefficient (SE)1	P value
**Total effect**				
Intrinsic capacity	**-0.52 (0.01)**	**<0.001**	**-0.48(0.02)**	**<0.001**
**Direct effect**				
Intrinsic capacity	**-0.40(0.03)**	**<0.001**	**-0.39(0.02)**	**<0.001**
**Indirect effect**				
Intrinsic capacity→Multimorbidity	**-0.039(0.005)**	**<0.001**	**-0.024(0.005)**	**<0.001**
	**R2=0.20, pvalue=<0.001**		**R2= 0.21, pvalue=<0.001**	

[1]Controlled for age, sex, education, and wealth

For peer review only

Table 6s: Direct of and indirect effect personal characteristics on ADL and IADL in serial multiple mediators (intrinsic capacity and multimorbidity)

	ADL		IADL	
**Causal path**	**Standardized Coefficient (SE)**	**P value**	**Standardized Coefficient (SE)**	**P value**
**Direct effect**				
Age	**0.006(0.02)**	**0.011**	**0.110 (0.03)**	**0.001**
Sex	-0.049(0.03)	0.061	-0.049(0.02)	0.051
Education	0.009(0.01)	0.571	-0.012(0.02)	0.613
Wealth	-0.013(0.01)	0.185	0.016(0.24)	0.510
Multimorbidity	0.036(0.01)	0.001	0.020(0.02)	0.402
Intrinsic capacity	-0.099(0.02)	<0.000	-0.142(0.02)	<0.001
**Specific indirect effect**				
Age → Intrinsic capacity	**0.053(0.01)**	**<0.001**	**0.050(0.01)**	**<0.001**
Age → multimorbidity	0.011(0.04)	0.004	0.003(0.01)	0.405
Female → intrinsic capacity	**0.047(0.010)**	**<0.001**	**0.048(0.01)**	**<0.001**
Female → multimorbidity	0.008(0.003)	0.008	0.002(0.003)	0.409
Education→intrinsic capacity	**-0.022(0.01)**	**<0.001**	**-0.02(0.01)**	**0.001**
Education→multimorbidity	0.000(0.002)	0.810	0.00(0.00)	0.817
Wealth →intrinsic capacity	**-0.021(0.01)**	**<0.001**	**-0.021 (0.01)**	**<0.001**
Wealth→multimorbidity	-0.009(0.01)	0.007	-0.002(0.001)	0.408
**Indirect effect**				
Age → Multimorbidity → Intrinsic capacity	0.002(0.001)	0.002	0.002(0.001)	0.002
Female → Multimorbidity → Intrinsic capacity	0.002(0.001)	0.004	0.002(0.001)	0.004
Education→ Multimorbidity → Intrinsic capacity	0.000(0.00)	0.810	0.000(0.00)	0.810
Wealth → Multimorbidity → Intrinsic capacity	-0.002(0.001)	0.003	-0.002(0.001)	0.003

For peer review only

For peer review only

Table 7s: Model fit information for Parallel and Sequential models examining pathways to ADL and IADL

**Models**	**Fit statistics**					**R2**		
**Activities of daily livings**	χ2	df	CFI	TLI	REMSEA (90% CI)	**Multimorbidity**	**Intrinsic capacity**	**ADL**
Model 1a : Indirect effect of age (and other covariates) on ADL is either mediated by multimorbidity or intrinsic capacity	43.9	5	0.96	0.89	0.06(0.04-0.08)	7.6%	42.6%	18.0%
Model 1b : Indirect effect of age(other covariates) on ADL is mediated by multimorbidity and intrinsic capacity	16.3	4	0.98	0.96	0.04(0.022 - 0.062)	7.6%	45.5%	18.9%
Model 1c : Effect of age direct and indirectly (other covariates) on ADL is mediated by multimorbidity and intrinsic capacity	5.92	3	0.99	0.98	0.02(0.001-0.05)	5.0%	39.1%	19.0%
**Instrumental Activities of Daily Livings**								
Model 2a: Effect of age (and other covariates) on IADL is either mediated by multimorbidity or intrinsic capacity	55.6	5	0.95	0.86	0.07(0.05-0.09)	7.8%	42.7%	35.4%
Model 2b : Indirect effect of age(and other covariates) on IADL is mediated by multimorbidity and intrinsic capacity	30.9	4	0.97	0.91	0.05(0.04-0.08)	7.9%	45.4%	31.2%
Model 2c : Effect of age direct and indirectly (other covariates) on IADL is mediated by multimorbidity and intrinsic capacity	4.4	4	0.99	0.99	0.008(0.001 – 0.03)	5.2%	39.1%	32.2%

For peer review only

Table 8s: Correlation between factors of intrinsic capacity

Domains	Cognitive	Sensory	Locomotor	Vitality	Psychological
**Cognitive**	1.000				
**Sensory**	0.283	1.000			
**Locomotor**	0.762	0.377	1.000		
**Vitality**	0.313	0.179	0.701	1.000	
**Psychological**	0.095	0.229	0.441	0.269	1.000

*all factors are significantly correlated with each (p>0.001)

For peer review only

Figure 1s: A statistical diagram of a simple mediation model (Direct and indirect) effect of intrinsic capacity on ADL and IADL

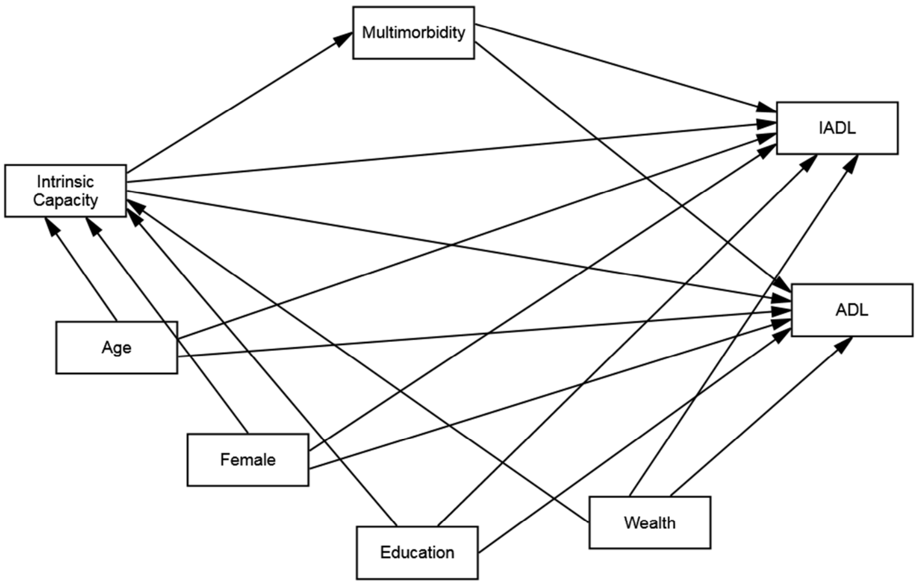

Figure 2s : Interaction effect of age and intrinsic capacity on incidence IADL.

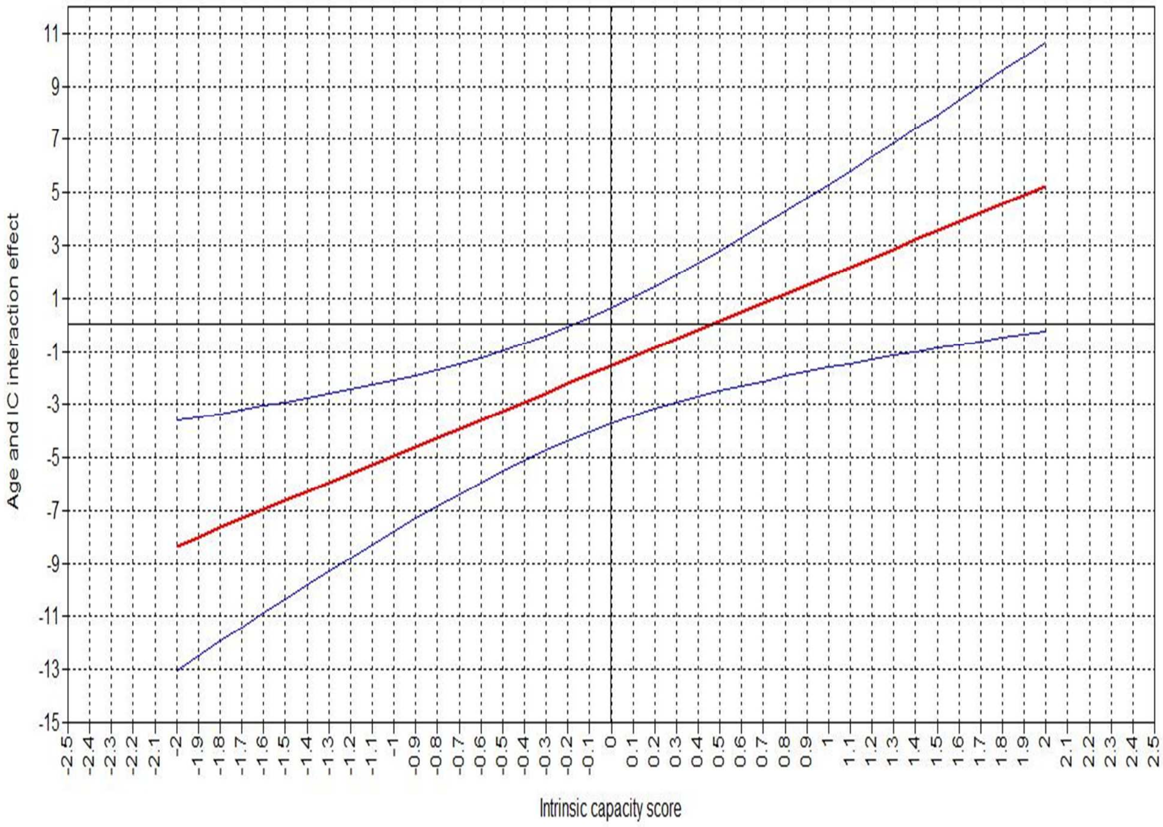

STROBE Statement—checklist of items that should be included in reports of observational studies

	Item No	Recommendation
**Title and abstract**	1	(*a*) Indicate the study's design with a commonly used term in the title or the abstract
		(*b*) Provide in the abstract an informative and balanced summary of what was done and what was found
**Introduction**		
Background/rationale	2	Explain the scientific background and rationale for the investigation being reported
Objectives	3	State specific objectives, including any prespecified hypotheses
**Methods**		
Study design	4	Present key elements of study design early in the paper
Setting	5	Describe the setting, locations, and relevant dates, including periods of recruitment, exposure, follow-up, and data collection
Participants	6	(*a*) *Cohort study*—Give the eligibility criteria, and the sources and methods of selection of participants. Describe methods of follow-up<br>*Case-control study*—Give the eligibility criteria, and the sources and methods of case ascertainment and control selection. Give the rationale for the choice of cases and controls<br>*Cross-sectional study*—Give the eligibility criteria, and the sources and methods of selection of participants
		(*b*) *Cohort study*—For matched studies, give matching criteria and number of exposed and unexposed<br>*Case-control study*—For matched studies, give matching criteria and the number of controls per case
Variables	7	Clearly define all outcomes, exposures, predictors, potential confounders, and effect modifiers. Give diagnostic criteria, if applicable
Data sources/ measurement	8*	For each variable of interest, give sources of data and details of methods of assessment (measurement). Describe comparability of assessment methods if there is more than one group
Bias	9	Describe any efforts to address potential sources of bias
Study size	10	Explain how the study size was arrived at
Quantitative variables	11	Explain how quantitative variables were handled in the analyses. If applicable, describe which groupings were chosen and why
Statistical methods	12	(*a*) Describe all statistical methods, including those used to control for confounding
		(*b*) Describe any methods used to examine subgroups and interactions
		(*c*) Explain how missing data were addressed
		(*d*) *Cohort study*—If applicable, explain how loss to follow-up was addressed<br>*Case-control study*—If applicable, explain how matching of cases and controls was addressed<br>*Cross-sectional study*—If applicable, describe analytical methods taking account of sampling strategy
		(*e*) Describe any sensitivity analyses

Continued on next page

Results		
Participants	13*	(a) Report numbers of individuals at each stage of study—eg numbers potentially eligible, examined for eligibility, confirmed eligible, included in the study, completing follow-up, and analysed
		(b) Give reasons for non-participation at each stage
		(c) Consider use of a flow diagram
Descriptive data	14*	(a) Give characteristics of study participants (eg demographic, clinical, social) and information on exposures and potential confounders
		(b) Indicate number of participants with missing data for each variable of interest
		(c) *Cohort study*—Summarise follow-up time (eg, average and total amount)
Outcome data	15*	*Cohort study*—Report numbers of outcome events or summary measures over time
		Case-control study—Report numbers in each exposure category, or summary measures of exposure
		Cross-sectional study—Report numbers of outcome events or summary measures
Main results	16	(*a*) Give unadjusted estimates and, if applicable, confounder-adjusted estimates and their precision (eg, 95% confidence interval). Make clear which confounders were adjusted for and why they were included
		(*b*) Report category boundaries when continuous variables were categorized
		(*c*) If relevant, consider translating estimates of relative risk into absolute risk for a meaningful time period
Other analyses	17	Report other analyses done—eg analyses of subgroups and interactions, and sensitivity analyses
**Discussion**		
Key results	18	Summarise key results with reference to study objectives
Limitations	19	Discuss limitations of the study, taking into account sources of potential bias or imprecision. Discuss both direction and magnitude of any potential bias
Interpretation	20	Give a cautious overall interpretation of results considering objectives, limitations, multiplicity of analyses, results from similar studies, and other relevant evidence
Generalisability	21	Discuss the generalisability (external validity) of the study results
**Other information**		
Funding	22	Give the source of funding and the role of the funders for the present study and, if applicable, for the original study on which the present article is based

*Give information separately for cases and controls in case-control studies and, if applicable, for exposed and unexposed groups in cohort and cross-sectional studies.

Note: An Explanation and Elaboration article discusses each checklist item and gives methodological background and published examples of transparent reporting. The STROBE checklist is best used in conjunction with this article (freely available on the Web sites of PLoS Medicine at http://www.plosmedicine.org/, Annals of Internal Medicine at http://www.annals.org/, and Epidemiology at http://www.epidem.com/). Information on the STROBE Initiative is available at www.strobe-statement.org.

BMJ Open

THE STRUCTURE AND PREDICTIVE VALUE OF INTRINSIC CAPACITY IN A LONGITUDINAL STUDY OF AGEING

Journal:	*BMJ Open*
Manuscript ID	bmjopen-2018-026119.R1
Article Type:	Research
Date Submitted by the Author:	12-Apr-2019
Complete List of Authors:	Beard, John; Organisation mondiale de la Sante, Ageing and Life Course<br>Jotheeswaran, AT; Organisation mondiale de la Sante, Department of Ageing and Life Course<br>Cesari, Matteo; Fondazione IRCCS Ca' Granda Ospedale Maggiore Policlinico, Università di Milano<br>Araujo de Carvalho, Islene; Organisation mondiale de la Sante
**Primary Subject Heading**:	Epidemiology
Secondary Subject Heading:	Geriatric medicine, Public health, Research methods
Keywords:	EPIDEMIOLOGY, GERIATRIC MEDICINE, PUBLIC HEALTH, PRIMARY CARE, GENERAL MEDICINE (see Internal Medicine)

SCHOLARONE™
Manuscripts

Article type : Original research paper

Title : The structure and predictive value of intrinsic capacity in a longitudinal study of ageing

John Beard[1],Jotheeswaran Amuthavalli Thiyagarajan[1], Matteo Cesari[2], Islene Araujo de Carvalho[1]

[1]Department of Ageing and Life Course, World Health Organization, Geneva, Switzerland
[2]Fondazione IRCCS Ca' Granda Ospedale Maggiore Policlinico, Università di Milano, Milano, Italy.

Corresponding author: John Beard, Department of Ageing and Life Course, World Health Organization, Geneva, Switzerland. Email ID: drjohnbeard@outlook.com.com

ABSTRACT:

Objectives: To assess the validity of the World Health Organization concept of intrinsic capacity in a longitudinal study of ageing; to identify whether this overall measure disaggregated into biologically plausible and clinically useful subdomains; and to assess whether total capacity predicted subsequent care dependence.

Design: Structural equation modeling of biomarkers and self-reported measures in the English Longitudinal Study of Ageing including exploratory factor analysis, exploratory bi-factor analysis and confirmatory factor analysis. Longitudinal mediation and moderation analysis of incident care dependence.

Settings: Community, United Kingdom

Participants: 2560 eligible participants aged over 60 years

Main outcome measures: Activities of daily living (ADL) and instrumental activities of daily living (IADL).

Results: One general factor (intrinsic capacity) and five sub-factors emerged: locomotor, cognitive; psychological; sensory; and "vitality". This structure is consistent with biological theory and the model had a good fit for the data (chi-square = 71.2 (df = 39)). The summary score of intrinsic capacity and specific sub-factors showed good construct validity. In a causal path model examining incident loss of ADL and IADL, intrinsic capacity had a direct relationship with the outcome - *RMSEA=0.02(90% CI 0.001 to 0.05) and RMSEA=0.008(90% CI 0.001 to 0.03) respectively* -and was a strong mediator for the effect of age, sex, wealth and education. Multimorbidity had an independent direct relationship with incident loss of ADLs but not IADLs, and also operated through intrinsic capacity. More of the indirect effect of personal characteristics on incident loss of ADLs and IADLs was mediated by intrinsic capacity than multimorbidity.

Conclusions: The WHO construct of intrinsic capacity appears to provide valuable predictive information on an individual's subsequent functioning, even after accounting for the number of multimorbidities. The proposed general factor and sub-domain structure may contribute to a transformative paradigm for future research and clinical practice.

For peer review only

Strength and limitations of this study

1. To our knowledge this is the first large population-based longitudinal analysis to examine the structure and predictive validity of the WHO concept of intrinsic capacity. We applied a rigorous psychometric approach for constructing a valid measurement model using commonly measured biomarkers and self-reported measures, allowing us to create a theoretically error-free composite score for intrinsic capacity, which was used in all analysis.
2. We used longitudinal data to minimize the potential for reverse causality and adjusted for multimorbidity to minimise confounding-by-disease; however, the potential of residual confounding cannot be completely eliminated.
3. This study shows that many of the commonly used assessments of health and functioning in older age have common variance (i.e. they are possibly measuring one underlying trait of an individual's health status) that is consistent with the WHO concept of intrinsic capacity.
4. This composite measure was structured in a way that is consistent with biological theory.
5. However, it is important to note that the measures included in the ELSA study are neither complete nor random. They were chosen to inform specific research questions of interest to the investigators, rather than to create an overall measure of intrinsic capacity. The consideration of other variables might influence both the overall score and the sub-factor structure.

For peer review only

INTRODUCTION

In 2015, the World Health Organization released the *World report on ageing and health*, which proposed a public health framework for action on population ageing[1 2]. Central to the Report is a new conceptual model for "*Healthy Ageing*". Rather than considering healthy ageing from the perspective of the presence or absence of disease, this functioning-based approach is oriented around building and maintaining the ability of older people to be, and to do, the things they have reason to value. The Report proposes that this "functional ability" is determined by the "intrinsic capacity" of the individual, the environments in which they live and the interaction between the individual and these environments. However, while the Report considers intrinsic capacity to be "all the physical and mental capacities" that an individual can draw on at any point in time, it does not provide a detailed description of the components of capacity, how they might be structured or how capacity and its components may be measured and monitored.

This reframing of the concept of healthy ageing builds on a growing body of research exploring patterns and determinants of functional status in older people. Many of these studies examine functioning in areas such as physical performance or cognition[3 4], and increasingly they are applying a life course perspective.[5] At the same time there is growing interest in the biological underpinnings of ageing and in identifying ways to measure "biological" age as distinct from chronological age.[6] This work all serves to better capture the heterogeneity that is a hallmark of ageing and helps researchers and clinicians advance from stereotypical notions of older age, and towards more personalised interventions to foster healthy ageing.

There has also been significant work identifying measures that might assess different domains of functioning at different stages in life.[7] However, there is less research and less agreement on how functional approaches for specific domains might together reflect the *overall* health status of older individuals.[8 9] It also remains unclear how specific functional domains such as locomotor and cognitive capacity relate to each other, and how the deficits in the complex and dynamic biological systems that underpin ageing relate to these more overt expressions of an individual's capacity.[10]

Broad self-reported measures of health and wellbeing such as the SF36 and GHQ attempt to capture this heterogeneity, but do not consider key capacities (for example cognitive capacity), and can have difficulty distinguishing between the contribution of individual or environmental level factors to functional status.

Distinguishing between capacity and ability is also a problem for other commonly used measures of overall functioning in older age including Instrumental Activities of Daily Living (IADLs) or Activities of Daily Living (ADLs). Losses of IADLs and ADLs are also generally only observed with very significant decrements of functioning,[11] while the WHO model suggests that changes in capacity are likely to start much earlier in life. Understanding the factors that influence levels and trajectories of overall capacity in relatively robust people before they experience these significant losses may help identify interventions earlier in the life course, and could be useful in self-care and clinical practice. Broad based outcomes like this could be useful in other ways too - for example as a way of comparing the relative benefits of interventions on different functional domains or in different organ systems.

Continuous measures of intrinsic capacity that are sensitive to subtle changes and that distinguish between the individual and their context would thus enable a much better understanding of functioning at both a population and individual level. However, this would first require a clearer conceptualisation of the intrinsic capacity construct.

To progress work in this area, we examined data from the English Longitudinal Study on Ageing (ELSA) to assess whether a range of commonly collected biomarkers and self-reported measures might provide a useful estimate of intrinsic capacity, and whether this construct predicted subsequent outcomes in relatively robust older people after accounting for the number of health conditions a participant may be experiencing. We examined the factor structure of the total capacity score to identify relevant sub factors and used structural equation modelling to assess longitudinally the direct and indirect relationships of the total intrinsic capacity score, personal characteristics and multimorbidity with subsequent IADL or ADL loss.

METHODS

Study description:

The English Longitudinal Study of Ageing (ELSA) is an ongoing study of a nationally representative sample of the English population aged ≥50 years[12]. Participants were recruited from households that were included in the Health Survey for England in 1998, 1999 and 2001, and then followed up every 2 years with detailed health examinations through nurse visits taking place every 4 years. Data were collected via face-to-face assessments using computer-assisted personal interviews and a self-completion questionnaire. In addition, a trained nurse visited participants in waves two, four, and six to measure physical functioning and collected the blood samples which were then analyzed to generate biomarker data. In ELSA the response rates varied across the waves with 67 % in wave 1, 82 % in wave 2, 73 % in wave 3, 74 % in wave 4 and 80 % in wave 5 [12]. The inclusion criteria for the present study include a) participants aged over 60 years included in the nurse visit, b) consent to provide blood sample, c) no missing data on main exposure (intrinsic capacity) indicators, and d) follow-up outcome data available in wave 5 (2010/2011). Applying these criteria led to a total study sample of 2352 participants (Figure 1).

Patient involvement: All participants were required to provide informed written consent. All ELSA data are anonymous and freely accessible from the UK Data Service Discover. Only data contained within the ELSA database were included in the analyses. No patients were involved in the development of the research question, study design or interpretation of the data in this study.

For peer review only

Measures

Intrinsic capacity:

We considered measures collected in ELSA that might provide objective estimates of aspects of intrinsic capacity based on the following criteria: a) prior evidence supporting an association with at least one aspect of capacity, and b) ability to distinguish between high and low physical or mental capacity at older ages and sensitivity to detect change within and between individuals over time.

Walking speed: Each participant aged 60 and above was eligible for the *timed walk* test. In addition, prior to the actual test, respondents were asked if they had any problems from recent surgery, injury, or other health conditions that might prevent them from walking. Only persons aged at least 60 years, willing to do the test, and able to walk (walking aids were permitted) were asked to walk 8 feet (2.4 m) at their usual walking pace, twice[13]. The time for both walks was recorded separately. In our analysis we use the mean speed (measured in m/s) of the two trials.

Chair-stand test: The chair stand test, a measure of physical performance, assessed the time required to rise from a chair to a full standing position five times with arms folded across the chest, with slower times reflecting worse function[14]. The test incorporated the use of respondent's own armless, straight backed chair. The time taken for full stand was recorded in seconds. Respondents were considered as ineligible if they could not stand up without assistance; the use of walking aids, such as a walker or cane, was not permitted. The test was stopped if the respondent became too tired or short of breath; if the participant used their hands; if after one minute, the participant had not completed all the rises; or if the nurse felt concerned for the respondent's safety.

Balance: Static balance was evaluated in three separate and progressively more difficult tests which formed part of the Short Physical Performance Battery[15]. Participants were ineligible for the tests if they were chair-bound or wheelchair-based; if it became clear after discussion that they were too unsteady on their feet; if they found it painful to stand; or if either the nurse or the participant considered the test unsafe. We used three components of the balance test (an additional two components were performed by younger participants only): side-by-side, semi-tandem, and full tandem. A) Side-by-side stand: Participants were asked to stand with feet together, side-by-side, for at least 10 seconds, using their arms, bending their knees or moving their body to maintain balance, but not moving their feet. If the participant was unable to hold the position for 10 s, a score of zero was recorded and no further tests attempted. Those able to hold the position for 10 s moved on to the semi-tandem stand. B) Semi-tandem stand: Participants had to stand with the side of the heel of one foot touching the big toe of the other foot for at least 10s. Participants unable to hold the position for 10 s scored one and no further tests were attempted. Those able to hold the position for 10 s moved on to the full-tandem stand. C) Full-tandem stand: For this test, participants had to stand with the heel of one foot in front of and touching the toes of the other foot. Those unable to hold this position for at least 3s scored no additional points; those able to hold the position for at least 3 but less than 10 s scored one point for this test; and those able to hold the position 10 s or longer scored two points for this test. The maximum possible score from all three tests was four points: one point each from the side-by-side and semi- tandem tests, and two points from the full-tandem test.

Grip strength: The grip strength test is a test for upper body strength[16]. Handgrip strength (kg) of the dominant hand was assessed using a handheld dynamometer, with the average(mean) of three measures used in the analyses. Three values were recorded for each hand, starting with the non-dominant hand and alternating between hands. Any measurements carried out incorrectly or participants refused to perform the test were not included.

Forced expiratory volume: Lung function was measured using a NDD Easy On Spirometer[17]. Willing and eligible respondents were asked to stand or seated, take a deep breath and blow into the spirometer as hard as they

For peer review only

could. Respondents were then required to repeat the procedure to give three technically satisfactory blows. The highest technically satisfactory measure of forced expiratory volume in 1 second (FEV1) was used in the analysis. The protocol required three successful measurements to be completed. An unsatisfactory blow included any of the following: an unsatisfactory start with excessive hesitation; laughing or coughing, especially during the first second; a Valsalva manoeuvre; leakage of air around the mouthpiece; obstruction of the mouthpiece by tongue or teeth; obstruction of the spirometer flow head outlet by hands.

Blood assay: A trained nurse collected biomarker data from all participants not meeting exclusion criteria. Viable blood samples were obtained from 6188 respondents (75.6% of wave 4 participants). Detailed information on the technicalities of the blood analysis, the internal quality control and the external quality assessment for the laboratory have been described elsewhere[18]. Dehydroepiandrosterone DHEA (S) levels from serum was performed using the Roche DHEA(S) assay that is a competitive immunoassay using electrochemiluminescence technology (analytical range: 0.003–27 μmol/L)[19]. Haemoglobin level (g/dl) was measured with two Abbott Diagnostics Cell-Dyn 4000 analysers[20]. IGF-1(Insulin-like growth factor 1) values are reported as whole numbers (range: 3–200 nmol l−1)[21].

Sensory: Hearing and vision impairments were measured using self-reported [22 23], validated questions previously demonstrated to be accurate when compared with objective measures. Hearing status was assessed by asking participants to rate their hearing (using a hearing aid if they used one) as excellent, very good, good, fair, or poor. For vision, participants were also asked 'How good is your eyesight for seeing things at a distance, like recognising a friend across the street' and 'How good is your eyesight for seeing things up close, like reading ordinary newspaper print'. Response options (excellent/very good/good/fair–poor) were categorised as above.Cognitive*:* The ELSA data include scores on three tests of cognitive function: verbal fluency, delayed verbal memory, and attention[24]. Verbal (semantic) fluency was assessed by asking participants to name as many animals as they could think of in 1 minute. Delayed verbal memory was assessed using lists of nouns presented aurally. Attention was assessed using a letter cancellation task. Scores on these tests were used as measures of three kinds of cognitive function: the scores on the animal naming task were taken as a measure of executive function[25], the sum of the scores on the delayed recall tasks were taken as a measure of memory, and the scores on the letter cancellation task were taken as a measure of processing speed[26].

Affect*:* Affect was measured using the eight-item Center for Epidemiological Studies-Depression (CES-D) scale[27]. Five of the eight CES-D items (i.e. felt depressed, was happy, felt lonely, enjoyed life, felt sad) were depressed mood items, while the remaining three (i.e. everything was an effort, restless sleep, and could not get going) were somatic complaints items. We derived a summary CES-D score by adding responses to all eight dichotomous questions (possible range:0-8).

Sleep: To assess sleep disturbance, participants were asked about the frequency of delay in falling asleep, inability to stay asleep, waking up tired, and disturbed sleep in the previous month[28]. Response categories were no difficulties, less than once a week, once or twice a week and three times or more a week. These response codes were given a numerical score (1 to 4) and then items were summed and a total score created. The total score ranged between 4 and 16, and showed a normal distribution, with a mean score of 8.8 (standard deviation 3.2).

Other covariates:

We also extracted data on other sociodemographic and medical covariates, recorded at wave 4, that may potentially confound the associations between intrinsic capacity and care dependence. These included chronological age, sex, education (no education, intermediate and higher education), total non-pension net wealth in quintile as a proxy measurement of socioeconomic status and multimorbidity (self-reported information on doctor diagnosed diabetes, hypertension, stroke, heart diseases (myocardial infarction, congestive heart failure, angina), chronic obstructive pulmonary disease , asthma, arthritis,

For peer review only

osteoporosis, cancer, Parkinson's disease, Alzheimer's disease and other dementia[29].

Measures of outcome:

Care dependence: The outcome of interest for longitudinal analysis – incident care dependence - was chosen because it was an overall measure of functioning that was assessed independently from the functional characteristics included in the intrinsic capacity construct. Care dependence was assessed using self-reported limitations in the Basic Activities of Daily Living (ADL) and Instrumental Activities of Daily Living (IADL)[30]. Respondents were asked to exclude any difficulties expected to last less than 3 months. ADL included six activities: dressing, walking across a room, bathing or showering, eating, getting in or out of bed, using the toilet. IADL included seven activities: using a map to get around in a strange place, preparing a hot meal, shopping for groceries, making telephone calls, taking medications, doing work around the house or garden and managing money. The scales ranging from 0 to 6 for ADL and 0 to 7 for IADL (number of items with reported difficulty) were constructed. To enable us to identify the incident loss of ADLs and IADLs, adults with limitations at wave 4 were excluded from the baseline analysis.

Statistical analysis

All statistical analysis was performed using Mplus version 8 [31] and Stata 14[32]. We performed incrementally related structural equation models (SEMs): a) traditional exploratory factor analysis, b) exploratory bi-factor analysis(EFA), c) confirmatory factor analysis(CFA), and d) mediation and moderation analysis.

We first performed a conventional exploratory factor analysis to reveal sub-factors of the intrinsic capacity concept using the robust weighted least squares (WLSMV) method. Eigen value and scree plot were used to identify number of sub-factors to retain. Communalities ≥0.3 was selected for minimum loading of an item. We then conducted a bi-factor analysis to examine the possibilities of establishing one general factor (Intrinsic capacity). The bi-GEOMIN rotation was implemented that allowed specific sub-factors to be correlated with the general factor (intrinsic capacity) and also correlated with each other. The factor structure was further tested in the confirmatory factor analysis. We identified the best fitting model using the inferential goodness-of-fit index in combination with several descriptive indices: root mean square error of approximation (RMSEA), comparative fit index (CFI), Tucker–Lewis Index (TLI). CFI and TLI values of greater than 0.9 and a RMSEA of less than 0.8 suggest a moderate fit, where as a CFI and TLI of greater than 0.95 and a RMSEA of less than 0.6 suggest a very good fit[33]. For the bifactor model, we calculated omega hierarchical coefficients (ωH), because in the bifactor model the indicators are assumed to be influenced by both the general factor and the specific factors[34].

We tested the construct validity of the general factor(intrinsic capacity) and specific sub-factors in regression analysis. The summary scores for general factor and specific sub-factors were generated from CFA by fixing the latent mean to 0 and the latent standard deviation to 1 for each factor. The scores of specific sub-factors can be interpreted as the unique contribution of each of the specific domains "over and above" the general factor (intrinsic capacity). These summary scores were used in the linear regression for testing the construct validity. Simple t-test were performed to examine the statistical difference in the intrinsic capacity score among older persons with or without chronic diseases and results are summarized by age-group and overall population score in two-way boxplot.

Finally, we assessed the predictive validity of the intrinsic capacity score in a mediation model of the direct and indirect relationships of intrinsic capacity and multimorbidity with incident loss of ADLs and IADLs, after controlling for all personal characteristics[35]. PM (ratio of the indirect effect to the total effect) and Rm (ratio of the indirect effect to the direct effect) was calculated to examine the indirect effect size in the mediation analysis[36 37]. For visualizing moderation effects, we used the Johnson-Neyman technique[38]. A bias-corrected bootstrap method was used for drawing inference in mediated and moderated analysis [35].

For peer review only

RESULTS

Sample characteristics

Baseline levels of study variables are presented in supplementary table 1s (online). Of the 7321 potential participants at baseline, 33% reported either ADL or IADL difficulties and 26 % did not provide consent for blood sample analysis (Figure 1). A further 28% of the remaining 3581 participants, participants had incompleteinformation on the independent variables and were also excluded from analysis. The baseline sample therefore comprised 2560 eligible participants. Compared to participants included in the baseline analysis, participants without complete information were older, had a lower education attainment and reported more chronic conditions.

In the follow-up, 91% of baseline eligible participants were re-interviewed. Except education, there was no difference on age, sex, wealth, and multimorbidity status among participants interviewed and not interviewed at the follow-up (Table s1).No imputation was performed in the analysis and participants with missing data were excluded, leaving a study sample of 2560 with complete data for the EFA and CFA analysis.

Bi-factor EFA, CFA and model fit

In the initial exploratory factor analysis, the Kaiser eigenvalues criterion suggested a five-factor model, with 5 factors having Eigen values greater than 1 (i.e.3.1,2.3,1.61, 1.23,1.04). These five factors explained 86% of total variance among the intrinsic capacity indicators. Supplementary table 2s shows the model fit information for EFA and CFA models tested in the study. One to three factor models provided unacceptable degrees of fit to the data, whereas five factor models provided very good fit, which suggests that intrinsic capacity is a multidimensional construct.

Next, we performed bi-factor EFA under a SEM framework to identify potential modelling problems (e.g. sizable cross loading of intrinsic capacity indicators) and get an early insight on whether primary results of EFA could be replicated with bi-factor model perspectives of multidimensionality. Most items loaded well (≥0.3) on the general factor (intrinsic capacity). Bi-factor EFA revealed one general factor (IC) and five specific sub-factors that we labelled cognitive, sensory, vitality, locomotor, and psychological (supplementary table 3s). The model fits the data very well: chi-square = 71.2 (df = 39), RMSEA = 0.012 (90% CI 0.011 to 0.024), CFI = 0.99 and TLI = 0.99 (Supplementary table 2s). When we examined the factor structure (one factor, second-order, correlated, bi-factor models) in confirmatory factor analysis, the pattern of factor loadings for the bi-factor CFA model showed a clear, simple structure with the five sub-factors (Figure 2).

Within the bifactor CFA model, excluding two sub-factors (sensory and locomotor), the factor loadings were evenly shared between the general factor and sub-factors. However, indicators in the psychological (sleep) and sensory (near vision and distance vision) sub-factors had higher loadings on their group factor than on the general factor (intrinsic capacity). This suggests that these two sub-factors provide additional information about psychological and sensory capacity, after accounting for the variance of the general factor. The model achieved a good fit for the data: chi-square value =1180.6(df=89), RMSEA =0.035 (90% CI 0.033 to 0.037), CFI =0.98 and TLI=0.97(table 2s). Indeed, the bi-factor model fit was stronger than for the second order factor model: chi-square value =2369 (df=102), RMSEA =0.07, CFI =0.94 and TLI=0.92. Taken together, these findings support this bi-factor model with one general factor representing overall intrinsic capacity and five specific sub-factors.

Reliability of the factor scores

The ωH (hierarchical) coefficient was calculated to understand the reliability of a latent general factor (Intrinsic capacity). The ὼh value for the general factor was 0.78, and the ωHS(sub-score) values for specific factors were .0.79, 0.80,0.81,0.82, and 0.83, respectively. A ωH value more than 0.7 indicates that the intrinsic

For peer review only

capacity total score predominantly reflects a single general factor, suggesting that the total score can be interpreted as a reliable measure of intrinsic capacity. The ωHS more than 0.80 for the sub-factor suggests that domain specific scores are equally reliable as the general factor score. Independent of specific factors, the percentage of reliable variance in the score due to the general factor was 72%. This indicates that the intrinsic capacity summary score was a sufficiently reliable measure of the general factor, and added value beyond sub-factor scores.

Construct validity

Factors associated with intrinsic capacity (general factor) and sub-domains (sub-factors) are presented in the supplementary table 4s. Lower intrinsic capacity scores were significantly associated with increasing age, female sex, lower levels of education, lower wealth, number of chronic diseases, and number of ADL and IADL limitations. Even after mutual adjustment, all related constructs remained statistically associated with intrinsic capacity (see Figure 3). Since all these characteristics have previously been associated with poorer health in older age, these findings support the construct validity of the general factor.

Associations between intrinsic capacity score and other variables

We used a boxplot of intrinsic capacity score for each chronic condition over three different age group to display associations between specific chronic conditions and intrinsic capacity s (Figure 4). Overall, older adults with chronic conditions had statistically significantly lower intrinsic capacity scores (below the mean) than those without chronic conditions and this association was stronger in older age groups. However, the impact of different chronic conditions on the intrinsic capacity scores varied. The greatest impact on intrinsic capacity score was from dementia in the two older age groups. We also examined the intrinsic capacity scores among older people with no chronic conditions in different age-groups. We found that in the absence of any diagnosed chronic conditions, the intrinsic capacity scores tend to decline in higher age-groups. In other words, older people with no diagnosed chronic conditions in higher age-groups (70-79 and 80-100) had significantly lower intrinsic capacity scores than older people in young age-group 60-69 years.

In a separate correlation analysis, we found associations between specific factor scores and various personal characteristics or multimorbidity and these associations were generally consistent with previous research on these characteristics (table 4s). Cognitive factor scores were negatively associated with increasing age, number of multimorbidities and positively associated with female sex, higher education, and wealth (highest quantile). Locomotor scores were negatively associated with age and multimorbidity, and positively associated with higher education, wealth, and female sex. Psychological factor scores were negatively associated with increasing age and higher multimorbidity. Higher psychological factor scores were negatively associated with age, female sex and multimorbidity. Vitality sub-factor scores were negatively associated with increasing age and multimorbidity, and positively associated with female sex, higher education and higher wealth. The scores of the sensory sub-factor were positively associated only with higher education.

Pathways to care dependence

In the simple mediation model, we tested the direct effect of intrinsic capacity on the incident loss of ADLs and IADLs and the indirect effect through multimorbidity (Supplementary table 5s, Supplementary figure1s). Intrinsic capacity predicted the incident loss of ADLs and IADLs both directly and indirectly, even after controlling for age, sex, education, and wealth. In comparisons of the effect size, the direct effect of intrinsic capacity on IADL and IADL was much more prominent than the indirect mediational effect through multimorbidity. In terms of proportion, only a small proportion of the effect of intrinsic capacity on the incidence ADL (8.7%) and IADL (5.2%) occurred indirectly through multimorbidity. A bias-corrected bootstrap confidence interval for this direct and indirect effect, which was based on a 10,000-bootstrap sample, was entirely above zero, thus suggesting that these effects are statistically significant.

For peer review only

The results of serial multiple mediators modelling of the relationships between the incident loss of ADLs and IADLs and personal characteristics, intrinsic capacity scores and multimorbidity are shown in Figures 5 and 6.

Both intrinsic capacity score and multimorbidity independently predicted incident loss of ADLs, however only intrinsic capacity independently predicted incident loss of IADLs. Except age, none of the personal characteristics (sex, wealth and education) had a direct effect on incident loss of ADLs and IADLs (supplementary table 6s). Personal characteristics were strongly associated with both intrinsic capacity and multimorbidity, and the relationship between all personal characteristics (including chronological age) and the incident loss of ADLs and IADS operated through multimorbidity or intrinsic capacity. A greater proportion of the impact of age on outcomes (30% for ADLs and 39% for IADLs) occurred indirectly through intrinsic capacity than directly (24% for both ADLs and IADLs).

The specific indirect effect of all personal characteristics (age, sex, education, and wealth) on the incident loss of ADL and IADL through intrinsic capacity was statistically significant (Table 6s). None of the indirect effect of personal characteristics on incident loss of IADLs operating through multimorbidity was statistically significant. This implies that specific indirect effects of personal characteristics on IADL were mainly transmitted through intrinsic capacity rather than multimorbidity.

In a moderation analysis, after including the interaction term (age*intrinsic capacity), the direct effect of chronological age on incident IADL was not statistically significant (-0.03, pvalue = 0.16). The effect of chronological age on IADL was moderated by a person's level of intrinsic capacity (-0.526, pvalue=0.004), with the relationship between chronological age and IADL only being significant for people with low intrinsic capacity (figure 2s). Similarly, intrinsic capacity moderated the effect of chronological age on incident loss of ADL, after controlling for personal characteristics and multimorbidity (-0.472, pvalue =0.03).

DISCUSSION:

The WHO model of *Healthy Ageing* provides a transformative framework by which to consider health in older age. Rather than using the entry points of chronological age or disease, the model is built around the concept of intrinsic capacity - all the individual level characteristics that contribute to a person's ability to be and to do what they have reason to value. However, there has been little empirical analysis of the concept and a clear understanding of a possible structure for intrinsic capacity is lacking.

We used a large longitudinal study on ageing to explore the possible structure and predictive validity of the intrinsic capacity concept. We developed a total capacity score for each study participant and found it to be a powerful predictor of incident care dependence, even after accounting for chronological age and the presence, or number, of key health conditions. Factor analysis suggested a structure comprising 5 sub factors - psychological, sensory, cognitive, vitality and locomotor. This may provide a frame for the construct that is readily applicable to research and clinical practice.

These findings suggest that the intrinsic capacity concept has an empirical rigour and captures information beyond that generally considered in research or clinical practice. It also suggests that multiple domains of capacity can be aggregated into a meaningful overall measure of health status. If confirmed by future studies, these findings have a number of significant implications. For example, routine monitoring of intrinsic capacity might enable clinicians to flag when trajectories of capacity in the second half of life are veering off normal - a similar approach to the way child development charts currently guide paediatric practice[39]. A recent meeting of expert geriatricians convened by WHO confirmed that this would be useful, particularly if score changes could be interpreted in ways that have clinical relevance[40]. The factor structure of capacity identified in this analysis may provide a framework that achieves this by allowing clinicians to identify and address the drivers of any changes.

For peer review only

Measurable trajectories of capacity may also be useful as research outcomes of interest. As continuous measures that can be monitored at multiple time points, they allow a more nuanced and powerful analysis than approaches that use crude categorical measures of late life events such as mortality or incident loss of ADLs and IADLs[41]. Moreover, if information was available on trajectories of capacity across the full second half of life, this may facilitate the identification of mid-life influences on late life health which may be amenable to intervention. This is likely to become more feasible with the rapid development of wearable and communications devices which are already generating large amounts of relevant and routinely collected data. Appropriate algorithms could be developed to process this information to describe trajectories of capacity that could inform self-management, clinical practice and research.

Using trajectories of capacity as a research outcome may also allow better comparison between the impacts of interventions for different conditions. Furthermore, as medicine becomes increasingly personalised and precise, better information is needed on how different subpopulations may respond to specific interventions[42]. Stratifying by intrinsic capacity may provide a useful way of identifying the groups for which interventions are most effective and may be more appropriate than categorisation by chronological age or comorbidity.

One critical issue requiring further work is that not all five subfactors appear to operate at the same level. The cognitive, locomotor, sensory and psychological sub factors can be thought of as overt expressions of capacity. On the other hand, dehydroepiandrosterone, IGF-1, haemoglobin and forced expiratory volume (included in the vitality sub factor) are elements of the biologic systems that underlie these overt manifestations of capacity[43]. Grip strength, the other characteristic loading to the vitality subfactor, can also be considered a marker of broader underlying factors such as nutritional, immune and hormonal status, and in this sense it is interesting that it loaded separately to locomotor capacity.[44 45]

The vitality sub factor interacts strongly with the other subfactors and part of the contribution it makes to the intrinsic capacity score is through the influence it has on these overt expressions of capacity. However it also loaded independently to the general factor (intrinsic capacity).

One possible conceptual frame for these relationships starts with a vitality domain describing variance in the complex and dynamic biologic systems which sustain life and functioning. When accumulated deficits in these systems reach a certain point they become manifest in the overt losses of capacity that are commonly associated with ageing. However, deficits in these systems that may not yet be expressed in overt manifestations are also likely have implications for the ability of the individual to retain their level of functioning. This residual is consistent with the notion of physiologic reserves or physiologic “resilience”. A total measure of vitality may thus capture an individual’s “biological age”.

Figure 7 shows how these domains might hypothetically relate. We have included a space for specific expressed capacities not captured in the four domains identified in our analysis (for example continence and speech). Within the vitality construct we have included cellular level characteristics as well as the contribution of higher physiologic systems. This is consistent with our analysis but also suggests at how characteristics not assessed (see strengths and weaknesses) might be considered in a conceptual frame for intrinsic capacity.

For peer review only

A second issue is that all the more overt capacities also interact. This could be explained using the conceptual frame proposed above since the biologic drivers of these capacities are shared. This finding is also consistent with research and clinical experience which suggest that decrements in one domain of capacity may have clinically relevant impacts on other domains. For example, gait speed can be influenced by simultaneously drawing on an individual's cognitive capacity (e.g. by being asked to count backwards). These complex interactions may indeed provide the opportunity for "stress" testing of scores in any single domain[46].

However, the combined score we have calculated takes no account of thresholds that may exist within each subfactor. For example, cognitive capacity may fall to the point where it becomes impossible for an individual to survive without appropriate care and support, even though they may retain perfect capacity in each other domain and thus retain a relatively high total capacity score. This emphasises the need to assess the multiple dimensions of capacity to fully assess the clinical importance of changes in total score.

Strengths and limitations of study

A strength of this study is that it is a large, nationally representative sample of older people living in England with good follow-up. Unlike approaches that use a composite total score which assumes that each indicator or measure contributes equally to the general factor (i.e. intrinsic capacity), we used the bifactor model scores that represents a pure measure of the underlying latent trait of interest, after controlling for all five specific sub-factors [47]. Hence, a theoretically error-free score was used in all analysis to study the unique contribution of intrinsic capacity and its components in the prevention of care dependence.
Secondly, the longitudinal nature of the study allowed us to examine the direction of causality. Thirdly, most of the indicators of intrinsic capacity were measured using objective performance tests, limiting opportunities for response or interviewer bias.

However, it is important to note that the measures included in the ELSA study are neither complete nor random. They were chosen to inform specific research questions of interest to the investigators, rather than to create an overall measure of intrinsic capacity[12]. Additional variables may alter the total capacity scoring and the factor structure. Nevertheless, since these questions largely draw on existing knowledge and research priorities, they cover aspects of most domains that might be conceptualised within the notion of capacity. Some potential components of capacity cannot be readily measured objectively (for example energy levels). Others require complex assessments that are beyond the scope of primary care or population-based research (for example, continence, cardiovascular capacity). Changes in other important attributes like the capacity for speech are important but less common. A number of key biomarkers, for example telomere length and immune function, were also missing from this dataset. Thus, while the set of indicators considered in this analysis can be considered relatively comprehensive, they are not complete in their ability to measure all aspects of capacity. Moreover, while we attempted to limit analysis to objective measures, the only data available on sensory and psychological capacities was through self report. This should not have had a significant impact on the construct of capacity, but may have had a marginal influence on the longitudinal analysis we undertook.

Despite carefully accounting for potential confounders, measurement error in their assessment, particularly the difference between participants who could and could not provide complete information on all exposure measures, may have biased associations. Also, the number of chronic diseases included in the analysis are limited, hence there is possibility of residual confounding.

Our findings are, however, consistent with previous research on the sub factors that were included in our analysis. Several longitudinal studies have shown strong predictive validity of cognitive (namely memory and executive function) [48 49], locomotor (gait or chair rise)[50-52], sensory (vision and hearing)[23 53-55], vitality (hand grip strength or FEV)[56-59], and psychological [60] indicators in relation to incident loss of ADL and IADL. Studies have also demonstrated associations between indicators of intrinsic capacity and survival. In particular, studies

of locomotor and cognitive functions have shown that these indicators are predictors of premature mortality in community dwelling populations[61-63] . Yet, traditionally, these characteristics have often been considered independently. The intrinsic capacity concept provides a vehicle for assessing how they relate to each other and a possible approach to better quantify ambiguous notions such as "health" in older age into research and clinical practice[40 64].

Conclusions

Measurement of intrinsic capacity is feasible with commonly used measures and appears to provide useful predictive information on an individual's subsequent functioning. The proposed general factor and sub-factors structure may contribute to a transformative paradigm for future research and clinical practice.

Figure legend

Figure 1: Flow of study members into the analytical sample: the English Longitudinal Study of Ageing.

Figure 2: Bi-factor CFA model of Intrinsic Capacity

Figure 3: Construct validity of Intrinsic capacity (mutually adjusted)

Figure 4: Intrinsic capacity summary score by chronic health conditions and age-group

Figure 5: Direct and indirect effect of characteristics on activities of daily living

Figure 6: Direct and indirect effect of characteristics on instrumental activities of daily living

Figure 7: Conceptual frame for the construct of Intrinsic Capacity

Contributorship: JRB conceived of the research, oversaw analysis and was responsible for final drafting of the paper. JAT undertook all analyses, reviews of related literature, and contributed to drafting of the paper. MC contributed to conceptualisation, reviews of related literature and drafting of the paper. IAC contributed to conceptualisation, reviews of related literature and drafting of the paper. All authors reviewed and approved the final manuscript submitted for publication.

Funding: This research received no specific grant from any funding agency in the public, commercial or not-for-profit sectors.

Patient consent: The study uses secondary data from ELSA available publicly. All participants gave full informed written consent for participation in the study.

Ethical approval: Ethical approval for ELSA was obtained from NHS Research Ethics Committees under the National Research and Ethics Service (NRES), and participants gave full informed written consent for participation. More information on ELSA can be found at http://www.ifs.org.uk/elsa/documentation.php.

For peer review only

Declaration of interests: The authors received no support from any organisation for the submitted work; have no financial relationships with any organisations that might have an interest in the submitted work in the previous three years; and have no other relationships or activities that could appear to have influenced the submitted work.

Acknowledgments: The views expressed in this paper are those of the authors and do not necessarily reflect the views of WHO. The authors would like to thank the ELSA participants, the ELSA researchers and the UK Data Service for enabling the use of ELSA data for this analysis.

Data sharing statement: ELSA dataset and information on all currently archived can be freely accessed through the UK Data Archive (https://www.elsa-project.ac.uk/availableData).

For peer review only

References :

1. WHO. World Report on Ageing and Health. In: John Beard AO, Andrew Cassels, ed. Geneva, Switzerland Department of Ageing and Life Course ,World Health Organization, 2015.
2. Beard JR, Officer A, de Carvalho IA, et al. The World report on ageing and health: a policy framework for healthy ageing. The Lancet 2016;**387**(10033):2145-54.
3. Guralnik JM, Ferrucci L. Assessing the building blocks of function: utilizing measures of functional limitation. Am J Prev Med 2003;**25**(3 Suppl 2):112-21.
4. Guralnik JM, Ferrucci L. Assessing the building blocks of function: Utilizing measures of functional limitation. American Journal of Preventive Medicine 2003;**25**(3, Supplement 2):112-21.
5. Ben-Shlomo Y, Cooper R, Kuh D. The last two decades of life course epidemiology, and its relevance for research on ageing. Int J Epidemiol 2016;**45**(4):973-88.
6. Belsky DW, Caspi A, Houts R, et al. Quantification of biological aging in young adults. Proceedings of the National Academy of Sciences 2015;**112**(30):E4104-E10.
7. Gershon RC, Wagster MV, Hendrie HC, et al. NIH Toolbox for Assessment of Neurological and Behavioral Function. Neurology 2013;**80**(11 Supplement 3):S2-S6.
8. Chatterji S, Byles J, Cutler D, et al. Health, functioning, and disability in older adults—present status and future implications. The Lancet 2015;**385**(9967):563-75.
9. Ferrucci L, Cooper R, Shardell M, et al. Age-Related Change in Mobility: Perspectives From Life Course Epidemiology and Geroscience. The Journals of Gerontology: Series A 2016;**71**(9):1184-94.
10. Milot E, Morissette-Thomas V, Li Q, et al. Trajectories of physiological dysregulation predicts mortality and health outcomes in a consistent manner across three populations. Mechanisms of Ageing and Development 2014;**141-142**:56-63.
11. Fieo RA, Austin EJ, Starr JM, et al. Calibrating ADL-IADL scales to improve measurement accuracy and to extend the disability construct into the preclinical range: a systematic review. BMC Geriatrics 2011;**11**(1):42.
12. Steptoe A, Breeze E, Banks J, et al. Cohort profile: the English longitudinal study of ageing. Int J Epidemiol 2013;**42**(6):1640-8.
13. Weber D. Differences in physical aging measured by walking speed: evidence from the English Longitudinal Study of Ageing. BMC Geriatr 2016;**16**:31.
14. Jones CJ, Rikli RE, Beam WC. A 30-s chair-stand test as a measure of lower body strength in community-residing older adults. Research quarterly for exercise and sport 1999;**70**(2):113-9.

15. Guralnik JM, Simonsick EM, Ferrucci L, et al. A short physical performance battery assessing lower extremity function: association with self-reported disability and prediction of mortality and nursing home admission. J Gerontol 1994;**49**(2):M85-94.
16. Dodds RM, Syddall HE, Cooper R, et al. Grip strength across the life course: normative data from twelve British studies. PLoS One 2014;**9**(12):e113637.
17. Yohannes AM, Tampubolon G. Changes in lung function in older people from the English Longitudinal Study of Ageing. Expert Rev Respir Med 2014;**8**(4):515-21.
18. Graig R, Deverill C, Pickering K. Quality control of blood, saliva and urine analytes. In: J; SKM, ed. Health survey for England 2004, Methodology and documentation. London: The Information Centre, 2006:34-41.
19. Souza-Teodoro LH, de Oliveira C, Walters K, et al. Higher serum dehydroepiandrosterone sulfate protects against the onset of depression in the elderly: Findings from the English Longitudinal Study of Aging (ELSA). Psychoneuroendocrinology 2016;**64**:40-6.
20. Grimaldi E, Scopacasa F. Evaluation of the Abbott CELL-DYN 4000 hematology analyzer. Am J Clin Pathol 2000;**113**(4):497-505.
21. Newcastle. FL. DPC Immulite 2000—IGF-1 (Laboratory summary of methods used up to 02 February 2012, available from laboratory on request). United Kingdom, 2012.
22. Liljas AEM, Carvalho LA, Papachristou E, et al. Self-reported vision impairment and incident prefrailty and frailty in English community-dwelling older adults: findings from a 4-year follow-up study. J Epidemiol Community Health 2017.
23. Liljas AEM, Carvalho LA, Papachristou E, et al. Self-Reported Hearing Impairment and Incident Frailty in English Community-Dwelling Older Adults: A 4-Year Follow-Up Study. J Am Geriatr Soc 2017;**65**(5):958-65.
24. Steel N, Huppert FA, McWilliams B, et al. Physical and cognitive function. In: J MMBJBRLCN, ed. Health, Wealth and Lifestyles of the Older Population in England: the 2002 English Longitudinal Study of Ageing. London: Institute of Fiscal Studies, 2003:249-300.
25. Shao Z, Janse E, Visser K, et al. What do verbal fluency tasks measure? Predictors of verbal fluency performance in older adults. Frontiers in Psychology 2014;**5**:772.
26. Batty GD, Deary IJ, Zaninotto P. Association of Cognitive Function With Cause-Specific Mortality in Middle and Older Age: Follow-up of Participants in the English Longitudinal Study of Ageing. American Journal of Epidemiology 2016;**183**(3):183-90.
27. Carleton RN, Thibodeau MA, Teale MJ, et al. The center for epidemiologic studies depression scale: a review with a theoretical and empirical examination of item content and factor structure. PLoS One 2013;**8**(3):e58067.
28. Jenkins CD, Stanton BA, Niemcryk SJ, et al. A scale for the estimation of sleep problems in clinical research. J Clin Epidemiol 1988;**41**(4):313-21.
29. Dhalwani NN, O'Donovan G, Zaccardi F, et al. Long terms trends of multimorbidity and association with physical activity in older English population. Int J Behav Nutr Phys Act 2016;**13**:8.
30. Chan KS, Kasper JD, Brandt J, et al. Measurement equivalence in ADL and IADL difficulty across international surveys of aging: findings from the HRS, SHARE, and ELSA. J Gerontol B Psychol Sci Soc Sci 2012;**67**(1):121-32.
31. M*plus* [program]. 8 version. Los Angeles: Muthén & Muthén, 2017.
32. Stata Statistical Software [program]. 14 version. TX: StataCorp LLC 2015.
33. Hooper DC, J ; Mullen, M. Structural Equation Modeling: Guidelines for Determining Model Fit. Electronic Journal on Business Research Methods 2008;**6**(1):53-60.
34. Schweizer. K, DiStefano. C. *Principles and Methods of Test Construction: Standards and Recent Advances*: Hogrefe Publishing, 2016.

For peer review only

35. Hayes. AF. *Introduction to Mediation, Moderation and Conditional Process Analysis: A Regression Based Approach*. New York: The Guilford Press, 2013.
36. Preacher KJ, Kelley K. Effect size measures for mediation models: quantitative strategies for communicating indirect effects. Psychol Methods 2011;**16**(2):93-115.
37. Miocevic M, O'Rourke HP, MacKinnon DP, et al. Statistical properties of four effect-size measures for mediation models. Behavior research methods 2017.
38. J.; JPN. Tests of certain linear hypotheses and their applications to some educational problems. Statistical Research Memoirs 1936(1):57-93.
39. de Onis M, Onyango A, Borghi E, et al. Worldwide implementation of the WHO Child Growth Standards. Public Health Nutr 2012;**15**(9):1603-10.
40. WHO. Clinical Consortium on Healthy Ageing. Secondary Clinical Consortium on Healthy Ageing 2016. http://www.who.int/ageing/health-systems/clinical-consortium-meeting/en/.
41. Mlinac ME, Feng MC. Assessment of Activities of Daily Living, Self-Care, and Independence. Archives of Clinical Neuropsychology 2016;**31**(6):506-16.
42. Morley JE, Vellas B. Patient-Centered (P4) Medicine and the Older Person. J Am Med Dir Assoc 2017;**18**(6):455-59.
43. Belsky DW, Caspi A, Houts R, et al. Quantification of biological aging in young adults. Proceedings of the National Academy of Sciences of the United States of America 2015;**112**(30):E4104-10.
44. Norman K, Stobäus N, Gonzalez MC, et al. Hand grip strength: Outcome predictor and marker of nutritional status. Clinical Nutrition 2011;**30**(2):135-42.
45. Visser M, Deeg DJH, Lips P. Low Vitamin D and High Parathyroid Hormone Levels as Determinants of Loss of Muscle Strength and Muscle Mass (Sarcopenia): The Longitudinal Aging Study Amsterdam. The Journal of Clinical Endocrinology & Metabolism 2003;**88**(12):5766-72.
46. Montero-Odasso MM, Sarquis-Adamson Y, Speechley M, et al. Association of Dual-Task Gait With Incident Dementia in Mild Cognitive Impairment: Results From the Gait and Brain Study. JAMA Neurol 2017;**74**(7):857-65.
47. DeMars CE. A Tutorial on Interpreting Bifactor Model Scores. International Journal of Testing 2013;**13**(4):354-78.
48. Johansson MM, Marcusson J, Wressle E. Cognitive impairment and its consequences in everyday life: experiences of people with mild cognitive impairment or mild dementia and their relatives. Int Psychogeriatr 2015;**27**(6):949-58.
49. Dodge HH, Kadowaki T, Hayakawa T, et al. Cognitive impairment as a strong predictor of incident disability in specific ADL-IADL tasks among community-dwelling elders: the Azuchi Study. Gerontologist 2005;**45**(2):222-30.
50. Vermeulen J, Neyens JC, van Rossum E, et al. Predicting ADL disability in community-dwelling elderly people using physical frailty indicators: a systematic review. BMC Geriatr 2011;**11**:33.
51. Ostir GV, Markides KS, Black SA, et al. Lower body functioning as a predictor of subsequent disability among older Mexican Americans. J Gerontol A Biol Sci Med Sci 1998;**53**(6):M491-5.
52. Gill TM, Williams CS, Tinetti ME. Assessing risk for the onset of functional dependence among older adults: the role of physical performance. J Am Geriatr Soc 1995;**43**(6):603-9.
53. Cigolle CT, Langa KM, Kabeto MU, et al. Geriatric conditions and disability: the Health and Retirement Study. Ann Intern Med 2007;**147**(3):156-64.

For peer review only - http://bmjopen.bmj.com/site/about/guidelines.xhtml

54. Crews JE, Campbell VA. Vision impairment and hearing loss among community-dwelling older Americans: implications for health and functioning. American journal of public health 2004;**94**(5):823-9.
55. Lin MY, Gutierrez PR, Stone KL, et al. Vision impairment and combined vision and hearing impairment predict cognitive and functional decline in older women. J Am Geriatr Soc 2004;**52**(12):1996-2002.
56. Taekema DG, Gussekloo J, Maier AB, et al. Handgrip strength as a predictor of functional, psychological and social health. A prospective population-based study among the oldest old. Age and ageing 2010;**39**(3):331-7.
57. Al Snih S, Markides KS, Ottenbacher KJ, et al. Hand grip strength and incident ADL disability in elderly Mexican Americans over a seven-year period. Aging Clin Exp Res 2004;**16**(6):481-6.
58. Hegendorfer E, Vaes B, Mathei C, et al. Prognostic value of short-term decline of forced expiratory volume in 1 s over height cubed (FEV1/Ht3) in a cohort of adults aged 80 and over. Aging Clin Exp Res 2017.
59. Abe T ST, Yoshida H,. The relationship between pulmonary function and physical function and mobility in community-dwelling elderly women aged 75 years or older J Phys Ther Sci 2011;**23**:443-49.
60. Penninx BW, Guralnik JM, Ferrucci L, et al. Depressive symptoms and physical decline in community-dwelling older persons. JAMA 1998;**279**(21):1720-6.
61. Amuthavalli Thiyagarajan J, Bryce R, Prina M, et al. Frailty and the prediction of dependence and mortality in low- and middle-income countries: a 10/66 population-based cohort study. BMC Med 2015;**13**:138.
62. Cooper R, Kuh D, Hardy R, et al. Objectively measured physical capability levels and mortality: systematic review and meta-analysis. BMJ 2010;**341**.
63. Studenski S, Perera S, Patel K, et al. Gait speed and survival in older adults. JAMA 2011;**305**(1):50-58.
64. Cesari M, Araujo de Carvalho I, Amuthavalli Thiyagarajan J, et al. Evidence for The Domains Supporting The Construct of Intrinsic Capacity. The Journals of Gerontology: Series A 2018:gly011-gly11.

For peer review only

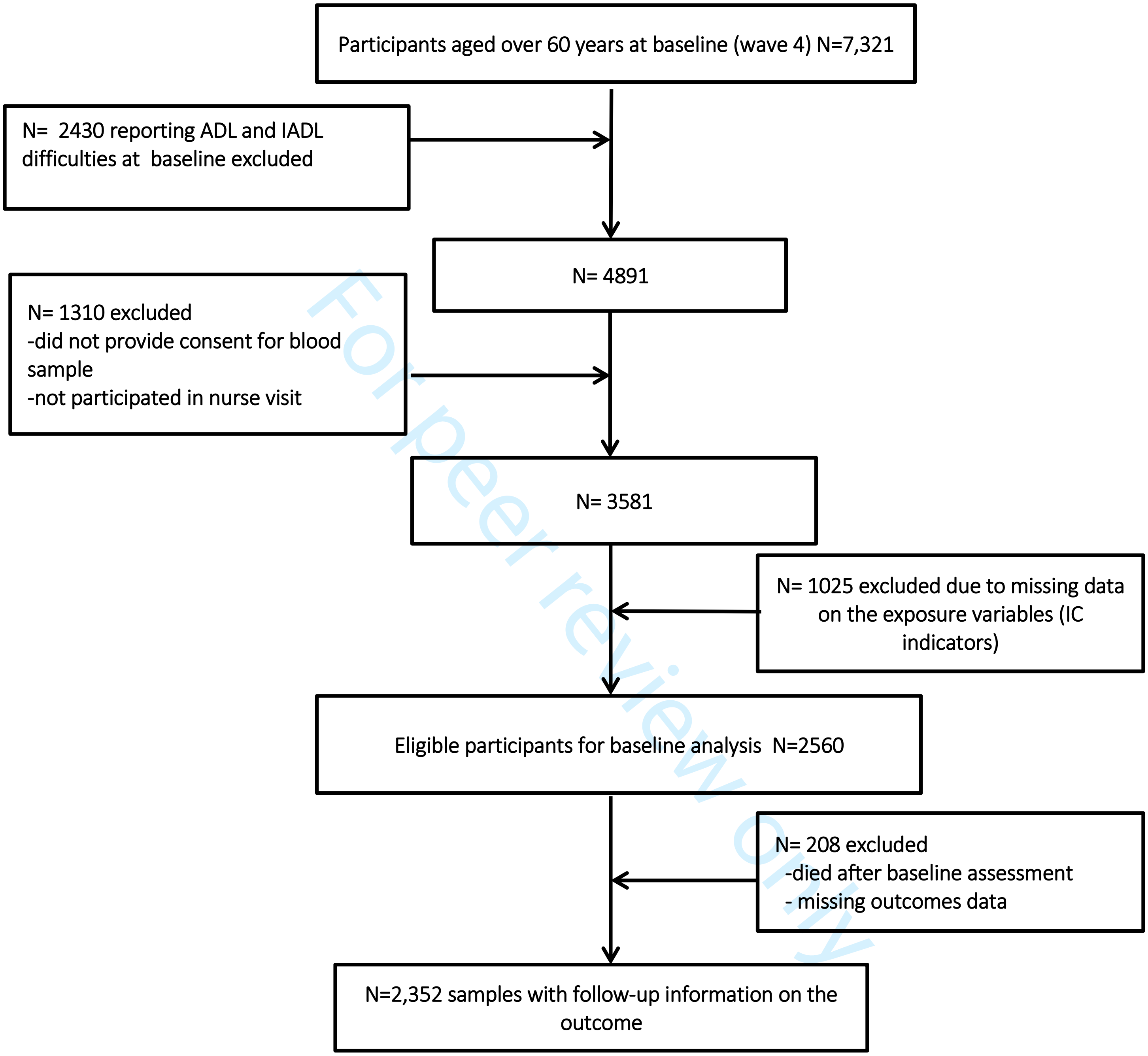
Participants aged over 60 years at baseline (wave 4) N=7,321
N= 2430 reporting ADL and IADL difficulties at baseline excluded
N= 4891
N= 1310 excluded
-did not provide consent for blood sample
-not participated in nurse visit
N= 3581
N= 1025 excluded due to missing data on the exposure variables (IC indicators)
Eligible participants for baseline analysis N=2560
N= 208 excluded
-died after baseline assessment
- missing outcomes data
N=2,352 samples with follow-up information on the outcome

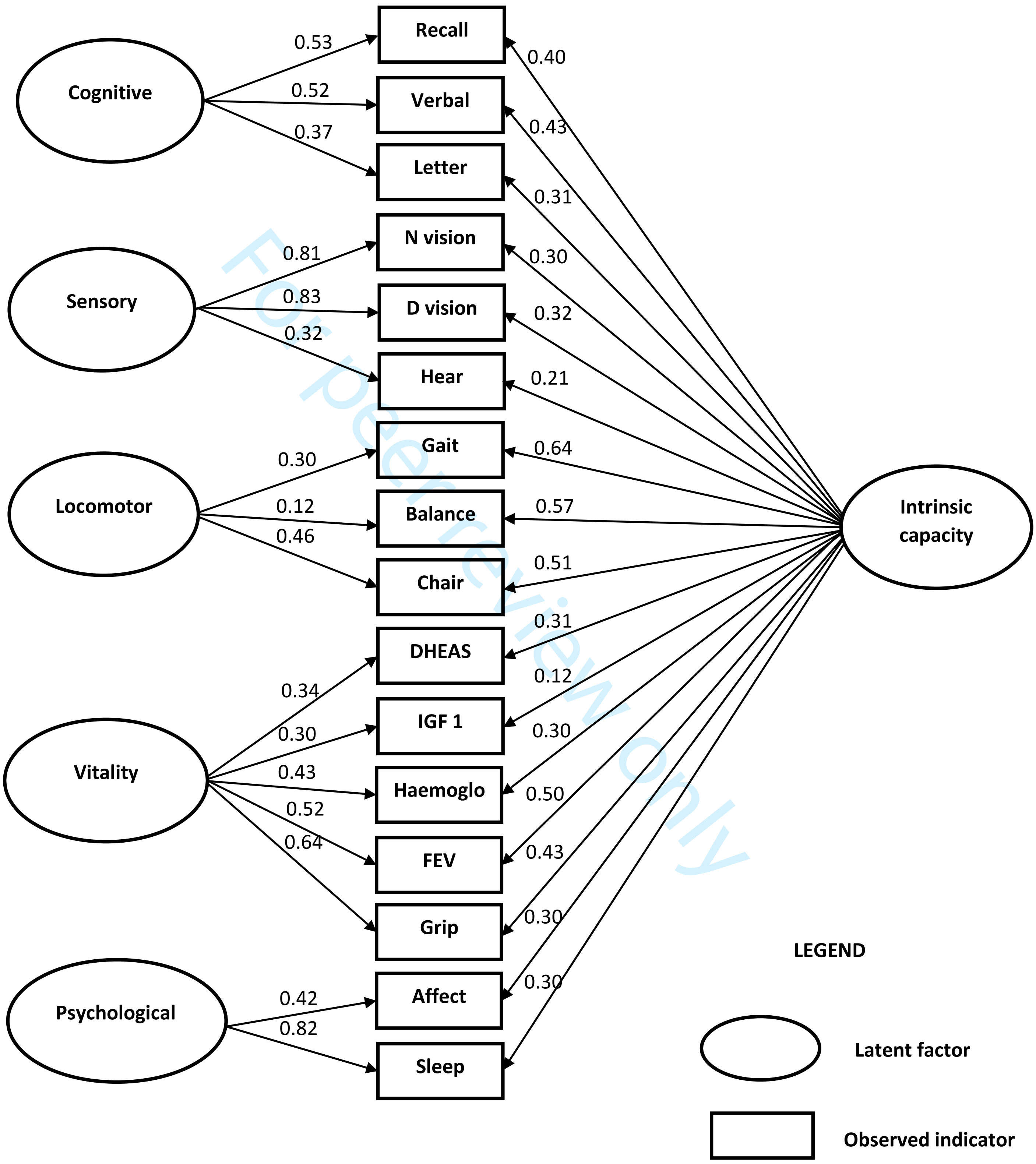
Cognitive
Sensory
Locomotor
Vitality
Psychological
Recall
Verbal
Letter
N vision
D vision
Hear
Gait
Balance
Chair
DHEAS
IGF 1
Haemoglo
FEV
Grip
Affect
Sleep
Intrinsic capacity
0.53
0.52
0.37
0.81
0.83
0.32
0.30
0.12
0.46
0.34
0.30
0.43
0.52
0.64
0.42
0.82
0.40
0.43
0.31
0.30
0.32
0.21
0.64
0.57
0.51
0.31
0.12
0.30
0.50
0.43
0.30
0.30
LEGEND
Latent factor
Observed indicator

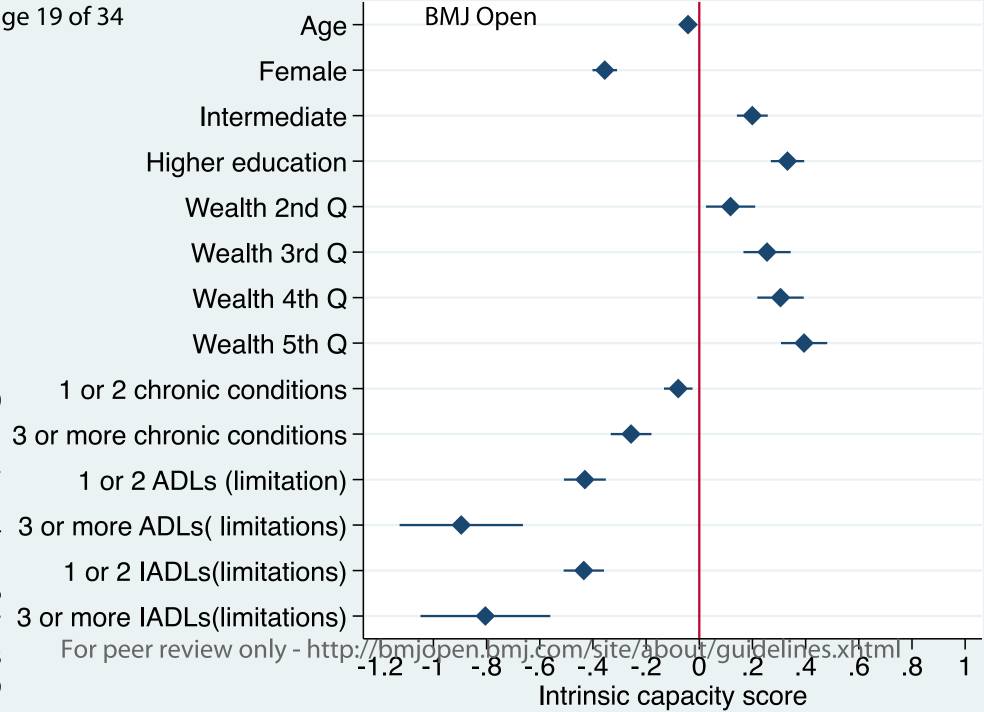

For peer review only - http://bmjopen.bmj.com/site/about/guidelines.xhtml

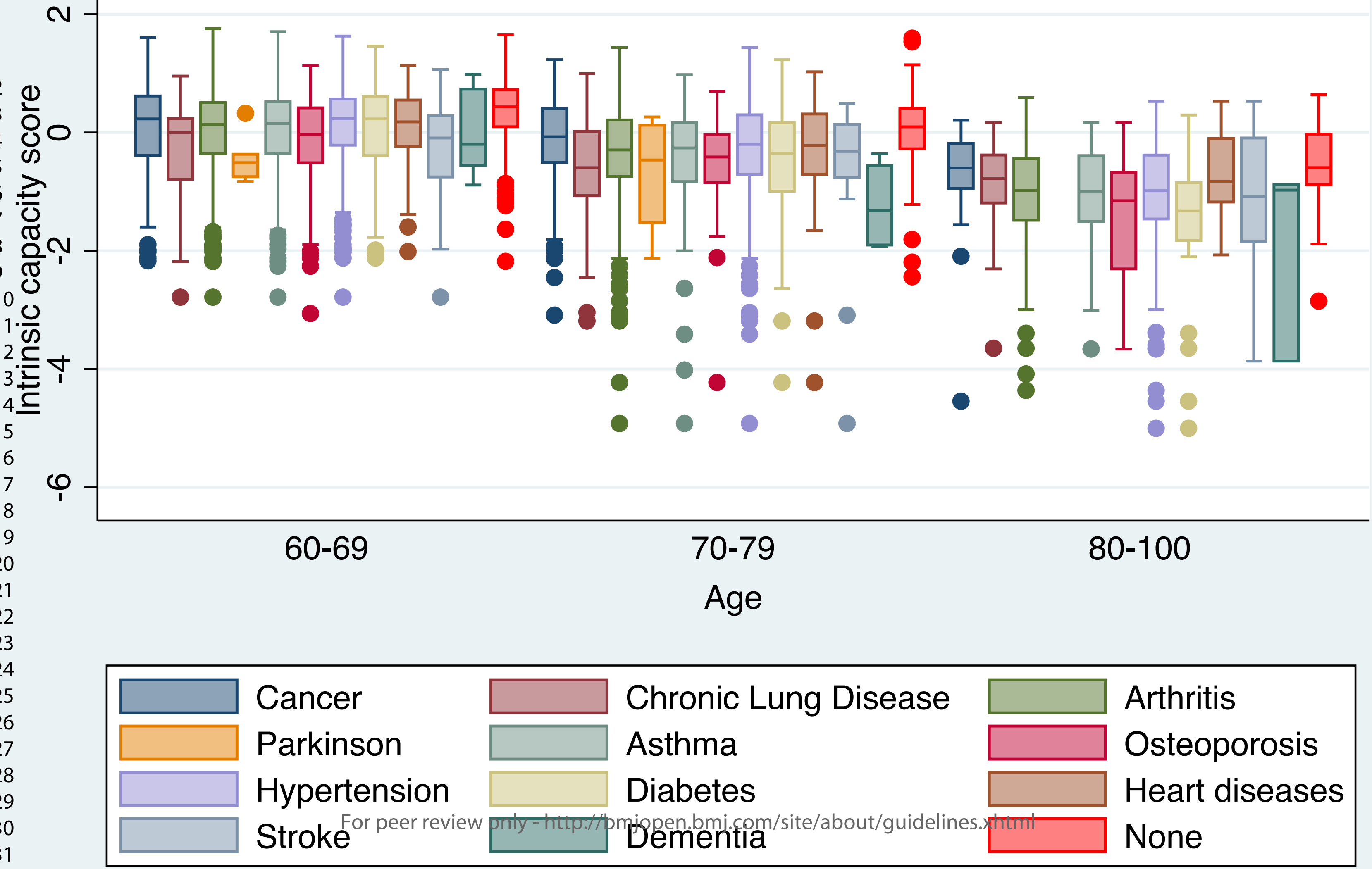

For peer review only - http://bmjopen.bmj.com/site/about/guidelines.xhtml

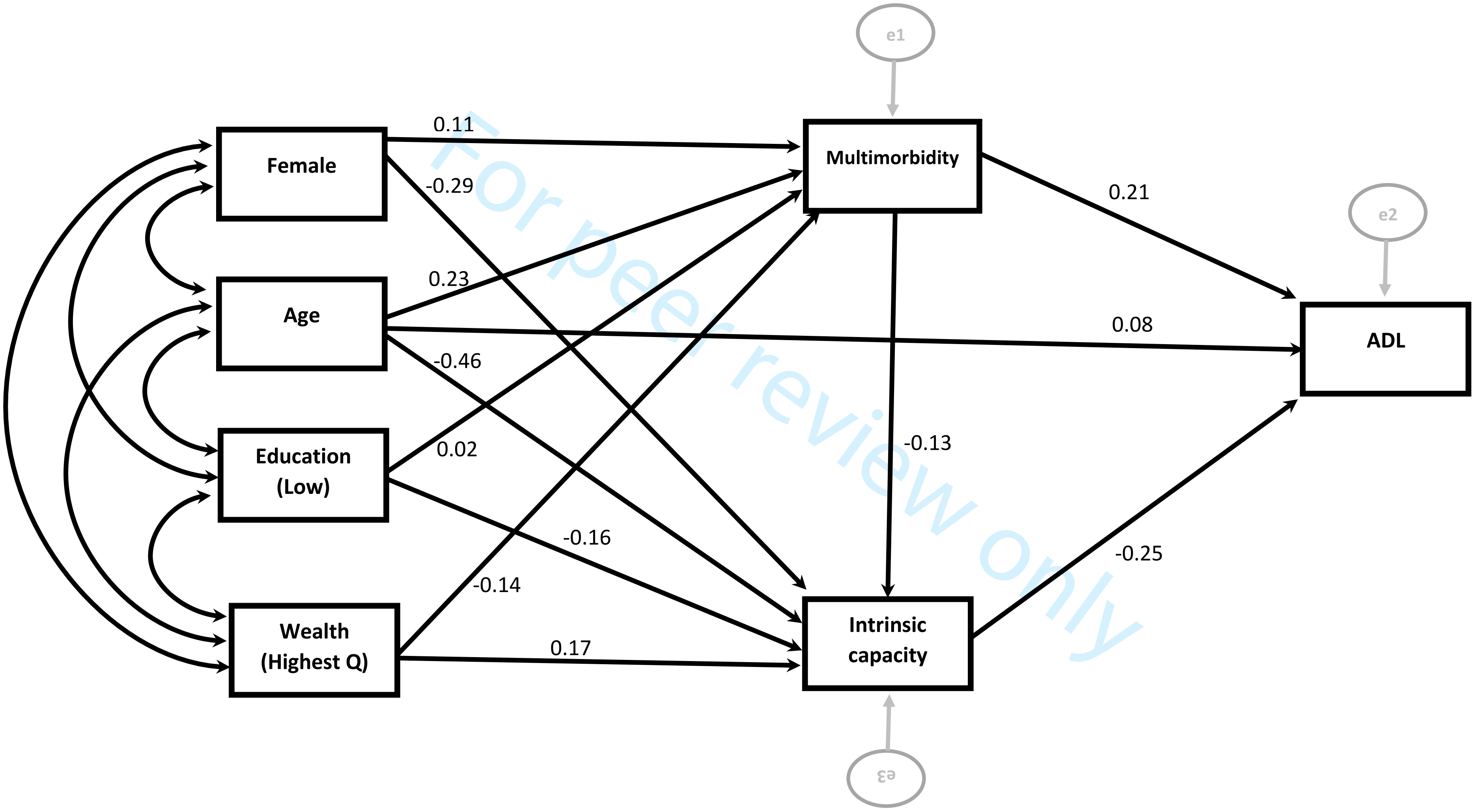

For peer review only

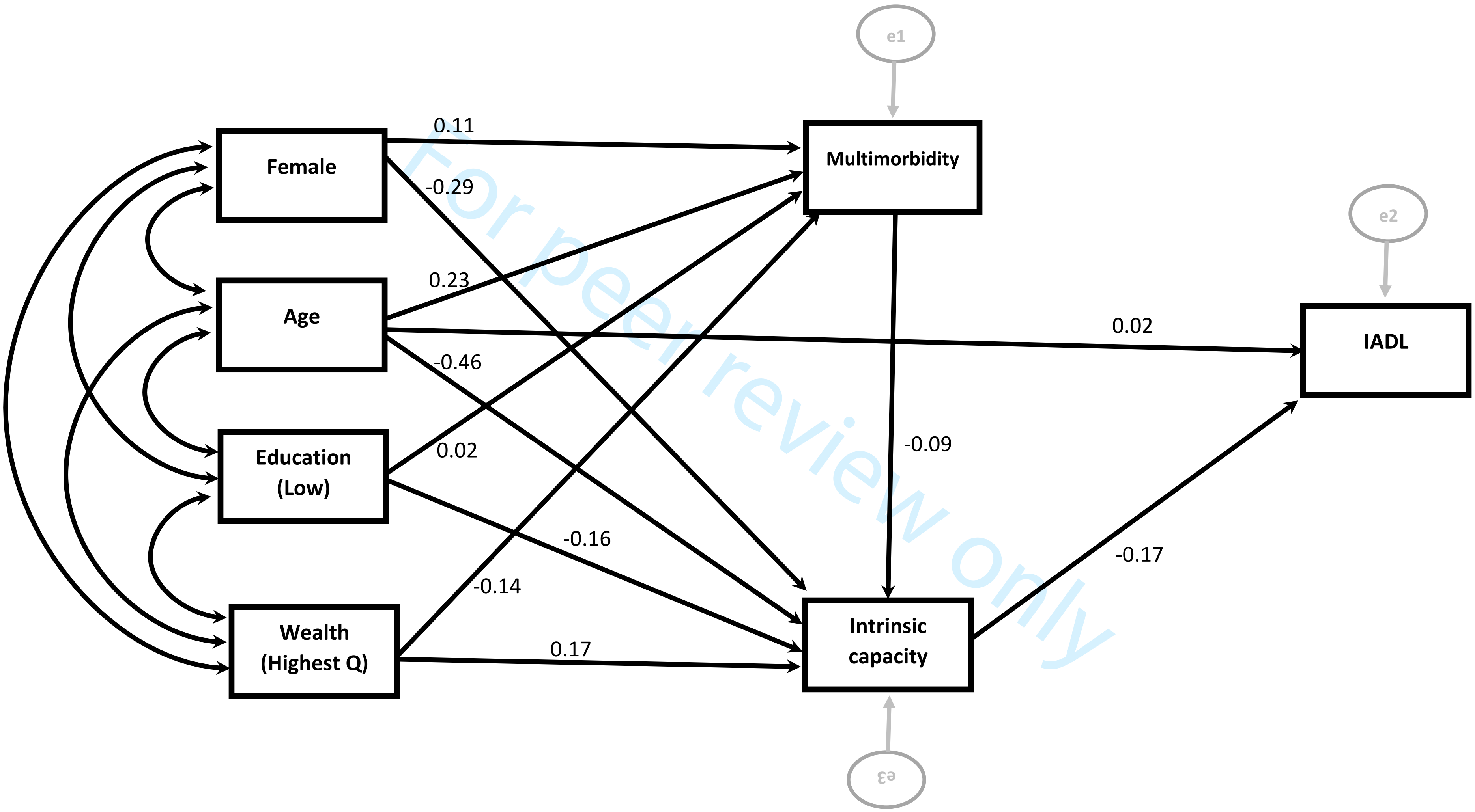

For peer review only

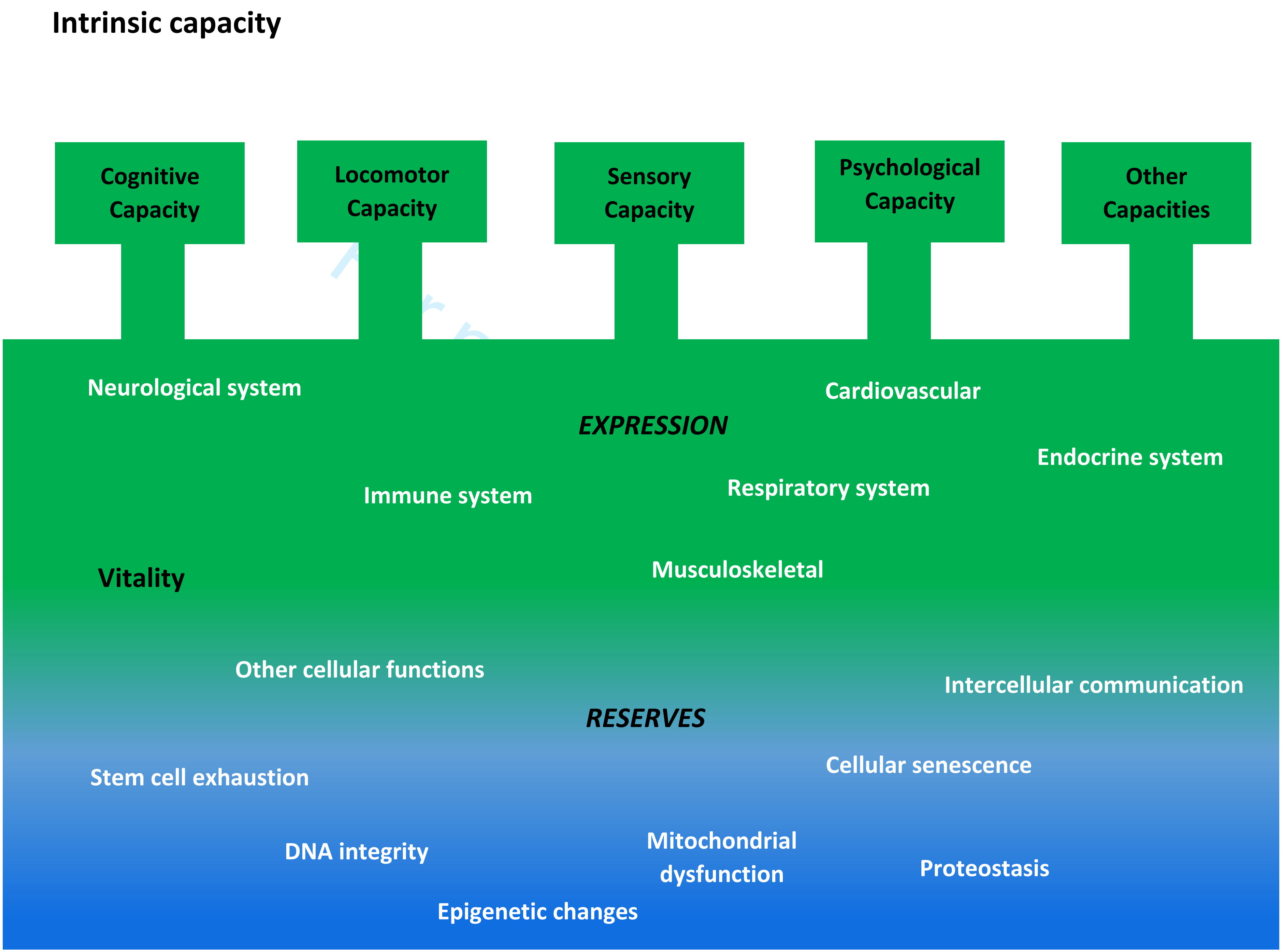
Intrinsic capacity
Cognitive Capacity
Locomotor Capacity
Sensory Capacity
Psychological Capacity
Other Capacities
Neurological system
Cardiovascular
EXPRESSION
Endocrine system
Immune system
Respiratory system
Vitality
Musculoskeletal
Other cellular functions
Intercellular communication
RESERVES
Stem cell exhaustion
Cellular senescence
DNA integrity
Mitochondrial dysfunction
Proteostasis
Epigenetic changes

Supplementary analysis

Table 1s : Sample characteristics

**Demographic and health characteristics**	**Wave 4**
**Age , mean (SD)**	70.5 (7.9)
**Sex**	
Male	3181(44.7%)
Female	3925 (55.3%)
**Education**	
No education	2035(30.7%)
Intermediate	2527(38.2%)
Higher education	2053(31.0%)
**Wealth quintile**	
1 (lowest)	712 (12.9%)
2	1119(20.3%)
3	1192 (21.6%)
4	1184(21.4%)
5 (highest)	1302(23.6%)
**Multimorbidity**	
0	1681(25.1%)
1 or 2	3647(54.3%)
3 or more	1381(20.6%)
**Activities of daily living**	
No ADL limitation	5745(80.8%)
1 or 2 limitations	1082(15.2%)

For peer review only

3 or more limitations	280 (3.9%)
**Instrumental activities of daily living**	
No limitation	5582(78.5%)
1 or 2 limitations	1228 (17.3%)
3 or more limitations	297 (4.2%)

Table 2s : Model fit statistics of ESEM and CFA models

Models	Fit statistics				
	χ2	df	CFI	TLI	REMSEA (90% CI)
**ESEM**					
One-factor	5385.6	104	0.63	0.57	0.136 (0.133 -0.139)
Two-factors	2570.8	89	0.82	0.76	0.101(0.098- 0.104)
Three-factors	927.5	75	0.94	0.90	0.064(0.061-0.068)
Four- factors	277.4	62	0.98	0.97	0.036(0.031-0.040)
Five-factors	117.9	50	0.99	0.98	0.022(0.017-0.030)
Six-factors (one general factor and five sub-factors) [1]	71.2	39	0.99	0.99	0.012(0.011-0.024)
**CFA**					
One-factor	6735.9	104	0.56	0.49	0.154(0.150-0.150)
Second-order	2369.9	102	0.94	0.92	0.073(0.070-0.080)
Correlated five factors	1782.3	103	0.95	0.92	0.060(0.050-0.060)
Bi-factor (one general factor and five sub-factors)	1180.6	89	0.98	0.97	0.035(0.033 -0.037)

[1] Bi-factor exploratory analysis is conducted in SEM framework.

For peer review only

Table 3s: Construct validity of intrinsic capacity and sub-domain score

Demographic and health characteristics	Intrinsic capacity Regression coefficient (95% CI)	Sensory Regression coefficient (95% CI)	Cognitive Regression coefficient (95% CI)	Vitality Regression coefficient (95% CI)	Psychological Regression coefficient (95% CI)	Locomotor Regression coefficient (95% CI)
**Age**	-0.052 (-0.054 to - 0.046)***	-0.002(-0.008 to 0.003)	-0.02(-0.023 to -0.019)***	-0.021(-0.024 to -0.019)***	-0.003(-0.005 to -0.0003)*	-0.009(-0.011 to -0.007)***
**Sex**						
Male	Ref 1	Ref 1	Ref 1	Ref 1	Ref 1	Ref 1
Female	-0.322 (-0.358 to -0.286)***	0.036(-0.037 to 0.110)	0.27(0.240 to 0.302)***	-0.881(-0.905 to -0.857)***	-0.251 (-0.314 to -0.189)***	0.099(0.059 to 0.139)***
**Education**						
No education	Ref1	Ref 1	Ref 1	Ref 1	Ref 1	Ref 1
Intermediate	0.462(0.420 to 0.504)***	0.085(0.037 to 0.133)***	0.267(0.230 to 0.304)***	0.068(0.029 to 0.106)***	0.027(-0-0.017 to 0.073)	0.058(0.028 to 0.094)***
Higher education	0.779(0.735 to 0.823)***	0.114(0.064 to 0.164)***	0.4042)***	0.239 (0.198 to 0.279)**3	-0.015(-0.062 to 0.032)	0.063(0.031 to 0.094)***
**Wealth quintile**						
1 (lowest)	Ref 1	Ref 1	Ref 1	Ref 1	Ref 1	Ref 1
2	-0.020(-0.085 to 0.044)	0.045(-0.025 to 0.116)	-0.072(-0.128 to -0.169)*	-0.072 (-0.132 to -0.129)*	-0.045(-0.113 to 0.230)	0.049(0.003 to 0.095)**
3	0.268(0.204 to 0.332)***	0.006(-0.150 to	0.050 (-0.005 to	0.016(-0.042 to	-0.017(-0.152 to	0.125(0.080 to 0.171)***

For peer review only

		0.163)	0.105)	0.075)	0.116)	
4	0.448(0.384 to 0.512)***	0.016(-0.141 to 0.164)	0.111(0.056 to 0.166)***	0.073 (0.014 to 0.131)*	-0.0530 (-0.119 to 0.0139)	0.155(0.110 to 0.200)***
5 (highest)	0.616(0.553 to 0.678)***	0.01(-0.142 to 0.159)	0.201 (0.147 to 0.255)***	0.099(0.041 to 0.156)**	0.055 (-0.074 to 0.184)	0.181(0.137 to 0.226)***
**Multimorbidity**						
0	Ref 1	Ref 1	Ref 1	Ref 1	Ref 1	Ref 1
1 or 2	-0.0237 (-0.279 to -0.195)***	-0.068(-0.151 to 0.014)	-0.012(-0.049 to 0.025)	-0.122(-0.160 to -0.085)***	0.035(-0.010 to 0.077)	-0.032(-0.061 to -0.003)*
3 or more	-0.764(-0.816 to -0.712)***	-0.057 (-0.151 to 0.014)	-0.067(-0.114 to -0.021)**	-0.308 (-0.354 to -0.261)***	-0.242(-0.350 to -0.133)***	-0.221(-0.251 to -0.185)***
**Activities of daily living**						
No ADL limitation	Ref 1	Ref 1	Ref 1	Ref 1	Ref 1	Ref 1
1 or 2 limitations	--0.471(-0.510 to -0.432)***	-0.105(-0.147 to 0.063)***	-0.049(-0.082 to -0.016)**	-0.096 (-0.131 to -0.061)***	-0.134 (-0.187 to -0.082)***	-0.097(-0.122 to -0.072)***
3 or more limitations	-0.857(-0.918 to -0.797)***	-0.116(-0.182 to -0.050)***	-0.088(-0.082 to -0.016)**	-0.072(-0.127 to -0.018)***	-0.028(-0.357 to 0.299)	-0.317 (-0.356 to -0.279)***
**Instrumental activities of daily living**						
No limitation	Ref 1	Ref 1	Ref 1	Ref 1	Ref 1	Ref 1
1 or 2 limitations	-0.636(-0.677 to -0.596)***	-0.097(-0.142 to -	-0.077(-0.111 to -	-0.127(-0.164 to -	-0.105(-0.253 to	-0.147 (-0.173 to -

		0.053)***	0.042)***	0.091)***	0.043)	0.121)***
3 or more limitations	-1.067(-1.144 to -0.990)***	-0.108(-0.193 to -0.023)**	-0.396 (-0.462 to -0.330)***	-0.107(-0.177 to -0.036)**	0.273(-0.108 to 0.656)	-0.259 (-0.309 to -0.209)***

*** p value < 0.001, **p value <0.05.

Table 4s: Regression coefficient of direct of intrinsic capacity on ADL and IADL and indirect effect through multimorbidity

Causal path	ADL		IADL	
	**Standardized Coefficient (SE)1**	**P value**	**Standardized Coefficient (SE)1**	**P value**
**Total effect**				
Intrinsic capacity	**-0.52 (0.01)**	**<0.001**	**-0.48(0.02)**	**<0.001**
**Direct effect**				
Intrinsic capacity	**-0.40(0.03)**	**<0.001**	**-0.39(0.02)**	**<0.001**
**Indirect effect**				
Intrinsic capacity→Multimorbidity	**-0.039(0.005)**	**<0.001**	**-0.024(0.005)**	**<0.001**
	**R2=0.20, pvalue=<0.001**		**R2= 0.21, pvalue=<0.001**	

[1]Controlled for age, sex, education, and wealth

For peer review only

Table 5s: Direct of and indirect effect personal characteristics on ADL and IADL in serial multiple mediators (intrinsic capacity and multimorbidity)

	ADL		IADL	
**Causal path**	**Standardized Coefficient (SE)**	**P value**	**Standardized Coefficient (SE)**	**P value**
**Direct effect**				
Age	0.006(0.02)	0.011	0.110 (0.03)	0.001
Sex	-0.049(0.03)	0.061	-0.049(0.02)	0.051
Education	0.009(0.01)	0.571	-0.012(0.02)	0.613
Wealth	-0.013(0.01)	0.185	0.016(0.24)	0.510
Multimorbidity	0.036(0.01)	0.001	0.020(0.02)	0.402
Intrinsic capacity	-0.099(0.02)	<0.000	-0.142(0.02)	<0.001
**Specific indirect effect**				
Age → Intrinsic capacity	0.053(0.01)	<0.001	0.050(0.01)	<0.001
Age → multimorbidity	0.011(0.04)	0.004	0.003(0.01)	0.405
Female → intrinsic capacity	0.047(0.010)	<0.001	0.048(0.01)	<0.001
Female → multimorbidity	0.008(0.003)	0.008	0.002(0.003)	0.409
Education→intrinsic capacity	--0.022(0.01)	<0.001	-0.02(0.01)	0.001
Education→multimorbidity	0.000(0.002)	0.810	0.00(0.00)	0.817
Wealth →intrinsic capacity	-0.021(0.01)	<0.001	-0.021 (0.01)	<0.001
Wealth→multimorbidity	-0.009(0.01)	0.007	-0.002(0.001)	0.408
**Indirect effect**				
Age → Multimorbidity → Intrinsic capacity	0.002(0.001)	0.002	0.002(0.001)	0.002
Female → Multimorbidity → Intrinsic capacity	0.002(0.001)	0.004	0.002(0.001)	0.004
Education→ Multimorbidity → Intrinsic capacity	0.000(0.00)	0.810	0.000(0.00)	0.810
Wealth → Multimorbidity → Intrinsic capacity	-0.002(0.001)	0.003	-0.002(0.001)	0.003

For peer review only

Figure 1s: A statistical diagram of a simple mediation model (Direct and indirect) effect of intrinsic capacity on ADL and IADL

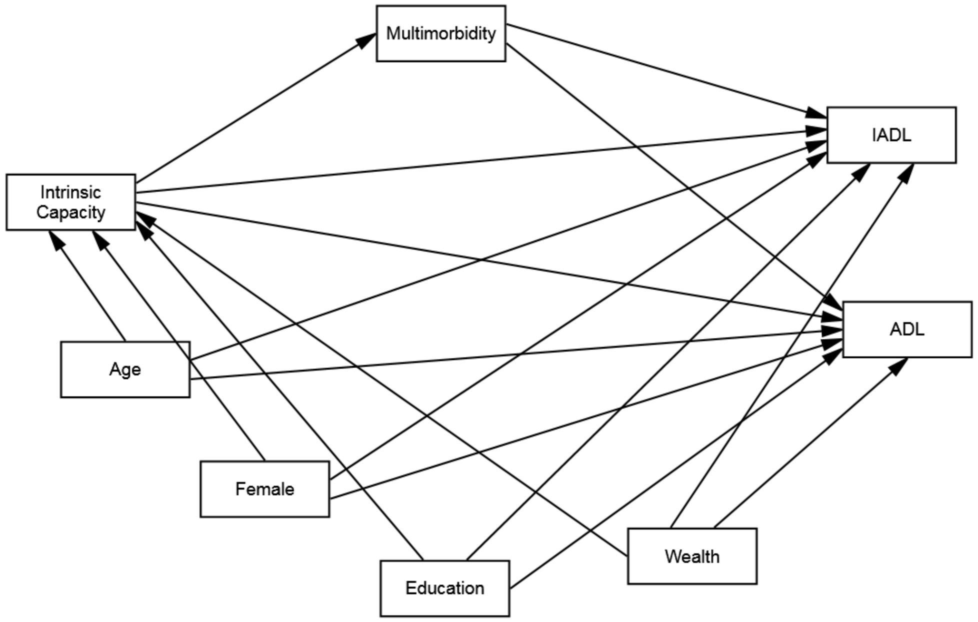

For peer review only

Table 6s: Model fit information for Parallel and Sequential models examining pathways to ADL and IADL

Models	Fit statistics					R2		
**Activities of daily livings**	χ2	df	CFI	TLI	REMSEA (90% CI)	**Multi morbi dity**	**Intrinsi c capacity**	**ADL**
Model 1a : Effect of age (and other covariates) on ADL is either mediated by multimorbidity or intrinsic capacity	43.9	5	0.96	0.89	0.06(0.04-0.08)	7.6%	42.6%	18.0%
Model 1b : Effect of age(other covariates) on ADL is mediated by multimorbidity and intrinsic capacity	16.3	4	0.98	0.96	0.04(0.022 -0.062)	7.6%	45.5%	18.9%
**Instrumental Activities of Daily Livings**								
Model 2a: Effect of age (and other covariates) on IADL is either mediated by multimorbidity or intrinsic capacity	55.6	5	0.95	0.86	0.07(0.05-0.09)	7.8%	42.7%	35.4%
Model 2b : Effect of age(and other covariates) on IADL is mediated by multimorbidity and intrinsic capacity	30.9	4	0.97	0.91	0.05(0.04-0.08)	7.9%	45.4%	31.2%
Model 2c : Effect of age(and other covariates) on IADL is mediated by intrinsic capacity (with no direct path between multimorbidity and IADL) model)	493	4	0.99	0.99	0.05(0.03-0.06)	11.5%	42%	32%

For peer review only

Figure 2s : Interaction effect of age and intrinsic capacity on incidence IADL.

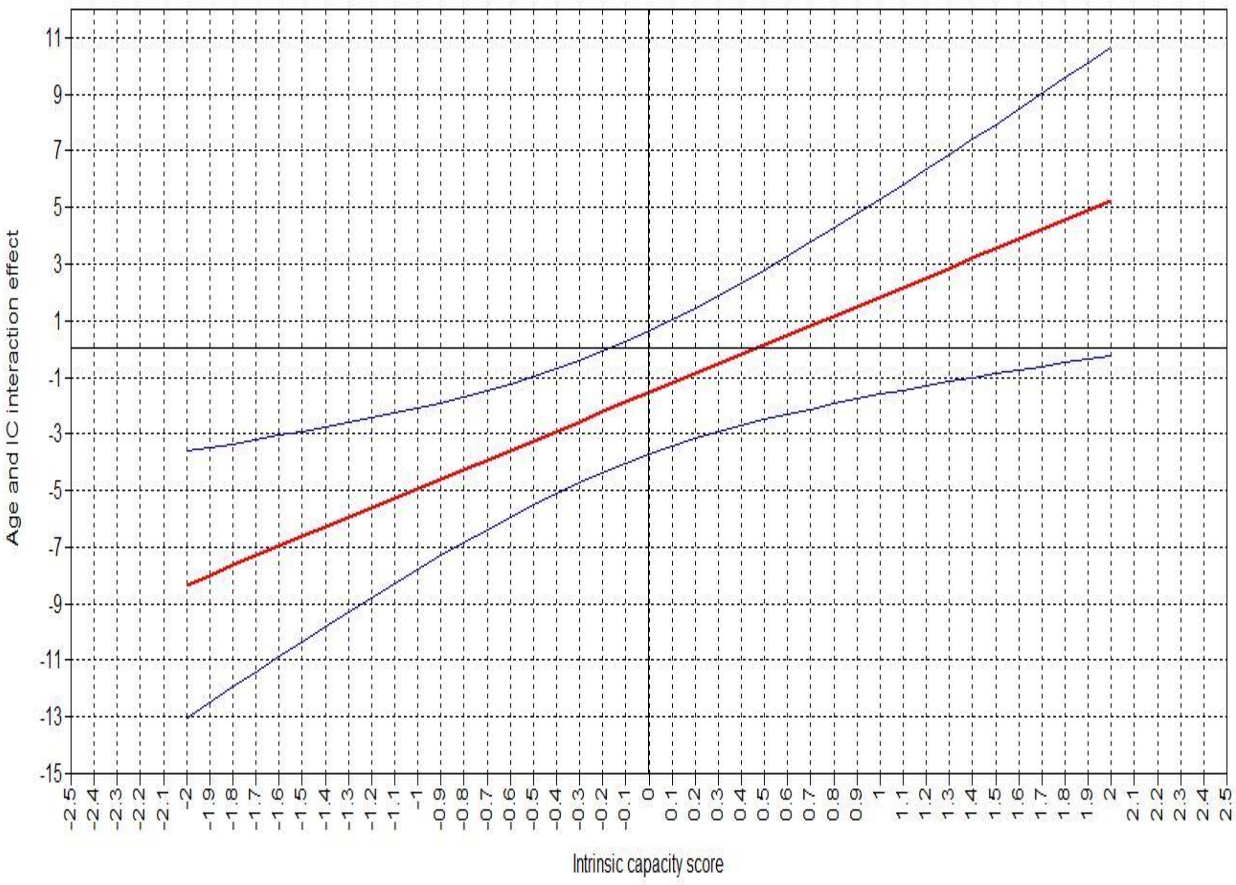

STROBE Statement—checklist of items that should be included in reports of observational studies

	Item No	Recommendation
**Title and abstract**	1	(*a*) Indicate the study's design with a commonly used term in the title or the abstract
		(*b*) Provide in the abstract an informative and balanced summary of what was done and what was found
**Introduction**		
Background/rationale	2	Explain the scientific background and rationale for the investigation being reported
Objectives	3	State specific objectives, including any prespecified hypotheses
**Methods**		
Study design	4	Present key elements of study design early in the paper
Setting	5	Describe the setting, locations, and relevant dates, including periods of recruitment, exposure, follow-up, and data collection
Participants	6	(*a*) *Cohort study*—Give the eligibility criteria, and the sources and methods of selection of participants. Describe methods of follow-up<br>*Case-control study*—Give the eligibility criteria, and the sources and methods of case ascertainment and control selection. Give the rationale for the choice of cases and controls<br>*Cross-sectional study*—Give the eligibility criteria, and the sources and methods of selection of participants
		(*b*) *Cohort study*—For matched studies, give matching criteria and number of exposed and unexposed<br>*Case-control study*—For matched studies, give matching criteria and the number of controls per case
Variables	7	Clearly define all outcomes, exposures, predictors, potential confounders, and effect modifiers. Give diagnostic criteria, if applicable
Data sources/ measurement	8*	For each variable of interest, give sources of data and details of methods of assessment (measurement). Describe comparability of assessment methods if there is more than one group
Bias	9	Describe any efforts to address potential sources of bias
Study size	10	Explain how the study size was arrived at
Quantitative variables	11	Explain how quantitative variables were handled in the analyses. If applicable, describe which groupings were chosen and why
Statistical methods	12	(*a*) Describe all statistical methods, including those used to control for confounding
		(*b*) Describe any methods used to examine subgroups and interactions
		(*c*) Explain how missing data were addressed
		(*d*) *Cohort study*—If applicable, explain how loss to follow-up was addressed<br>*Case-control study*—If applicable, explain how matching of cases and controls was addressed<br>*Cross-sectional study*—If applicable, describe analytical methods taking account of sampling strategy
		(*e*) Describe any sensitivity analyses

Continued on next page

**Results**		
Participants	13*	(a) Report numbers of individuals at each stage of study—eg numbers potentially eligible, examined for eligibility, confirmed eligible, included in the study, completing follow-up, and analysed
		(b) Give reasons for non-participation at each stage
		(c) Consider use of a flow diagram
Descriptive data	14*	(a) Give characteristics of study participants (eg demographic, clinical, social) and information on exposures and potential confounders
		(b) Indicate number of participants with missing data for each variable of interest
		(c) *Cohort study*—Summarise follow-up time (eg, average and total amount)
Outcome data	15*	*Cohort study*—Report numbers of outcome events or summary measures over time
		Case-control study—Report numbers in each exposure category, or summary measures of exposure
		Cross-sectional study—Report numbers of outcome events or summary measures
Main results	16	(*a*) Give unadjusted estimates and, if applicable, confounder-adjusted estimates and their precision (eg, 95% confidence interval). Make clear which confounders were adjusted for and why they were included
		(*b*) Report category boundaries when continuous variables were categorized
		(*c*) If relevant, consider translating estimates of relative risk into absolute risk for a meaningful time period
Other analyses	17	Report other analyses done—eg analyses of subgroups and interactions, and sensitivity analyses
**Discussion**		
Key results	18	Summarise key results with reference to study objectives
Limitations	19	Discuss limitations of the study, taking into account sources of potential bias or imprecision. Discuss both direction and magnitude of any potential bias
Interpretation	20	Give a cautious overall interpretation of results considering objectives, limitations, multiplicity of analyses, results from similar studies, and other relevant evidence
Generalisability	21	Discuss the generalisability (external validity) of the study results
**Other information**		
Funding	22	Give the source of funding and the role of the funders for the present study and, if applicable, for the original study on which the present article is based

*Give information separately for cases and controls in case-control studies and, if applicable, for exposed and unexposed groups in cohort and cross-sectional studies.

Note: An Explanation and Elaboration article discusses each checklist item and gives methodological background and published examples of transparent reporting. The STROBE checklist is best used in conjunction with this article (freely available on the Web sites of PLoS Medicine at http://www.plosmedicine.org/, Annals of Internal Medicine at http://www.annals.org/, and Epidemiology at http://www.epidem.com/). Information on the STROBE Initiative is available at www.strobe-statement.org.

For peer review only - http://bmjopen.bmj.com/site/about/guidelines.xhtml
